# Non-Targeted Plasma Lipidomic Profiling in Late Pregnancy and Early Postpartum Stages: An Observational Comparative Study

**DOI:** 10.3390/metabo15120798

**Published:** 2025-12-16

**Authors:** Alexandra Traila, Simona-Alina Abu-Awwad, Carmen-Ioana Marta, Manuela Violeta Bacanoiu, Anca Laura Maghiari, Ahmed Abu-Awwad, Marius Lucian Craina

**Affiliations:** 1 Doctoral School, “Victor Babes” University of Medicine and Pharmacy Timisoara, Eftimie Murgu Square 2, 300041 Timisoara, Romania; alexandra.traila@umft.ro; 2 Department of Obstetrics and Gynecology, County Emergency Hospital, 220056 Drobeta Turnu Severin, Romania; 3 Department of Obstetrics and Gynecology, “Victor Babes” University of Medicine and Pharmacy Timisoara, Eftimie Murgu Square 2, 300041 Timisoara, Romania; alina.abuawwad@umft.ro (S.-A.A.-A.); craina.marius@umft.ro (M.L.C.); 4 Clinic of Obstetrics and Gynecology, “Pius Brinzeu” County Clinical Emergency Hospital, 300723 Timisoara, Romania; 5 Sport Medicine and Physiotherapy Department, University of Craiova Romania, 200500 Craiova, Romania; 6 Department of Laboratory Medicine, County Clinical Emergency Hospital of Craiova, 200642 Craiova, Romania; 7 Department I—Discipline of Anatomy and Embryology, “Victor Babes” University of Medicine and Pharmacy, Eftimie Murgu Square, No. 2, 300041 Timisoara, Romania; boscu.anca@umft.ro; 8 Department XV—Discipline of Orthopedics—Traumatology, “Victor Babes” University of Medicine and Pharmacy, Eftimie Murgu Square, No. 2, 300041 Timisoara, Romania; ahm.abuawwad@umft.ro; 9 Research Center University Professor Doctor Teodor Șora, “Victor Babes” University of Medicine and Pharmacy, Eftimie Murgu Square, No. 2, 300041 Timisoara, Romania

**Keywords:** lipidomic, biomarkers, HPLC-QTOF-ESI^+^-MS, multivariate analysis

## Abstract

**Background/Objectives**: Pregnancy represents a unique physiological state marked by extensive metabolic adaptations, particularly in lipid pathways essential for maternal adjustments, fetal development, and postpartum recovery. This study aimed to explore these changes through untargeted lipidomic profiling. **Methods**: This observational, comparative, non-interventional clinical study included 107 women, of which 65 were in the third trimester of pregnancy (mean age 27.9 ± 5 years) and 42 were in the early postpartum period (≤7 days, mean age 28.9 ± 5.9 years). Inclusion criteria were singleton, term pregnancies (37–41 weeks) with neonates weighing > 2500 g and no associated pregnancy-related pathologies; exclusion criteria included multiple gestation, use of lipid-altering medications, maternal age > 40 years, or diagnosed pregnancy complications. Plasma samples were analyzed using High-Performance Liquid Chromatography–Quadrupole Time-Of-Flight–Electrospray Ionization (positive mode)–Mass Spectrometry, data were processed with MetaboAnalyst 6.0 using multivariate and univariate analyses (Partial Least Squares–Discriminant Analysis, Volcano Plot, Random Forest, Receiver Operating Characteristic analysis), with statistical significance set at *p* < 0.05. **Results**: Multivariate analysis demonstrated a clear separation between groups with high predictive accuracy as reflected by strong classification metrics (Accuracy = 0.90, R^2^ = 0.75, Q^2^ = 0.68). Several discriminative lipids were consistently identified across statistical models, including 2-Methoxyestrone (AUC = 0.861), Eicosanedioic acid (AUC = 0.854), and Pregnenolone sulfate (AUC = 0.843). These biomarkers were further categorized into five major lipid classes: steroid hormones, long-chain fatty acids, lysophospholipids, ceramides/sphingolipids, and glycerolipids. **Conclusions**: Untargeted lipidomic profiling revealed distinct metabolic signatures that differentiate late pregnancy from early post-partum states. The identification of robust lipid biomarkers with high discriminative performance highlights their potential utility in maternal health monitoring, obstetric risk assessment, and postpartum recovery surveillance.

## 1. Introduction

Pregnancy is a complex physiological state, characterized by coordinated adaptations across multiple body systems. Although many adaptive changes have been described, an overall physiological picture remains difficult to define because pregnancy is dynamic and shaped by continuous maternal–fetal interactions [1]. Among the systems most extensively remodeled is lipid metabolism, which supports the energetic, structural, and hormonal demands of gestation. Lipid molecules are not only crucial for energy storage and membrane integrity but also serve as bioactive mediators in inflammation, vascular remodeling, and steroid hormone biosynthesis [2].

Alterations in lipid metabolism have been increasingly linked to obstetric syndromes such as gestational hypertensive disorders (GHD) [3,4], preeclampsia (PE) [5,6,7,8], gestational diabetes mellitus (GDM) [9,10,11,12], preterm birth [13,14], and abnormal fetal growth [15,16]. Understanding these metabolic shifts may provide valuable insights into the pathophysiology of pregnancy complications and reveal potential biomarkers for early risk stratification.

Emerging metabolomic and lipidomic studies have highlighted significant gestational progression–related changes in specific lipid classes and their association with neonatal anthropometry. Song et al. demonstrated longitudinal associations between lipid subclass variations and newborn size parameters, suggesting potential biomarkers for fetal growth monitoring [17,18,19]. Similarly, Handelman et al. reported dynamic changes in plasma metabolites from early to late pregnancy, notably increases in steroid conjugates such as estriol 3-sulfate, underscoring the endocrine remodeling that occurs throughout gestation [20,21]. Complementary studies by Orczyk-Pawilowicz et al. revealed marked metabolic differences between trimesters in both amniotic fluid and maternal plasma, emphasizing shifts in carbohydrate and lipid intermediates [22]. Collectively, these findings underscore the role of lipidomic remodeling in pregnancy and the need for robust biomarkers capable of detecting deviations from physiological adaptation [2,23].

Longitudinal analyses have also demonstrated coordinated shifts in fatty acids, acylcarnitines, and amino acids across gestation [24]. However, the early postpartum period, despite its importance for maternal recovery and metabolic reequilibration, has been comparatively less studied. Postpartum lipid metabolism may influence both short-term recovery and long-term cardiometabolic health. For instance, Tinius et al. showed that postpartum lipidomic patterns are associated with weight retention and metabolic risk [25], while Yu et al. identified metabolomic signatures linked to postpartum depression [26]. Despite these advances, few studies have directly compared maternal lipidomic profiles between late pregnancy and early postpartum.

Given these gaps, the objective of this study was understanding the maternal adaptation and identify potential biomarkers for obstetric monitoring.

Therefore, we hypothesized that the maternal plasma lipidome undergoes systematic, quantifiable shifts from the third trimester of pregnancy to the early postpartum period (≤7 days).

## 2. Materials and Methods

### 2.1. Study Population and Sample Collection

This observational, comparative, non-interventional clinical study enrolled 66 pregnant women in the third trimester and 42 early postpartum (≤7 days after delivery) who met the inclusion criteria. One of the participants left the study, so the total number of pregnant women decreased to 65. All consecutive eligible women attending the Obstetrics and Gynecology Department from Timisoara, Romania, during the recruitment period (December 2023–April 2024) were included in the study, resulting in a final cohort of 108 participants. The final cohort of 108 participants provided a statistical power of approximately 71% to detect moderate effect sizes (Cohen’s d = 0.5) between groups, at a two-sided α = 0.05. This investigation was designed as an exploratory study. Subjects were recruited from the Obstetrics and Gynecology Departments of the Emergency Clinical Hospital “Pius Branzeu” Timisoara, during routine antenatal or postpartum care. To reduce the risk of selection bias, all consecutive eligible women attending the Obstetrics and Gynecology Department during the recruitment period (September–December 2023) were enrolled. Inclusion and exclusion criteria were predefined and uniformly applied, and no selective recruitment was performed. All procedures were performed in accordance with research ethical standards and approved by institutional ethics committees. Present study was conducted in accordance with all ethical standards research and is in accordance with the 1964 Declaration of Helsinki [27] and its later amendments. The study was approved by the Ethical Commission for Scientific Research of “Victor Babeș” University of Medicine and Pharmacy Timisoara and approved with number 28 on 1 September 2023. This study was also approved by the Ethics Committee of the Emergency County Hospital “Pius Branzeu” in Timisoara, with number 424 of 8 December 2023. Written informed consent was obtained from all participants prior to sample collection. Eligible women were 19–40 years of age. We restricted the cohort to singleton term deliveries (37–41 weeks) with appropriate-for-gestational-age neonates (birth weight > 2500 g) and uncomplicated outcomes. Exclusion criteria included multiple gestation, use of lipid-altering medications, maternal age > 40 years, and diagnosed pregnancy pathologies. A post hoc power analysis was performed to assess the adequacy of the sample size. Based on the main discriminatory lipid features identified by multivariate modeling (PLS-DA and Random Forest), the achieved statistical power exceeded 0.80 at an alpha level of 0.05 for large effect sizes (Cohen’s d > 0.8). This indicates that the study had sufficient power to detect meaningful differences between the pregnancy and postpartum groups, supporting the reliability of the results despite the moderate cohort size.

Peripheral venous blood (4–5 mL) was collected in the morning (08:00–10:00 a.m.) after overnight fasting, using K_2_-EDTA vacutainers (Timișoara, România). Participants refrained from caffeine, alcohol, and lipid-modifying drugs for at least 72 h prior to sampling; none were under hormonal or lipid-altering medication. Samples were centrifuged within 30 min (2000× *g*, 15 min, 4 °C), and plasma aliquots were stored at −80 °C until analysis. Ethical approval was obtained from the institutional review boards (protocols 28/2023 and 424/2023).

Peripheral venous blood was collected in EDTA tubes at enrollment (G: third trimester; L: ≤7 days postpartum). Plasma was separated immediately by centrifugation at 4 °C for 15 min at 2000× *g*. The samples were stored at −80 °C in the Biological Materials Bank of the “Victor Babes” University of Medicine and Pharmacy in Timisoara.

### 2.2. Plasma Sample Preparation

To 0.2 mL of plasma from each sample was added 0.8 mL of a mixture of solvents (methanol–acetonitrile–tert-butyl ether, 1:1:0.25). The mixture was shaken for 30 s, then stored at −20 °C for 24 h to precipitate the protein. The samples were thawed and centrifuged at 12,500× *g* for 10 min. The supernatant was collected, filtered through 0.2 µm PTFE filters and transferred to autosampler vials for metabolomic analysis. This mixture of solvents was used to improve the extraction of lipid components.

### 2.3. HPLC-QTOF-ESI+MS Instrumentation and Analysis

Target analytes were characterized using high-performance liquid chromatography (HPLC) coupled to quadrupole time-of-flight mass spectrometry (QTOF-ESI^+^-MS). The analytical platform consisted of a Thermo Scientific UltiMate 3000 HPLC system (Cluj-Napoca, Romania), equipped with a Dionex Ultimate quaternary pump, column oven, and autosampler, directly interfaced with a MaXis Impact QTOF mass spectrometer (Bruker Daltonics, Bremen, Germany). The separation was carried out on a reverse-phase C18 column (Kinetex UPLC C18, 5 µm, 4.6 × 150 mm; Phenomenex, Torrance, CA, USA) maintained at a constant temperature of 25 °C. The mobile phase consisted of two eluents: (A) ultrapure water with 0.1% formic acid (*v*/*v*), and (B) a ternary solvent mixture of methanol–acetonitrile–isopropanol (1:1:1, *v*/*v*/*v*), also containing 0.1% formic acid. The gradient elution program was defined as follows: 70% A at 0 min, decreasing to 30% A at 4 min, then to 0% A at 7 min, subsequently returning to 30% A at 10 min, and finally to 70% A at 13 min, maintained until the end of the run at 15 min. The flow rate was 0.8 mL/min, and the injection volume was 25 µL. Each sample was analyzed in duplicate. In cases where significant differences between replicates were observed, a third injection was performed, and the result was calculated as the average of consistent values. Analytes eluted from the column were ionized using electrospray ionization in positive mode (ESI^+^) and Sodium formate 10 mM was used to generate positively charged ion clusters under electrospray ionization (ESI^+^) conditions. The ionization conditions were as follows: nebulizer gas pressure (nitrogen) 2.8 bar, drying gas flow rate 12 L/min, and drying temperature 300 °C. Full-scan acquisition was performed over an *m*/*z* range of 100–1000 Da, capturing both low and mid-mass molecular ions with high resolution and accuracy.

To ensure the reliability, reproducibility, and analytical robustness of the HPLC-ESI^+^-QTOF-MS system throughout the study, a structured quality control (QC) protocol was implemented in parallel with regular instrument calibration. QC samples (obtained by mixing 0.2 mL plasma from each sample, in each group) were run repeatedly and after each 10 samples, to assure a high reproducibility of data. All QC solutions were prepared using the same solvents as the analytical samples, filtered (0.22 µm PTFE), and stored at 4 °C before analysis. System suitability was considered acceptable if the mass error of QC compound ions was ≤±5 ppm; retention time deviation was within ±0.2 min; signal intensity (peak area) for each QC compound varied less than 20% relative standard deviation (RSD) across the batch. Each sample was analyzed in duplicate. Also, as Internal standard (solution of Doxorubicin hydrochloride 2 mg/mL) used to allow for normalization and control of the entire workflow, improving the accuracy of relative metabolite abundance comparisons.

The mass accuracy and retention time reproducibility were monitored in real time using the instrument software (HyStar 3.2) and processed using Data Analysis 4.2. This approach ensured the generation of high-confidence data suitable for downstream quantitative and qualitative interpretation.

### 2.4. Data Processing and Statistical Analysis

**Figure 1:**
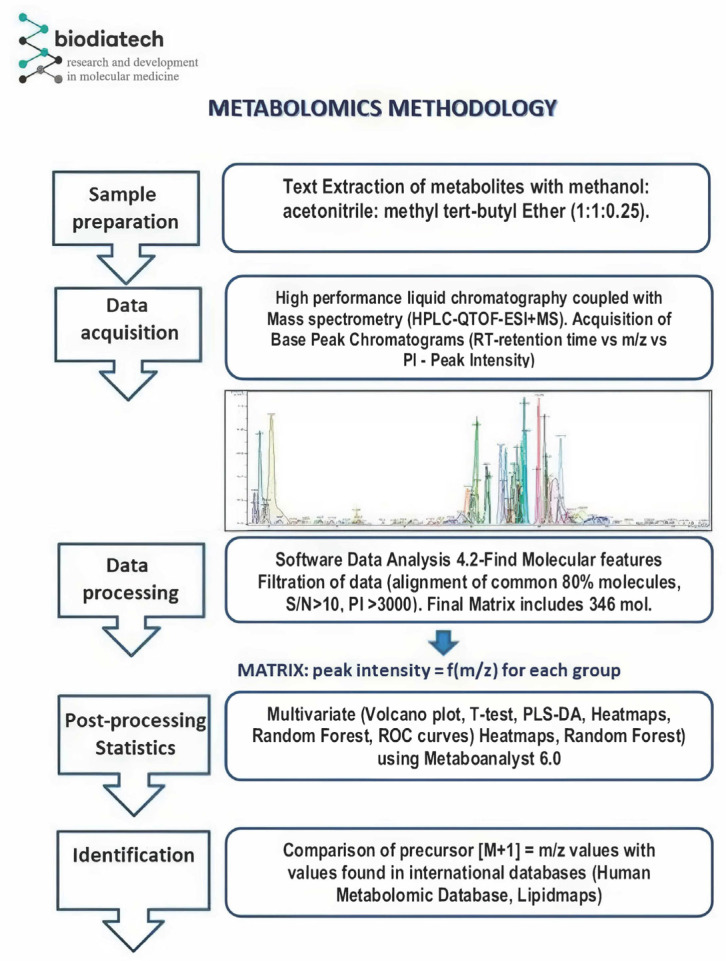
Workflow diagram for untargeted lipidomic data processing, features selection and metabolite annotation.

The general scheme of the workflow is presented in Figure 1. Data processing was performed using Data Analysis 4.2. software. In a first step (1) individual total ion chromatograms (TICs) were recorded and subsequently transformed into base peak chromatograms (BPCs). Compound MS spectra were obtained using the FMF (Find Molecular Features) function. The output table from the FMF matrix included the retention times, peak areas and peak intensities, and signal-to-noise (S/N) ratio for each component, along with their respective *m*/*z* values. Typically, the number of separated compounds ranged from 1100 to 1800 molecules. Molecules with retention times shorter than 1.6 min (corresponding to the void volume of the LC column) and peak intensities below 3000 were excluded, *m*/*z* values below 995 Da as were those with S/N values below 10.

In a second step (2) the matrices obtained were submitted to an alignment of retention times using the online software available at https://www.bioinformatics.org/bioinfo-af-cnr/NEAPOLIS/ (accessed on the 4 November 2024). After alignment, there were kept 80% of the common molecules identified in all samples, resulting in a matrix containing a number of 346 *m*/*z* values. This matrix (provided as Supplementary Materials, Files S1 and S2) was used for statistical analysis, uploaded as a .csv file to the Metaboanalyst 6.0 online platform (www.metaboanalyst.com).

In a third step (3), the statistical analysis was carried out. A combination of multivariate approaches were implemented to maximize both the classification power and the biological relevance of the findings. Lipidomic analysis of all plasma samples included the following algorithms: PLS-DA and loadings VIP scores-top 15 molecules, Volcano Plot (*p* < 0.1) and *t*-test (*p* < 0.05), Random Forest and Mean Decrease Accuracy (MDA) scores, Heatmap including dendrogram, Biomarker Analysis (BA) which includes the receiver operating characteristic (ROC) and area under the curve (AUC).

Partial Least Squares Discriminant Analysis (PLS-DA) was applied as a supervised learning algorithm with enhanced discriminative power comparative to PCA. Molecules with Variable Importance in Projection (VIP) scores > 1 were considered as most influential in differentiating between pregnant (G) and postpartum (L) groups [28,29].

Random Forest (RF) was applied as a classification method which provides a Mean Decrease Accuracy (MDA) score with a good prediction accuracy. Features with the highest MDA values are interpreted as the most critical in discriminating between G and L groups. This approach complements PLS-DA by confirming feature relevance through non-linear decision boundaries and robustness to overfitting [30].

Volcano Plot and *t*-test analysis visualized the relationship between magnitude of change (log2 fold-change) and statistical significance (−log10 *p*-value) for each feature. Student’s *t*-tests were performed for each metabolite to determine significant levels. Features with log2 fold-change greater than ±1 and adjusted *p*-values less than 0.05 were selected as differentially expressed lipids. This approach is particularly effective in highlighting molecules that are both statistically and biologically significant. Molecules passing a threshold of *p* < 0.05 and log2FC > ±1 were flagged for further annotation [31].

In a final step (4) the identification of molecules, the experimental *m*/*z* values being compared with the average of theoretical *m*/*z* values from the Human Metabolome Database (https://hmdb.ca/), from LIPID MAPS^®^ Lipidomics Gateway (https://www.lipidmaps.org/data/structure/LMSDSearch.php) (accessed on 4 November 2024), the accuracy of theoretical–experimental *m*/*z* values being set below 5 ppm. Using the HMDB and LipidMaps databases, 277 molecules were identified. The list of putative biomarkers (*n* = 277) identified in the plasma of two groups (pregnant and postpartum) from a total of 346 molecules selected as common ones in all groups, are presented in Supplementary Table S3.

## 3. Results

The results of this study reflect the outcome of comprehensive untargeted lipidomic profiling performed on plasma samples from pregnant women in the third trimester compared to postpartum women. Using a combination of advanced analytical techniques, several discriminative lipid biomarkers were identified that highlight significant metabolic alterations between the two physiological states.

### 3.1. Demographic and Clinical Characteristics of Study Participants

Baseline characteristics of the third-trimester (G) and early postpartum (L) cohorts are summarized in Table 1. Overall, the groups were comparable, with no statistically significant differences across continuous measures or categorical distributions (all *p* > 0.05); gestational age at sampling is reported only for the pregnant cohort.

### 3.2. PLS-DA (Partial Least Squares Discriminant Analysis)

**Figure 2:**
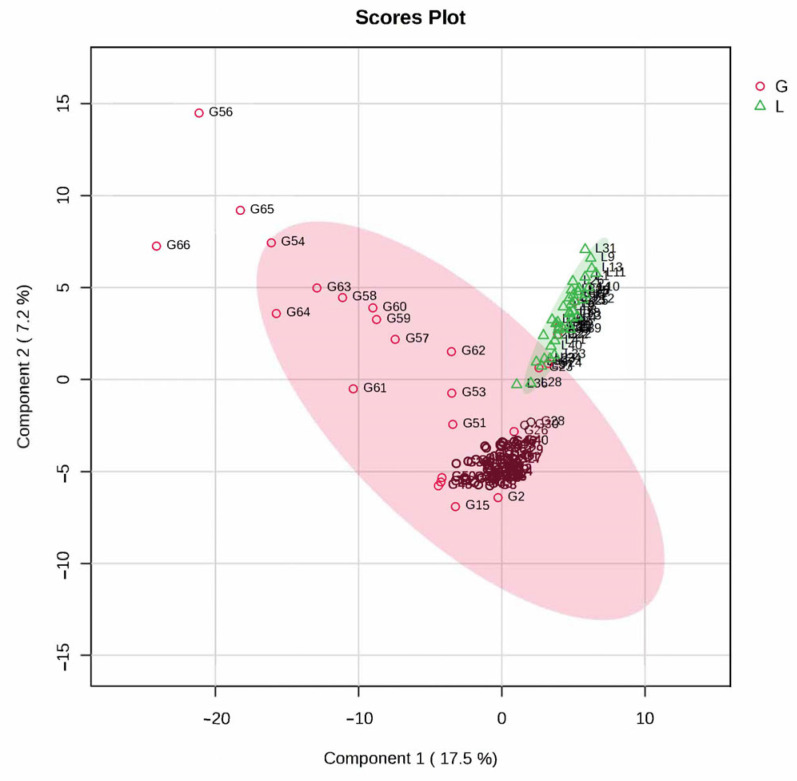
Partial least squares discriminant analysis (PLS-DA) plot shows a clear separation, the red area represents group G (pregnant) and the green area represents group L (postpartum).

Partial Least Squares Discriminant Analysis (PLS-DA) demonstrated a clear separation between groups G (pregnant) and L (postpartum), as illustrated in Figure 2. Visualization of the PLS-DA chromatogram generated by MetaboAnalyst 6 software highlights the separation of the groups. The PLS-DA model captured 17.5% of the variance in component 1 and 7.2% in component 2. The cross-validation algorithm indicated high accuracy (Accuracy = 0.9), adjustment parameter (R^2^ = 0.75) and prediction parameter (Q2 = 0.68) based on the first 5 components of the PLS-DA model. Pregnenolone sulfate, Docosadienoic acid FA 22:2, 2-Methoxyestrone, Eicosanedioic acid FA 20:1; O_2_, and Estrone 3-sulfate were the molecules with the highest VIP scores.

### 3.3. Volcano Plot and t-Test Analysis

**Figure 3:**
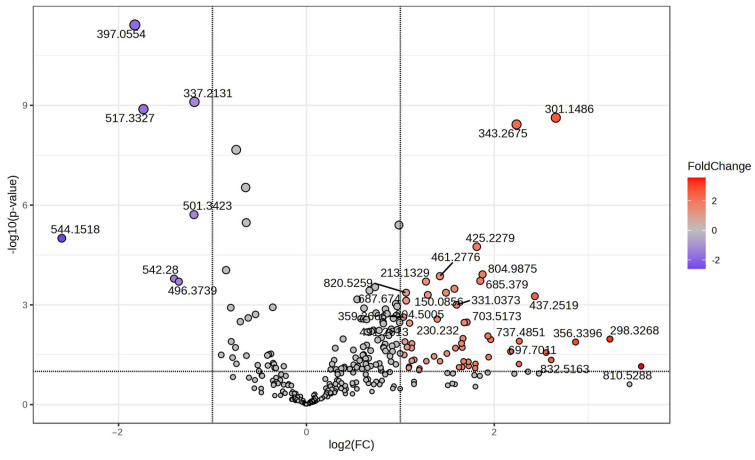
Volcano plot representing the *m*/*z* values of molecules with increase MS intensity levels in group L (log2FC < 0) and decrease levels (log2FC > 0) comparative to group G.

These tests highlighted features with both high statistical significance and substantial fold-change. The volcano plot displays the relationship between –log10 (*p*-value) and log2 fold-change (FC) for each molecule relative to the selected covariate. The x-axis represented the log2 fold change between groups, while the y-axis plotted the negative log10 of the adjusted *p*-value. Molecules with high statistical significance are in the upper area of the graph, above the horizontal line, while molecules with high differential expression are located to the left-right of the ±1 log2FC values. The horizontal line represents *p*-value threshold. Molecules were color-coded when the resulting *p*-value was below the specified threshold. The most statistically significant molecules are labeled in Figure 3. Molecules with increased intensity in group G are marked in orange, 2-Methoxyestrone, Eicosanedioic acid FA 20:1; O2 and in blue are the molecules with increased intensity in group L, respectively. Pregnenolone sulfate and Docosadienoic acid FA 22:2.

### 3.4. Random Forest

**Figure 4:**
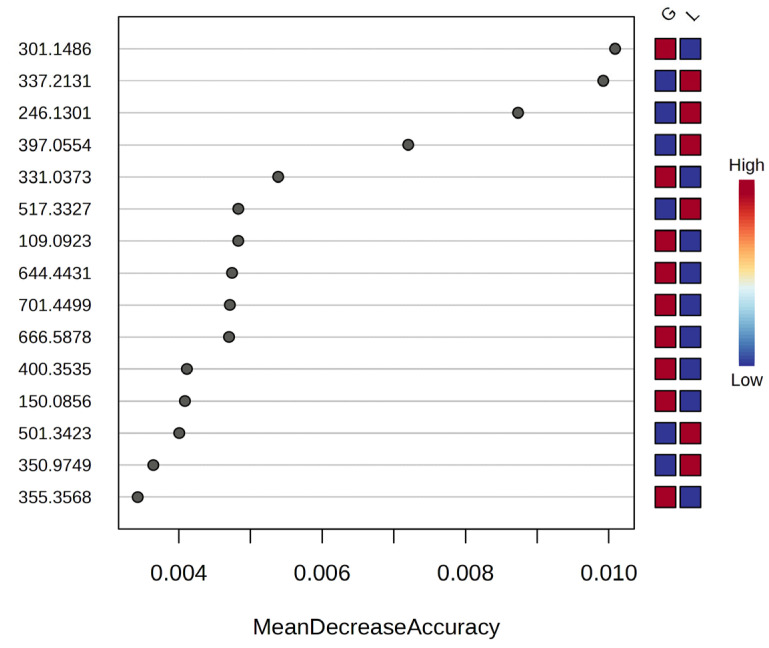
MDA values versus *m*/*z* of the first 15 molecules with discriminating power between the pregnant group (G) and the postpartum group (L), High-intensity molecules are represented in red, while low-intensity molecules are represented in blue, according to the Random Forest analysis.

Random Forest classification analysis further supported group separation and feature discrimination. The algorithm ranked the importance of the variables based on the mean decrease in accuracy (MDA). MDA values range from 0.01 to 0.003 and the increase or decrease in the level of these molecules in L Vs. G groups were considered. A higher MDA value indicates the importance of that metabolite in the differentiation pool. Figure 4 presents the top discriminating molecules, with 2-Methoxyestrone, Docosadienoic acid FA 22:2, Valeroylcarnitine, and Pregnenolone sulfate emerging as the most relevant for group classification. The model achieved an overall classification accuracy over of 90%, validating the consistency and biological relevance of the identified features.

### 3.5. Heatmap

**Figure 5:**
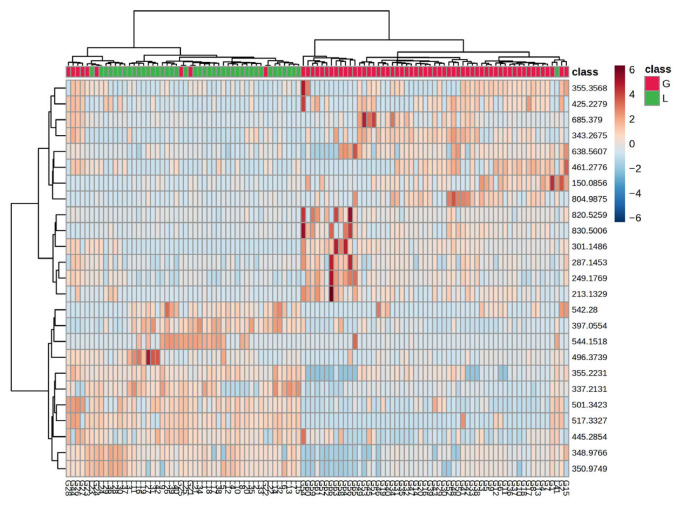
Heatmap representing correlations between samples and variables (*m*/*z* values), between groups G (pregnant) and L (postpartum). Correlations determined by the *t*-test are represented as follows: red areas show molecules that have increased levels, and blue areas show molecules with decreased levels for specified samples.

The heatmap generated with MetaboAnalyst 6.0 illustrates correlations between samples and the identified molecules (*m*/*z* values). The heatmap highlights significant differentiation between samples and features, represented by distinct color intensities. Colors represent the correlations: red-positive and blue-negative as determined by *t*-test. Hierarchical clustering of samples from groups G and L, as well as the heat map using the Euclidean distance measure and the Ward clustering algorithm is illustrated in Figure 5.

### 3.6. Receiver Operating Characteristic (ROC) Analysis—Top Discriminating Lipids

The advantages of repeatability, sensitivity, and metabolite coverage have made HPLC-MS a frequently used method for metabolomic analyses [32]. In this study, plasma lipid molecules differentially expressed between pregnant state and postpartum state were highlighted, 12 of them having AUC values above 0.800 in the ROC analysis.

**Figure 6:**
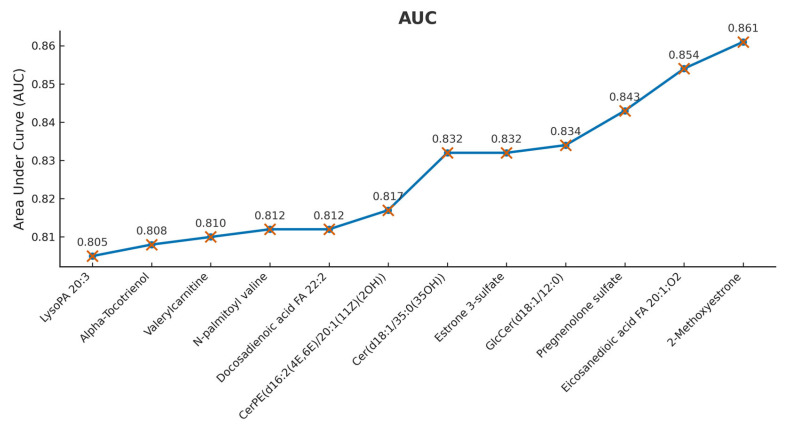
Potential biomarkers in the discrimination of pregnant (G group) and postpartum (L group) highlighted by biomarker analysis. AUC for a number of 12 variables used to construct ROC curves.

Figure 6 presents the AUC (Area Under the Curve) values obtained for the top-performing putative biomarkers identified in the lipidomic study analyzing the transition from pregnancy to postpartum. AUC values above 0.800 indicate excellent sensitivity and specificity.

2-Methoxyestrone recorded the highest AUC (0.861), indicating strong discriminative capability. Eicosanedioic acid (0.854) and Pregnenolone sulfate (0.843) also showed robust performance. In this lipidomic study, ROC (Receiver Operating Characteristic) analysis was a key method for identifying the most discriminative biomarkers during the transition from pregnancy to postpartum. Through this method, researchers aimed to assess the ability of specific lipids to clearly distinguish between these two distinct physiological states.

The ROC curve, and by extension the AUC, provides an objective quantitative framework for evaluating the sensitivity and specificity of a biomarker across all possible decision thresholds. The AUC values above 0.840 confirm that these compounds can effectively distinguish between the two physiological states regardless of cutoff. High AUC values also suggest relevant clinical potential, either for diagnostic purposes or for monitoring physiological recovery after delivery.

However, the method has limitations. AUC is insensitive to class imbalance (e.g., if there are more postpartum samples than pregnancy samples), and ROC curves do not reflect the clinical impact of classification errors. Therefore, for effective clinical translation, validation in independent cohorts is required, as well as the integration of these biomarkers into robust multivariate models.

### 3.7. Integrative Biomarker Panel and Lipid Class Categorization

**Figure 7:**
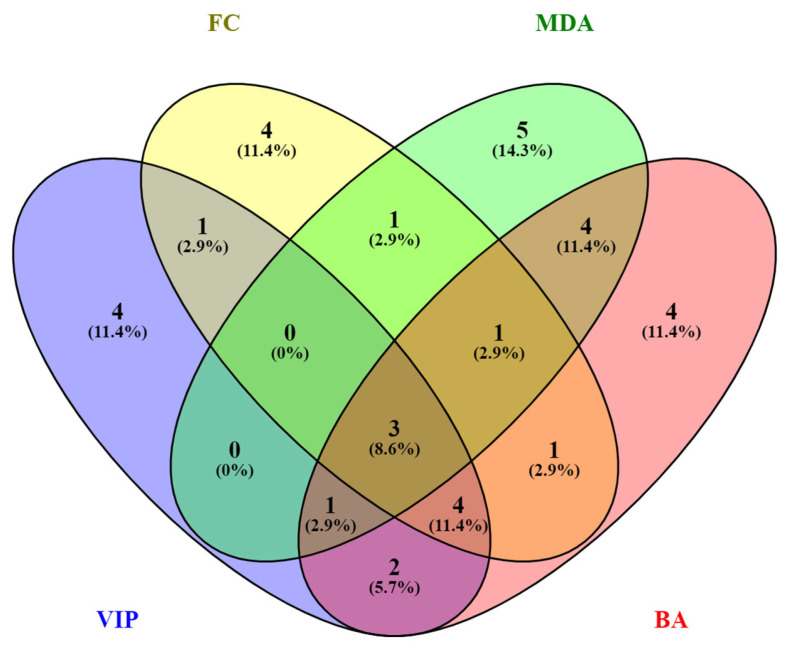
Performance evaluation using Venny diagram for the number of differential metabolites identified by four algorithms (VIP scores-15, MDA-15, Volcano-FC-15 and Biomarker analysis BA-12).

To improve interpretability, a comparative Venny analysis (https://bioinfogp.cnb.csic.es/tools/venny/) (accessed on 4 November 2024) was performed for the specific molecules selected by the four statistical methods (VIP scores-15, MDA-15, Volcano-FC-15 and Biomarker analysis BA-12). Venny diagram showing the common molecules is displayed in Figure 7.

Three molecules-pregnenolone sulfate, 2-methoxyestrone, and docosadienoic acid FA 22:2-were consistently identified across PLS-DA, Random Forest, Volcano Plot, and ROC analysis. Four additional metabolites were identified by three of the four algorithms. These molecules were categorized into five major lipid classes: (1) steroid hormones, (2) long-chain fatty acids, (3) lysophospholipids, (4) ceramides/sphingolipids, and (5) glycerolipids. This classification underscores the involvement of multiple lipid pathways and structural classes in the metabolic distinction between pregnancy and postpartum states.

Steroid Hormones: These lipids, including pregnenolone sulfate and 2-methoxyestrone, are derivatives of cholesterol and play key roles in endocrine regulation during pregnancy. They influence placental development, uterine quiescence, and fetal maturation. Shifts in their levels postpartum reflect the endocrine transition from gestational to lactational states (Pregnenolone sulfate, 2-Methoxyestrone) [33].

Long-Chain Fatty Acids (LCFAs): Molecules such as eicosanedioic acid and docosadienoic acid belong to this category. LCFAs are crucial for membrane biosynthesis, signaling pathways, and energy storage. Their elevated levels in the postpartum group may indicate increased mobilization and oxidation of fat reserves associated with lactation and recovery (Docosadienoic acid, Eicosanedioic acid) [34].

Lysophospholipids: This group includes lysophosphatidylcholines (LysoPCs) and other derivatives involved in membrane remodeling and immune signaling. Elevated in pregnancy, they may reflect increased membrane turnover, vascular remodeling, and inflammatory regulation necessary for placental function (Included biomarkers: LysoPC 15:0, LysoPA 18:4, LysoPC 20:4) [35,36].

Ceramides/Sphingolipids: These lipids are bioactive components that regulate apoptosis, cell proliferation, and immune responses. Higher ceramide levels in pregnancy could support immunotolerance mechanisms and modulate maternal-fetal immune interactions (Included biomarkers: GlcCer (34:2), CerPE (38:7)) [37,38].

Glycerolipids: These include mono-and diacylglycerols that serve as intermediates in lipid metabolism and energy regulation. Changes in this class may reflect metabolic adaptation required for fetal growth in pregnancy and energy redistribution in the postpartum state (Included biomarkers: Palmitoleyl linolenate, MGDG 38:5, MGDG 30:3) [39,40].

Together, the enrichment and differential expression of these lipid classes contribute to the distinct lipidomic signatures that define gestational and postpartum metabolic profiles.

## 4. Discussion

This untargeted lipidomic study provides new insights into the physiological and metabolic transitions occurring between late pregnancy and the postpartum period. Through the integration of multiple multivariate and univariate statistical models, a panel of discriminative lipid biomarkers were identified, many of which offer biologically plausible associations with maternal adaptation processes. A key observation was the differential abundance of steroid-derived molecules. Pregnenolone sulfate, a precursor in the synthesis of all steroid hormones, was found at significantly higher levels during pregnancy. Pregnenolone sulfate is not only a neuroactive metabolite regulating the release of several neurotransmitters but also an essential intermediate in steroidogenesis. In the placenta, pregnenolone sulfate serves as the immediate precursor of progesterone, which is critical for maintaining uterine quiescence and supporting early gestation. Moreover, pregnenolone sulfate is converted without prior hydrolysis into 17-hydroxypregnenolone sulfate, and subsequently, through C17–C20 desmolase activity, into dehydroepiandrosterone sulfate (DHEA-S). DHEA-S represents one of the most abundant fetal steroids and acts as the principal precursor for placental estrogen synthesis, including estradiol, estrone, and estriol [41,42]. Classical studies have also shown that pregnenolone sulfate modulates uterine contractility, suggesting a dual endocrine and paracrine role during pregnancy. The higher concentrations of pregnenolone sulfate in the umbilical artery compared to the umbilical vein, as previously demonstrated [43], indicate active fetal production of this metabolite, which subsequently contributes to the placental pool. These findings are consistent with the results of the present study, in which elevated pregnenolone sulfate levels were observed in the third trimester, confirming its physiological importance in placental progesterone and estrogen biosynthesis and in fetal neuroendocrine regulation.

The discriminant metabolites highlighted in our study, including pregnenolone sulfate, 2-methoxyestrone, and the long-chain fatty acid docosadienoic acid (FA 22:2), fit within the broader framework of pregnancy-related metabolic adaptations. Increased levels of pregnenolone sulfate in late gestation are consistent with its role as a precursor in the biosynthesis of progesterone, estrogens, and corticosteroids, reflecting the increased endocrine demand of pregnancy. Similarly, 2-methoxyestrone has been described as an estrogenic metabolite with vasomodulatory activity, and its relative increase may support vascular adaptations. Chen et al. observed that many lipid species rise during pregnancy and partially return postpartum, with some persisting and potentially influencing cardiometabolic risk [19].

Conversely, 2-Methoxyestrone, a metabolite of estrone, was significantly elevated in postpartum women, indicating a shift in estrogen metabolism as the hormonal axis rebalances after delivery. Alterations in long-chain fatty acids (LCFAs), including docosadienoic acid and eicosanedioic acid, underscore the dynamic processes of lipid mobilization. These LCFAs are key components of membrane lipids and signaling molecules. Their elevated levels in postpartum women may reflect increased lipolysis and fatty acid release as part of lactation-related metabolic adjustments [44].

The lipid classes of lysophospholipids and ceramides also emerged as key contributors to group discrimination. Lysophosphatidylcholines (LysoPCs) and lysophosphatidic acids (LysoPAs) are involved in inflammation, vascular signaling, and cell membrane turnover, all processes relevant to placental remodeling and fetal development [36,37]. Their abundance in the pregnancy group suggests a heightened need for membrane synthesis and restructuring. Ceramides and sphingolipids, particularly those identified as GlcCer and CerPE species, were primarily elevated in pregnant individuals. These lipids regulate apoptosis, immune tolerance, and cell differentiation, mechanisms critical for maternal immune adaptation and placental function. Their decline postpartum indicates a downregulation of these processes as the maternal body transitions out of pregnancy [45].

From a statistical perspective, the convergence of results across multiple analytical algorithms (PLS-DA, Random Forest, Volcano Plot, and ROC analysis) highlights the robustness of these findings. PLS-DA provided strong group separation based on latent component analysis, while Random Forest validated the discriminatory power of individual features through a non-linear classification model. Volcano Plot analysis offered insight into statistically significant fold changes, and ROC curves quantified biomarker performance with high AUC values. Notably, the Venny diagram comparison of biomarkers identified through each statistical method revealed overlap among the top candidates, reinforcing their potential clinical utility.

Molecules like Pregnenolone sulfate, 2-Methoxyestrone, Docosadienoic acid FA 22:2, Eicosanedioic acid FA 20:1; O_2_ consistently appeared across all models, highlighting their reproducibility and reliability. These findings may have relevant clinical implications. Lipid biomarkers specific to pregnancy or postpartum status could aid in the development of diagnostic panels for monitoring maternal health, identifying at-risk pregnancies, or assessing postpartum recovery. Furthermore, these lipidomic patterns could be incorporated into personalized medicine approaches to enhance clinical care and therapeutic strategies. Lipidomic shifts reflect emphasizing coordinated biological reprogramming involving hormone synthesis, membrane dynamics, and immune regulation. By capturing these changes with untargeted high-resolution MS, this study enhances the understanding of maternal metabolic physiology and identifies new opportunities for biomarker development.

This study demonstrates the power of untargeted lipidomic analysis using HPLC-QTOF-ESI^+^-MS to reveal distinct metabolic fingerprints associated with late pregnancy and the postpartum period. Through the application of complementary multivariate statistical algorithms, we identified a robust set of lipid biomarkers-particularly steroid hormones, long-chain fatty acids, lysophospholipids, ceramides, and glycerolipids that clearly differentiate between these two physiological states.

Levels of Docosadienoic acid FA 22:2, Palmitoleyl linolenate, GlcCer(d18:1/12:0), LysoPC (20:4), Cer(d18:1/35:0(35OH), LysoPA 20:3 were identified in the postpartum group, compared to pregnant women in the 3rd trimester. Our findings are validated by the results of other studies that indicated low levels of lipids starting from the 3rd trimester. Accumulation and storage of fatty acids, triglycerides, phospholipids and cholesterol have been reported in the first and second trimesters of pregnancy [14,46,47].

Our study aligns with other metabolomic research that has studied early diagnostic markers of postpartum hemorrhage (PPH) [48], the metabolome of postpartum placental tissue and its potential associations with low birth weight (LBW) and small infants for gestational age (SGA) infants, for understanding long-term effects of low birth weight [49], the effect of labor on plasma metabolites immediately after birth, for better management of postpartum complications [50].

Understanding pregnancy and the postpartum period, from a detection perspective of complex dynamic changes in the lipidomic picture, can bring additional information both for the mother’s well-being, as well as for fetal monitoring and neonatal growth, by identifying some biomarkers for signaling deviations from a healthy pregnancy.

Strengths, Limitations, and Future Directions

This study has several strengths. It employed untargeted HPLC-QTOF-ESI^+^-MS lipidomic profiling combined with multiple complementary statistical approaches (PLS-DA, Random Forest, Volcano plot, and ROC analysis), ensuring robust detection and validation of discriminative lipid biomarkers. The use of strict quality control, external calibration, and reproducible sample processing protocols enhanced data reliability. Moreover, integrating biological interpretation with statistical modeling provided a comprehensive understanding of maternal metabolic adaptations.

Nonetheless, some limitations must be acknowledged. The study was conducted on a moderate cohort size from a single center, which may limit the generalizability outcomes. Future investigations should include larger, multi-center cohorts to validate these findings and ensure their applicability across diverse populations.

Only two physiological states, third-trimester pregnancy and early postpartum, were compared, without longitudinal follow-up across all trimesters or extended postpartum periods. In addition, while lipidomic alterations were identified, mechanistic validation and functional assays were not performed, and external validation cohorts were not available.

Future research should focus on larger, multi-center cohorts with longitudinal sampling to capture the dynamic trajectory of lipid metabolism throughout pregnancy and recovery. Integration with complementary omics platforms (transcriptomics, proteomics, microbiome) may further refine biomarker panels. Clinical translation will require validation of candidate biomarkers in independent populations and exploration of their predictive value for maternal and neonatal outcomes, enabling the development of personalized monitoring and intervention strategies in obstetric care.

## 5. Conclusions

This study demonstrates that untargeted lipidomic profiling using HPLC-QTOF-ESI^+^-MS can clearly differentiate between late pregnancy and early postpartum states. We identified robust lipid biomarkers, including steroid hormones, long-chain fatty acids, lysophospholipids, ceramides, and glycerolipids, which consistently distinguished the two groups across multiple statistical models. These findings highlight the role of lipid remodeling in maternal adaptation and postpartum recovery, with potential clinical applications in monitoring and risk assessment. These results provide preliminary insights with potential relevance for future clinical applications in maternal health. Although exploratory and limited to a single-center cohort, the results provide a foundation for future large, multi-center studies to validate and generalize these biomarkers for clinical use.

## Figures and Tables

**Table 1:** Demographic and clinical characteristics of study participants.

Variable	Pregnant Group (G)	Postpartum Group (L)	*p* Value
Participants	65	42	-
Maternal age (years)	27.9 ± 5	28.9 ± 5.9	0.366
Gestational age at sample (weeks)	34 ± 3.6	-	-
Height (cm)	163.6 ± 2.7	162.9 ± 3.7	0.293
Pre-pregnancy weight (kg)	63.7 ± 5.4	62.5 ± 7.3	0.362
Pre-pregnancy BMI (body mass index) (kg/m^2^)	23.7 ± 1.6	23.5 ± 2.6	0.656
BMI category-Normal (19–25)	74.2%	73.8%	0.960
BMI category-Overweight (25–30)	25.8%	26.2%	0.960
Education-Bachelor’s degree	22.7%	11.9%	0.269
Education-post-secondary studies	18.2%	30.9%	0.269
Education-High school graduate	39.4%	42.9%	0.269
Education-General education	19.7%	14.3%	0.269
Marital status-Single	28.8%	31%	0.810
Marital status-Married	71.2%	69%	0.810
Tobacco use-Yes	33.3%	21.4%	0.182
Tobacco use-No	66.7%	78.6%	0.182
Alcohol use-No	62.1%	73.8%	0.209
Alcohol use-Sometimes	37.9%	26.2%	0.209
Birth way-Natural birth	45.5%	40.5%	0.611
Birth way-Cesarean section	54.5%	59.5%	0.611
Infant sex-Female	53%	52.4%	0.947
Infant sex-Male	47%	47.6%	0.947

## Data Availability

All data are available in the manuscript.

## Supplementary Materials

The following supporting information can be downloaded at: https://www.mdpi.com/article/10.3390/metabo15120798/s1, File S1: Matrix G and L 346 mol.csv; File S2: Matrix G and L 346 mol.pdf; Supplementary Table S3: List of common molecules (n = 277) identified in the plasma of two groups (pregnant and postpartum). The databases used for identification were HMDB and LipidMaps.
