# Supplementary material for: Non-Targeted Plasma Lipidomic Profiling in Late Pregnancy and Early Postpartum Stages: An Observational Comparative Study
Source: Metabolites. 2025 Dec 16;15(12):798. doi: 10.3390/metabo15120798 (PMC12734539; doi:10.3390/metabo15120798)
Supplement: Supplementary file 1 [file metabolites-15-00798-s001.zip › File S2 Matrix G and L 346 mol.pdf]

mz	QC	G1	G2	G3	G4	G5	G6	G7
104.0982	233616	126693	153448	114122	126236	136406	131718	217983
109.0923	18300	15647	15923	14526		16178	17077	15822
111.108	35875	27634	30114	32061	28295	33967	31217	32675
115.1024	2309		9975	10083	10339			10577
116.0728	17479	41301	30200	31657	23836	27615	23073	23217
120.0709	182210	125181	128269	142375	146047	63332	138884	130497
123.0358	38108	32895	34638	39033	36358	39285	35410	40656
125.9763	5289							
129.1175	15853.2	9384			9984		10502	12328
132.0877	135637.9	91264	64833	106818	87993	37216	92970	71636
146.0436	48219.6	29356	27388	49416	36145	20490	33965	39461
149.0686	23031.36	65054	58661	143377	13546	309719	14853	16828
150.0856	63403.56	116977	101435	85226	85571	76686	63754	59305
155.0852	44283.36	31326	31217	30930	29615	31638	32180	32210
158.1433	157202.8	107045	103977	111965	117285	112280	117495	112958
160.0498	600304.3	390916	352733	358031	367354	375589	425160	386155
162.0382	28317.96		19608			24698	20958	
162.1003	97492.56	69118	58231	64291	59780	47082	50978	72409
163.0627	127974	129976	117320	117642	106137	97837	95623	96618
165.0977	19962.36	14808	14913	15184	14949	16825		15776
166.0735	211169.6	117983	110225	321180	140600	66419	131805	118762
167.0844	33518.76	19260	22110	19818			22712	22497
172.1932	58391.52	44029	41970	43135	42341	40402	43029	44684
173.1407	176882.6	111105	109122	116963	118266	117075	121844	123902
174.9915	22354.2	16441	16056	16808	17156	15452	17384	15915
175.1069	36016.2		20154	14281	24243	21077	20453	22080
177.0421	119787.4	67524	69953	72227	76562	71793	81138	83575
185.1014	46621.08	33539	34485	33107	33565	35697	29449	35320
188.0846	1017280	524275	10076	21863	20178	381994	704368	813976
195.1225	94292.88	65300	68293	67675	66393	61963	69327	69148
200.2222	55011	41599	40136	38995	40136	39159	40105	8597
202.2024	31451.64	23929	24023	25290	25539	23759	23875	26476
203.0382	264075.2	464813	425347	337784	324504	370766	286358	232063
205.0771	397424.3	205119	204736	396485	298353	158928	144404	320195
212.2229	0	48507	49431		49411	46496	47792	
213.1329	27185.4	21811	23164	151077	19633	151452	149275	19925
214.2374	527056.2	32640	32199	398042	395818	418884	384966	388654
216.2175	73435.56	53330	50254	53773	53967		54786	54428
217.1366	68654.52	41904	48424	48794	47065	44948	46860	49693
223.1548	30004.92	15499	22620	21286	20444	23539	24678	23128
227.1598	103905.1	370479	68798	122639	381178	69051	67868	69211
228.2531	60874.44	217081	49534	48334	214517	214740	206987	216554
229.1275	62643.24	34001	46641	47252	50147	50300	44748	59667

230.232	1183641	16536	18784	22823	19942	16065	13758	15638
235.154	138417.8	94218	92748	98104	91587	93789	96970	102423
239.1451	525220.1	281128	290648	27431	28320	316173	29873	357593
242.2675	548239.6	36078	41017	426339	408891		422825	418267
243.1195	143757.2	126362	106610	110120	100829	97408	101242	102521
245.0633	161173.3	82519	83867	90482	94788	96840	107020	112578
246.1301	0						73931	
249.1769	594644.2	263966	356331	255481	194056	391538	403345	408384
250.9874	46863.96	36235	36274	34973	37570	36398		37675
252.9843	0	22469	22626	22894	24484	23978	24241	24903
256.2815	1037260							
258.2619	348437.8	355893	373239	285671	335869	278088	334868	252200
261.1231	0	59099	51599	25546				
263.1462	50838.48	39159	33742	31084	36405	38812	33976	
263.2207	10752.72		8440					
265.1144	80150.4	66813	63184	63215	65537	65821	61749	60324
267.2526	19594.08	2480	94962	30643	26912	34614	23661	23755
269.1918	89919.72	122059	151881		116424	99534	77987	91188
271.1779	468165.7	379544	318348	339624	309184	2816396	2842405	91442
275.2224	80073.84	21762	281473	281165	91944	80596	281644	75138
277.2	90913.68	66067	64558	37583	27724	21934	70943	58010
279.1452	138192.1	33457	31634	118741	158716	84842	171953	122025
279.2151	108328.4	100665	102625	106256	93202	90358	90645	91058
281.1623	63761.28	43173	25969	18550	19095	44414	46603	43749
284.3147	56849.76	49938	49582	49148	46441	46953	32565	45242
286.2924	64633.8	619903	58568	42292	35110	35019	617340	39245
287.1453	175975.8	25608	107541	117150	119529	125010	126493	130313
290.2672	27524.64		24279	19639			30596	17474
293.2144	23238.6						34719	36630
295.2183	152339.9	133683	129931	132486	78707	74993	23055	119066
297.2244	117058.9	126979	124773	121446	108053	107253	111313	111298
298.3268	602985.2							
298.3293	154066.4	110240	112012	112753	112076	123900	479205	113949
301.1486	1006408	1400769	1315502	1320352	1007341	798707	882043	897605
304.8507	0	182271	183269		78924	138072		
307.1797	35537.04	34174	28735	27911	26909	26914		26865
309.1904	0				30613	30040		29916
311.2207	47554.32	26123	25461	32835	23398	32526	17852	35446
313.2276	86075.88	92586	868279	89959	86927	137728	96744	86028
315.0558	2319236	1758632	1614590	1646838	1686394	1382798	1768966	1679030
315.193	0	26583	26496	59506		26791	48961	28529
317.187	66693	184667	172935	131478	81340	58108	77304	50749
318.2966	0	110792	107798	109901	93436	103228		97909
319.2634	0	579190	558664	511310	478482		416020	403643

321.2516	0							
325.2515	0	961130	765545	466554	336255	277276	290835	332548
326.9949	86247.48	61402	63268	65003	66737	65147	57950	66089
328.9923	79663.32	62308	64393	62431	64196	65160	65381	64981
331.0373	606034.4	470759	435269	438743	448985	439430	474691	452900
333.0428	39518.16	29948	25538	26809	26137	22402	31330	
335.2192	233339	289124	267609	94578	197999	183539	180523	174028
335.3243	92481.84	71708	76261	77888	72696	72107	73478	
336.2934	116207.5	139067	131862	124894		99497	104168	101099
337.0479	147461.2	118038	10704	83831	95260	61107	102999	78039
337.2131	548825.6	1222969	858029	822914	562454	467948	460615	449755
338.3235	53619.72	143886	28966	96507		63326	60240	58035
340.2364	684050.4	384001	434150	456865	469900	536767	295796	292752
340.3719	107133.8	31210	114679	493911	100369	456453	399608	440637
341.2452	3113939	4542870	4923102	3823751	3625230	3200650	2986245	2864339
342.3573	92250.84	109286	73189		71050		68786	105512
343.2675	155020.8		96700		98556		89336	
347.2954	200340.4	385098	328410		241760	181060		196681
348.2639	313174	246072	246549	244667	239460	226595	231423	229071
348.9766	81539.04	76558	66353	70982	68883	69481	62440	65063
350.9749	0	75244	69968	66173	66023	66593	61145	62420
351.2296	30733.56	92308	99119	66938	57051	38676	34616	32180
353.0106	50330.28	33008	24259	25218			36346	24064
354.3889	667736.5	585327	19228	585327	553055	539304	4484	525706
355.2231	417176.8	395056	363966	361521	327834	307861		315593
355.3568	100836.1	8248	86251	86216	85989	83836	81910	78029
356.3396	42977.88	36258	5968	31991	34741	33257	32033	33485
357.2364	1055209	1754723	1933827	1613204	1346365	1119966	1071587	1036430
359.2666	164870.6	791314	879782	272718	235028	330540	197530	193564
360.3396	544009	541958	490900	480904	458930	445064	444779	424952
363.1979	92529.36			11660			15305	22182
363.2887	523948.9	648842	643921	563283	544864	527591	504089	479208
365.1151	509710.1	761843	711586	679255	544609	462289	445258	425417
368.4019	564999.6	689841	754191	824663	602640	579524	467248	472615
369.2684	1518227	3469092	84854	2117610	1519622	1065750	209284	1541879
369.3512	264022.4	250144		203949	149437	211554	195725	39318
377.3145	161634	26048	266896		213327	191838	179306	129523
380.3145	407568.5				395155	386967	380316	359472
383.176	217731.4	342060	8321	217797	215944	31063	154882	157981
387.2326	48265.8		67983	19556	17076	22194	31246	35921
391.3171	230412.6	377316	217660	32424		249285		227514
395.2218	193158.2	580460	401508			180604		184216
397.0554	146298.2	17851	18226		38866		34517	22981
397.1954	112957.7	1017517	1268453	95856	96646	89781	85536	88305

399.278	392362.1	424036	455030		316819	286381	273894	269099
400.3535	541460	375143	438931	499172	764208	520968	352487	638764
401.2561	1114343	1842293	1907168	1634352	1325449	1130437	1099891	1072466
402.3844	506494.6	408736		419812	424564	410772		407384
405.2407	113584.7	186336	206015	182212	165396	114079	116325	105063
406.0536	53277.84	23313	26984	28062	37488		38921	
406.307	0	1498196	1830632	604869	456752	292201		294960
407.3119	198900.2	229747		201630	194261	204072	191192	178333
408.0499	0	16914		17899	24198			23228
409.2234	0	80878	43380	26985	23086	54343	37331	34067
413.2668	69013.56	1572305	3746002	123140	175384	108912		98864
415.2594	0		567993					19741
419.2596	114218.3	41588	44920	137480	101143	101093		97015
423.2288	60408.48	178875	170525	172961	168228	162606	157265	50682
424.3364	944007.2	1078300	1035704	927101	896990	919056	906731	847960
425.1931	135985.1	170184	381056			182656		
425.2279	124939.3		156745		125694	117329		108378
427.2616	101440.7	82373	77414	26145	79485	76203	76011	75960
432.2662	124460.2				5964	65505		85364
434.2642	0	114663	76685			58260		64177
437.1986	0		625023	677716	476140		375294	359210
437.2519	400579.1	212367	191269		34625	37706		28804
439.2187	402922.1	772612	779500	648771	570976	397743	380415	363481
442.3765	0		293694		157568	115296	87910	108296
443.2381	116777.8	52467	44715	47329	41180	78535	84638	56539
443.3066	153927.8		444806	359782	383180	328914	349600	307427
445.2854	22445.28	1260587	329258	1207368	999955	10663	15036	770990
449.2644	225688.3		408808	409280	272980	230251	224136	218733
452.5016	40190.04	29152			32794	39940		29896
452.6968	433241.2	29120	107205		150478	139984	304112	207850
453.3173	359517.8	157670	595076	535769	558262	76236	929160	598283
456.2225	192052.1	39978	52667	6580	66488	8232	94142	33212
459.2976	87217.68	24716	46136	32452	63872	49193	62109	39597
459.4722	153700.8	116605		128763	137785		160860	165804
460.6957	31830.48		21821		31673	23263		38799
461.2776	133664.5	73522	73424		87281	53384	89299	100544
465.3666	282790.2	248477	256812	253604		230073		
465.7947	21805.08	31679						
468.3558	0	2396412		1608738		43671		1835784
470.2415	130456.9	78271	84178	37780	116374	48639	186607	10976
484.3541	221084.2		258614	274549	257880		169966	196987
487.3129	126656.6	21316	24141	10308	10232	134964	139344	9680
487.8018	44510.4	25403	2196	23054	18902		27202	
489.3091	519397.6	197984	626534	608297			475058	428877

491.2613	515768.9	170238	471895	153488	382785	630039	385514	440682
494.5405	84136.8	78838	104832	85839			71294	55444
496.3739	532740.1	655207	711010	541459	614530	545205	511363	
501.3423	0	4765628	4826205	2532216	2532715	2678292		
507.2995	753973.4		899964		196280	193932	605748	614088
509.814	0	18470				18462		17511
511.3624	175297.3	154055	104826	127508	123146	149726	148045	141699
512.3758	2173072	2235208	2195839	1875091	1908774	1881525		1798427
515.5226	40388.04	31183	33123	32985	33049	31207	35912	37928
517.3327	0	4980523	5007389			129351	4377379	
522.5693	196818.6	153338	166211	165088	175766	166020	174500	154047
524.3359	1002874	6005	4359	613840	459512	479772		
528.2329	415530.7		181777	15662	103907	80553	198684	179213
528.3756	123438.5	155352	146077	152662	146343	134076		112472
531.3522	0			149278	242080	153386		
533.3248	275242.4	299294	293490	294945	339696		313492	235574
537.2974	0	259049	275209	265205	250834	218424	261284	
537.3859	0	56956				39249		
540.4093	1310195	1139310	1112108	60428	903535	716360	916960	1189876
542.28	0	7572	1694944	1419853		658298	1010998	5584
544.1518	0	8124		7161	27188		21514	
545.3619	0	2749041	3165798	2291714				
550.5964	124225.2	90432	92024	104914	97772		105089	106734
551.3231	637655		604930	40532	585868	54092	442336	464431
556.4014	1574316	1619279	1580765	1331949	1333533	1288064		1248122
560.1602	0	11348					14212	
561.3546	3246510	3017296	3137138	2552039	2595125	2602131	2588120	2569594
562.21	17010.84	27840		20412	24868	26396		25056
566.2869	504435.4	562482		69924	388624	280136	410318	462156
568.2966	0		19671	131114				207864
571.5822	2106.72	10974	16640		15413		15715	11584
572.3939	64091.28	72226	68321	72485	74737		62636	
575.3746	46854.72		89938	91537	92657	89494	84487	87388
577.3524	0	61752		133975		131784		122511
581.3191	0	147364	153079	144006		144823	180604	135079
583.226	0	8424		5804				4936
584.4274	1143513	19822	1100962	1010232	828723	674836	874499	903102
585.2151	0	8569			6284			
589.3836	2372757	2191206	2171965	50768	1524345	1196552	1724079	2327040
595.3426	248065	247711	250013	229068	238161		207413	34264
597.4329	0		35123	48371	51305		760	33920
600.4213	1049723	1003081	940542	1115836	853375	849921	838062	838136
605.3796	2001488	5281	15246	9602	1542453	1569551	1573307	1566304
605.8028	39706.92		8948	11059	13754	16294	37438	16628

606.6721	10707.84	8621	9242	3112	7358	9297	8940	
607.1078	8511.36	6881	7401	6826	6625	7142	7557	6885
609.2967	0	112943						142832
610.5348	0	139924	151944	145107	154927	143054	132636	126942
611.3233	33493.68	32865	40640	30694	30824		27884	27988
614.4439	0	26987	25621	26885	27293		27294	32787
616.4176	32650.2	33020	29041	33829	37162	35320	32185	30585
617.4279	0	41674	40063					
619.4279	0	8666		13951		7655	9286	7537
621.3719	320089.4	350580	359016	287688	72042	72493	70909	64142
623.0868	54981.96	38748	1956	38647	39719	39063	40200	38949
625.3417	130981	93489	14292	90564	98877	92636	94407	93244
625.4468	0	55284	25752	29812			25836	21460
628.4495	784571	793014	797130	720659	645044	573102	601536	618915
629.0961	27009.84	23949	20610	21498	20727	13297	22445	20864
631.3101	0	41945		7464	5795	6344		9504
633.2217	14761.56		23880			12953	16504	
633.4051	0	1328207	27733		1104460	974099		1139111
634.2582	14405.16		12572	14111	16396	11968	10888	13952
634.4049	460867.4	62948	52747	408618		57079		53695
636.5553	60355.68	42856		53952		42558	42201	780
638.5607	376821.7	342022	352641	339692	365482	313514	299875	303596
639.3641	116268.2	110472	105030	105619	107627	95820	94159	3707
641.4382	67851.96	58301	11886	33915	33535	45144	32952	30090
643.5182	29879.52	8801	22300				23968	19710
644.4431	675066.5	598950	569583	571481	524136	512407	514045	505436
645.0653	17854.32	16576	13632	14477	14839	3962	5236	13719
645.5167	14308.8	5792	6757				6916	8307
649.3946	1191172	958322	942707	882761	852807	876515	918750	936505
650.2559	0	3521			13036			11712
651.5169	394875.4	415734	428334	374769	382600	253647	364332	259119
655.3393	15953.52	14868	16888	16784		15181	13096	
655.6663	15323.88		11782	13600	15830	18919	17621	18682
658.4611	20739.84	13713	14257	15832		15229		
660.4391	16480.2	14907	12398	14016	18655	18094	16589	16060
661.0296	12789.48	7872	7841	8115	8992	8489		8757
663.4148	43695.96	36932	37704	55370	44431	49595	47020	38959
665.3939	44754.6	34183		195912	188504	39223	188632	47464
666.5878	164829.7	143036	136037	149481	147851	134639		136296
669.3663	74148.36	57774	61059	61541	65009	62249	60478	62221
672.4711	457732.4	459149	443117	408631	384263	377459	362592	364338
675.3404	3908.52	19035	5232	18370	13257	7076		3384
675.491	82047.24	17555	74558	73832	78012	48466	61929	65745
677.4284	842302.6	774471	774686	736633	637672	641371	656919	657770

679.4567	89831.28	58940		63896	60213	56614	62204	57668
683.3948	10586.4	7865	8496	7474	9460	36508		7625
685.379	37254.36	46274	49352	75982	23337	16560		27058
687.674	0	50094	48925	55816			54335	54354
688.4722	397052	320731	295164	1588	288377	298147	1644	287700
689.6877	0	20483				2540		3588
693.4191	201400.3	528696	524282	527092	498363	526515	550613	548411
694.3539	0	7893		8241	8155	5820		6973
696.3574	11782.32	9874	9816	8824	8688	8312	10014	9266
697.4779	21057.96		20348	21712	22365	1884	16909	17198
697.7011	129364	48741	95065		98202	100048	142472	81688
699.38	43728.96	32053	31006	33280	30478	29014	32631	33164
699.5809	25865.4	7549	16121	17183	18987	19578	18731	19903
701.4499	282216	269668	229439	236540	275733	268734	247938	236999
703.5173	156834.5			132668	136780	148470	132614	226584
707.4405	0	12596	12995	13464	13773	12730	13177	
709.4068	0	99389	14411	90289	87911	88837	89977	102776
713.391	46860	9940	36559	38169	41051	39598	37821	38930
713.4949	17476.8	17700		15968	14724	15288	13544	
716.4925	0	223971	227584	204309	207232	202125	190405	198195
717.4072	43449.12	34390	28944	22296	36441	26934	40133	19628
721.4475	614346.5	10328	572781	530399	379254	344661	376246	445453
723.4839	14530.56			13187	15814	7332	7617	9891
725.4988	58825.8			50181	45647	50476	47449	77477
727.404	0		11131	12984	12562		11780	12229
729.5143	28963.44	13319		19784		27990	23896	16709
730.482	37583.04		32168	31813	38550	15252		24296
732.4944	150880	171505	153629	423242	180502	214238	106448	114682
737.4851	31793.52		9941	19413	20832	24603	10172	24000
742.5059	46522.08			13732	28706	33746	37209	25051
744.5179	36267			27902	38760	51413	35521	13422
752.4681	12014.64		8527	11594	12976	17445	16065	6818
753.4267	75377.28	58010	7717	53610	50904	51590	52532	51588
754.4749	87052.68		16773	275851	104552	123144	61849	70520
756.4931	25011.36		5906	38766	31198	26828	13517	8985
757.4086	33770.88	17160	6137	20327	22219		22009	
758.5085	1805380	12792	8552	1798061	1206554	1288395	1368811	9083
760.5253	16008.96	5000	12182	98061	589449	13659	478513	547906
765.4722	0	200348	7337	187149	193520	216076	218704	
766.5104	19162.44			10874	7742	17427	13461	11351
768.5206	54599.16					9616		28556
776.5121	107371.4	91073	78567	81791	80925	79804	70579	75761
780.4898	1348669	12888	26236	1256729	799578	897205	1046216	900858
782.5064	156620.6			743708	829920	901692	678057	593348

784.522	313344.2	3721	3521	110740	337474	171947	148547	226712
786.5326	569004.5			3398	185336	335989	384356	358300
788.542	45010.68			4495			36032	48825
790.51	0			17499	22692		16392	17916
794.5335	65031.12						36464	38514
796.4637	85658.76			81885	50539	55201	61120	56954
797.444	33634.92	32224	30332	3759			29608	
798.4773	38724.84				488	33322	30830	32672
801.4326	14698.2	8169		9684	11109	10174	11284	10734
804.5005	62593.08	57240	5571	951251	21078	845289	32412	790634
804.9875	0			47076	37369	34220	34084	
806.5042	272067.8		20977	392923	252579	72461	66989	241199
808.5184	49280.88	2456		38760	41201	53781	39678	57227
810.5288	0							
816.5513	94603.08			103431	66974	69762	75569	71035
818.5559	40933.2	2656			34101	36268	28124	33974
820.5259	56072.28	40998	33138	41220	42181	40269	35897	38506
825.4906	98482.56	73011	66562	73622	78184	77212	77428	76080
828.4859	13935.24		18509	190934	130549	123359	8895	102955
830.5006	47490.96		6860	65987	45152	56954	25526	59233
832.5163	18601.44		4244	8673	18836	8330	14609	139019
834.5313	65770.32				46758			
840.5496	73263.96	2643		34390	46489	58041	44985	30761
841.4642	17905.8	16830	15458	17873	15705	15578	15157	16340
848.4812	14168.88			3868	6487	8123		8832
848.5609	28819.56	19296	18910	15957	19352	20894	20623	20558
853.5174	52547.88	40630		41095	40560	37424	37419	
858.3987	0	5204	4702	5110	4559	5196	4970	4772
864.5569	8981.28	17951	12891	9516	10113	10989	4830	5796
869.5092	44990.88	33689	29276	32827		35003	35227	37105
885.483	9859.08	7454	7016	10144			7523	8417
892.5842	9522.48	7925		6566		7344	7206	9212
897.5385	25945.92	16955	16705	17342	18252		17786	19248
908.5772	0	7536		7130		7539	6771	7069
913.5321	18919.56	13435	12185	14939	15746	15596	18988	15615
929.5067	5157.24	3338		2282	5120	6368	3258	
941.5601	9221.52	6923	8720		7493	8936	8636	6860
957.5535	8943	7356	5476	3773	6778	6546		6123
991.5927	0	66332	40001	27127		17860	35924	43438

G8	G9	G10	G11	G12	G13	G14	G15	G16
191006	174806	243616	204882	105450	137585	165202	208996	206240
8779	17773	17900	18478	17984	17385	18042	18756	18954
35029	33200	33775	33409	35152	32555	32811	33241	30526
10276	10973		12286	11681	11044	10935	12059	12484
7649	24018	17479	20087	20101	14207			
142079	114877	171910	130823	168177	115475	112506	139146	142482
39844	39805	36108	36597	36733		35542	36045	40198
11294		12010	12024	12274	13463	12687	12230	
103709	82861	102756	101268	116524	132504	59758	112929	103548
36544	37337	36530	34366	44493	32813	29143		43818
16288	15794	17448	17548	18311	19234	18044	20475	19115
20436	50469	48033	47147		37757	40313	39597	39385
31519	32014	33548	33946	34460	32496	32848	35657	33196
7854	126538	119093	126707	121980	120294	126252	117551	120827
389180	388516	454776	429758	487569	454002	444062	469723	470783
8276	24058	21453	24581	24020	25435	23773	26194	
45167	85710	73858	58780	106032	60699	32149	52624	87319
95573	96706	96950	96370	96902	90974	94218	102263	100970
15267	14911	15123	14553	19491	13928	19347	13667	15422
9075	108964	159977	111641	144383	118983	107310	140038	133801
9371		25393	25247			23996	26221	
44191	45556	44236	46199	46524	45245	48996	48313	48245
9406	130797	134002	136932	142088	127764	122736	140635	143856
17100	18436	16935	18386	17344	16857	17152		16030
7342	19702	27285	24775			32715		
81169	85934	90748	92729	94450	92481	89834	95296	113117
34459	36453	35319	36936	36101	33849	34467	36058	33694
828308	802344	770667	748193	958768	29317	22471	16562	32853
68256	69465	71434	67586	69602	293917	100721		48253
41455	40286	41675	40015	38203	9242	40004	9538	36997
26179	27575	23827	23489		22916	22018	25114	25245
216814		200057		266555		357165		330746
324160	129517	301079	136475	126435	359840	270828	317959	143582
48570	48276		47025	45880	43673	44180	43134	44777
19848	158279	20595	156940	161584	150660	22879	22732	24658
15049	395746	399285	390427	404291	355087	383697	370361	400618
55699	56496	55633	59844	60893	57152	56381	57895	61254
	54264	52011	46989	55097	201476	134044	100123	88743
21658	21704	22731	21936	23329	24330	22367		16265
42627	74442	78716	74746	75258	72818	79605	76708	79380
213358	47500	46117	212262	45745	48573	49745	197641	195584
16056	54796	47457	45760	55746	48491	47564	51625	60209

12932	16156	896698	904997	16363	828116	16611	16334	12991
102377	106681	104862	105646	103905	105174	105943	105635	110842
31867	398293	397894	414498	436182	103951	430472	435110	461766
9550	410737	415333	420175	409326	395450	404823	399887	396358
108542	116756	108907	121951	121572	120418	138165	146295	133272
115073	124360	122101	131553	137292	129899	137377	144422	165115
429542	433352	450488	468360	467358	420668	454935	484772	497644
37921	37369	35503	36761	37567	38258		37254	38211
25004	24606		23923	24043	25281	23667	24272	23484
		785803	763836	775724	720846	746357		725858
2962		263968	336692	238615	12801		303868	212934
45393			47941		85947	5148098	37341	70280
38220	35932	38514	35516			32898	35702	32332
		8146		7798	9868	10479		16297
62755	60325	60720	46826	46636	45850	55419	44618	53843
19986	19653	14844	19678	18081	16241	21173		16627
75645	70253	68121		65183	53993	50546	59442	70693
347530	350221	354671	380542	395173	386871	403750	435348	61738
85568	66805	60662	49514	297421	288018	68008	282488	67695
21163	19742	68874	21092	71046	18029	21126	19370	69770
122519	160765	104691	84471	76956	77754	88164	88637	86651
91190	93424	82067	85229	88815	89712	97281	88224	90828
44097	45470	48304		47798	50294	49223	47220	49956
43939	44549	43068	41355	44926	41349	51563	46209	41961
36336	27866	48965	627630	48500	35870	64931	599722	29773
128088	133150	133315	135146	16916	118479		129298	133451
	32412	20852	31600	16304	17397	22437	19393	
35822		17605	37728	38890	15740		19813	25991
22298	119673	115409	61653	111548	58966	123074	122891	75509
111615	112063	88681	91118	110321	109544	110573	104256	97117
		456807	462443	461117		469048		455104
114107	476687	116717			454728		447842	123318
820634	766584	762430	853622	759612	740046	892801	994061	969632
198568			197216		153633	191692	159807	153605
14574	27293	26922		30078	28692	30025	29564	
29402			29410	31379	58274		20914	
17382	14694	36026	15399	37766	31986	35334	30514	35321
88606	100423	65209	70331	81117	78212	74028	80986	78363
1681891	1640510	1756997	1777088	1827594	1385325	1477839	1679424	1726371
65730	29902		30910	30041	49586		47311	35803
55824	47283	50525	53291	48965	51726		61677	74906
	127612		93500	99276	91714	97626	96878	
392180	378392		365823	352880	335269			351311

								11821
311503	215326		230026	176184	201032	256212		207710
65637	67896	65339	64233	62435	64778	61554		
64343	65476	60351	66258	63537	63872	60392	62794	61710
5672	428800	459117	459775	474724	433827	450772	474134	413005
27404		29938	28061	28949	23675		29817	
87441	114751	176772	191918	193314	185846	98265	115784	109231
	74844	70062		69955	68152			
99078	100706	88036	98671	92808	90743	99234		97166
92695	75846	111713	96055	101864	25515	102135	93995	105599
25883	431594	415777	430324	426867	404185	478572	521562	546038
57447	39054	40621	43680		37588	46424		
312175	473579	518220	164843	641836	33863	362253	690856	432672
81316	83314	81162	367923	93739	405243	84453	426405	92455
104956	2636985	2359045	2581256	2561889	2497107	2752902		2684574
	70515	69887	73852	105752	71540		101585	102857
106612		117440	115300	120988		125412		140796
188041	141202	151773	158540	122731	151309	177133		146367
6786	241761	237253	232436	231926	217240	228895	233554	229157
65317	62409	61772	59678	59232	55281	56920	59556	56568
61011	61427		60725	54483	53534	54882	10912	54424
61087	24653	23283			24433	26476	52182	
20901		38129	19171		50995		37935	
510177	526753	505861	515897	500217	503584	512495	510053	520137
321701	324688	316043	2396	326212	333328	349729	347346	477532
80624	83183	76391	78434	74678	71576		70974	
31314	31165	32559	32457		28879	29280	29363	28420
955200	891578	799401	848753	823511	812353	862413	873665	996468
170751	161706	124902	165693	148895	121127	170513	400619	128685
435161	427178	412128	418482	424449	405451	407136	421135	410717
28764		70098				28549	39664	20891
480589		396931	441699	452083	424243			394289
424366	409723	386144	396532	404025	367370	402034	420246	
464909	429729	428030	406288	496979	404015	432955	442271	477461
1441175	222011	1150172	1251887	1008015	1185584	1554903	206264	1418924
245308	231676	200017	233972	187042	175588	235596	240700	179701
138549	131718	122450	133625	142716	115210	116094		90062
368126	356815	308764	343027	355443	335114			326391
	141529	164948	12077	158964		7500		
35180	45620	36565	49961	45258	43133	38575	58461	60417
12787	206624	174555		174165	176889			154229
161192	16280	146332		14985	121460	167644	182656	179480
33922	15645	110832	55415		29369	35638	18789	26229
13795	82905	85574	83304	84911	75710	79336	75611	78406

276305		297244		258185	248650	271978	115140	265725
421358	382412	410197	370846	446404	553816	474215	662304	371998
247676	45916	844199	49723	839396	843265	56174	877768	50157
418476		383708	382684			365384	385484	
97531	92649	86049	86590	82313	85242	91475	96941	109457
40076	39553	40362	41658	42220	39373	40333		37006
262453	141604		185702	151879	159220	215828		156896
176399		150682	159518	164416	155131	159762		145554
	27772		29564	30832	2476	24604	30960	25588
27394	41622		33713				21714	33066
2763500	94616	52283	82947	29971	91484	92044		46007
504933								
77731		86529		87801	74002	88800	65304	78072
52123	138894	45764	31767	49868	139699	138008	70433	133431
865838	836166	715157	797015	844799	750850	798251		750898
141666		103019			6860	143900	139128	197964
103319	105002	94651	97967		92890	89987		9872
25424	74449	76849	73615	76731	70203	65713	75525	76214
90384	26247	94288	24669		74881	83286		12134
72460			77429	83012	82174	82990	86692	
						309215	351410	
21441	348409	303469	313205	364944			25271	211924
351633	33218	305244	310441	303373	276031	323259	349702	341512
119844	76460			61395	68192	80903		
87393	87680	88468	90472	38744	79983		41857	79848
324046	333528	116612	314888	301634	301038	319615		347620
726114	15174	17004	288672	662851	637535	254820		17364
244440	191618	170976	188366	162151	176496	188548	200513	199384
28749	31538	30447			30220	29227	40100	30205
225617	182360	328213	174714	22910		193190	30480	
28278	70626	272362	307705	75027	240287	626943	90915	690249
51679	25620	145494	91641		91592	46709	58629	76243
64378	67822	66074	62070	63550	56392	66554	68822	38463
171060	163952	116440	145212		159484	155100	158480	139262
49605	89814	24114	73670					
95132		101261	99186	99195	88412		95368	77659
243360	213976	214235	209235	196445	190697			
		16519		31121		26446	30616	
						1595924		1559456
98996	243057	98831	7676	61388	226760	82835	9184	159756
185082		167488		155585	153949			
20513	119992	95952	10470			91424	124944	
29700	26686	33720		36820		29664	22863	
	8600	393483	470540		381891	345708	500720	

54590	353717	390734	338410	188546	323622	370429	316728	344883
69769	69018	63740	66025	65360	63362	63825	52142	65909
469628	631620	403591	444143	378013	535703	508035		371879
	129400				150044	3162352		
		571192		590624				
	24441		26417				28752	27688
137343	133857	132801	136178	132231	120199	122769	111581	105806
1817118		1646267		1851248		1919088	1473346	1842592
	29898	30597	29654	29925	9868	35004	28182	27947
189812				4450960				
142924	142932	149105	154070	140680	132566	171464	128631	138308
594759	576828	759753			522708			665248
207378	151410	314796		38370		206587	28871	
1340	106514	93514	102888		93802	94078		116600
			197659					
236806		208517			215646	229246	289580	
	230916		191160	224252	181375	195120	211876	232558
	38812			32170			91664	
74936	844462	992572	979572		956368	890186		693217
930002	688159		829469	704756	816225		1076765	
6877			6348		15565	45444		21375
				2449172	2610841	2513357		
123048	96442	94110	90639	83912	86172	89692	91138	85924
449743		483072	39908	124168	427321			
5635	1225885	1192664		1409508		1212975		1146735
8222	10128		7980	10324		8687		
2436994	2464508	2459477	2568167	2381967		2741900	2399879	2616970
20236	11828	12887	17899	18088	14963	12812	24416	
295915	380484	382148		329052	283677			
107633	109916					22400	266037	193937
16719	14749	1596	19208			11024		
5517		48554	47548	51454		57988	49897	53322
94908	82646	35496	87089	87256	88127	94250		89940
61960	116519		48524	29596		120621		135426
133321	128411		158204	125250	121458	137024	144095	155390
9844					24108		9816	
808445	1073248	866298	836358		821625	801242		769919
					12723	7548		
1621976	44772	1797543	1794479	1781127	1855793	1939496		1828833
203000	201854	187928	205007		59924	200584	193484	58752
35663	39540		28387	31432	34792		32653	29622
834321	803954	795245	816958	782408	782930	801368	737096	753960
1473725	1440739	1516279	1553832	1507102	1561095	1628510	1517415	1580172
10799	11713	30081	22874					

		8112	10284		11140	2262	8068	9612
7037	7091	6448	7788	8372	8914	7263	6503	
135300						113510	111259	
131241	120602		145024	97330		114732	107969	107655
27995	22528	25374		22485	21659	25432		
30185	28510		27101	27488	26039	22035	25864	25825
2232	27677	24735	24576	26754	25615	23729		36424
	12356				13883			
9323	51180		7797	8815	19495		53412	11655
52016	61014	242492	62885	58474	36373	32912	24272	55308
37718	37237	41653	40410	42991	44079	38403	40116	37102
91451	84213	99228	91130	87422	85982	93084	100928	104403
22821	20596					16090		
590685	604048	594372	595916	594186	563114	574260		556123
19900	18851	20462	20840	21190	17034	15306	18193	19815
6115	6568		6988	6264	2712	7652		
		11183	8016	19140	13981	8372		
1117444	1139477		1147916	1193018		1185893		1201014
2941	12504	10913	1690		1712			11623
47074		349142	45970		36928	43695	42707	47201
3300	37947	45724		2076	35653	33974	34861	2264
290683	260595	285471	282533	261525	247344	266274	318096	256657
3108	91604	88082	92579	87997	95233	93096	92247	13428
3464	53373	51403	53732	23231	49043	47287	46890	25467
17276	19153	22636			20484	24448	14568	16162
489830	501874	511414	524092	472728	496221	524414	475908	484872
13684	12982	13526	13517	12933	13842		12786	11235
7050	6719	10840		8096	8127	10036		
849520	872324	902403	916253	854585	936723	988540	909916	17051
13604			14536				14048	2300
2540	239073	299148	217421	215990		272124	197190	312376
	12351	12086	13268				11709	9173
	13677	11609		15152			13382	
16989	17456	15712		16069	15511	16683	14431	15046
14872	13573	12485	13743	13233		12818	13380	14311
8601	8436	9689	8897	8578	8020	7523	7887	7219
26632	32933	33103	35670	33867	26773	22189		32028
34852	147384	33905	35493	33777	141564	34501	36964	142024
115736	118922	124871	120685	100205	114186	120068	105185	120877
61921	56991	56173	62854	59958	61035	64120	66406	70372
366792	356373	346767	359534	344036	353146	349288		331520
7960		2961						
56346	69665	62157	73839	48124	39708	45051		52125
3745	636430	638108	652250	666787	685352	668290		686851

56931	58120	68054	72335	55350	54363	63010	55693	52614
8487	8436	8020	8175	9021	8885	8431	8070	8211
33231	24874	28223	29135	26131	29919			33425
51455			54806	50065	54206	56030		55585
294463	278764	300797	296435	278158	1236	1240	275966	264637
			20934	19706	18648			3104
520160	493189	152576	539377	522339	540809	544088	534594	543598
7704	7205		7406		8757	7230	7949	7246
9096	9725	8926	11592		10489	10552	9641	9778
2404	18475	15953	18474	12374	10395	12285		13587
	109527	98003	1960	141904	120612	120572	90579	94557
33468	34782	33128	1420	33725	35635	32098	32810	
20905	20913	19595	18675	24024	25396	20695	21364	19453
233132	230309	213800	212846	211829	207562	215392	210267	200529
126354	132409	118814	144279	92818	104399	127014		153141
11890	11868		11502		14463	14093	11588	14190
67832	90976		20312		68743	61503	20545	73339
2413	34715	35500	37559	39284	38304	39506	40596	41788
16364	12885	13240	13675	11929	13218	11971		
198592	201242		199843	191423	191155	195110		178166
18970		32916	28635	22197	22977	4252	22319	
491135	457249	465414	470898	479605	506658	525291		458474
7416	8332	11008	7223	4566	11929	10211	8236	
44694	53459	44565	53095	37787	40316	62356		59464
10626			8887	12392	5863		11018	12537
18330	19752	21942		14131	19887			16224
27049	31829	28472	43134	15775	10766	22796		19058
66087	84838	114303	87718	191784	72404	39353		142568
8307	25283	24086	25002	23677	22568	22011	8991	22211
27447	42088	35244	34578	39217	24012	26974		22078
23194	45105	27475	51595	27802	26672	21600		18183
8084		9102	11021	15999		5360	15387	4834
50334	42315	57104	44373	51492	38907	38436		41274
45695	51181	65949	56284	5280	41674	32921		95483
	16490	18948		18124	15711			22173
28756	21501	25584	22773				24107	
1241677	1365757	1367712	1185139	1464239	1402359	1068821	21637	1483399
14217	15110	12128	14575	15652	19336	23901	22775	21988
218320	187944			233116	232396	247396		244180
12418	17605	14517	17657	27122	19056	19288	21232	22190
32419	56647	41363	23328	47187	37859	41128		9799
78211	69443	81342	85103	82449	76563	79806	80902	76009
877236	1046762	1021719	828793	1078531	963995	950176	11088	1126563
130616	755102	118652	740062	813455	709616	633878	76365	881486

252447	207511	237382	231555	175955	393771	185020	171038	203418
302399	327650	431064	371916	414789	391822	474265	423990	296216
37239	22984	34099	24718	26120	27028	26299		19548
11480			10759	16364	12464	20363		13925
35189	7238	49266	54792	46761	46883	43103		37787
58839	66625	64893	56885	71267	65222	59540		73893
	22821	25481	30256		21722		20486	
35884	30907	29337	33997	31936	31920	35408		27937
10523	11433	11135	11641	11510	10926			11711
771998	40872	47419	34301	794152	27583	17417	33115	828196
30274	31719			28372	29901	28801		26790
98585	317526	206112	81763	52096	51960	52427	70257	243914
60636	45552	37334	61779	50960	42303	47623	171780	38398
69729	80839	71669	69629	82186	75252	64557		88444
34655	29934	31010	32317	29370	32086	31809		27309
39630	34976	42479	42701	41874	42465	41909	39853	37971
76113	65491	74608	78223	73314	73799	76839	74731	75142
124172	111657	10557	12871	101978	80169	67605	113574	98728
54562	37193	35978	52295	36776	29876	35230		17810
142352	12222	14092	14808	177399	20130	160204		11350
48521		49826	54259	42662	45263	59966		
50590	55356	55503	53661	64897	55084	48862		75418
16414	3178	13565		12607	12597		15472	
10111	11999	10734	11236	10989	11875	12488		10547
1958	21802	21833	21239	21725	20929	22912		21007
39682	37582	39809	40545	37901	40426	42882		40455
4627	4805		4699	5832	5087		4640	
13458	12929	6804	5839	10344	7516	9976	15766	
34564	31152	34084	33856	32461	35324	34770		34492
	9760	7469		6425	5375	6547	6156	7872
9025		7214	8008	9580	9166	10747		9030
17696	14775	19656	21540	14255	17074	17700		19136
7447			8771	7676	8420	8015	8697	8573
4673	15239	14333	14448	15678	15291	16328	17956	15336
4186	4380	3907		3912			3304	
5832	5960	6986	6456	6384	6747	6723		6519
6343	5801	6775	5625		6911	6555		6082
49776				24023	31946	34596		47980

G17	G18	G19	G20	G21	G22	G23	G24	G25
157555	267450	306727	210009	187216	212589			211223
	14782	17420	17534			29100	33443	
35235	32443	29460	31918	52719	55204	57363	55593	56916
12244	12868	12645		15406	15855	14870	15488	13526
15774	17385	11206		34495	20754			
110767	137707	153878	209540	1847412	2225806			2499821
		34947	38041	55139				44469
14853		18386	16088	38841	41535	37673	38367	41902
13582	13527		12607	16446		16510	17896	16567
42358	98583	89165	159833	19886	22956			21239
	39086	46173	77653	194720	17423	18777	21094	23027
16879	16733	303584	21680	23740	23748	30275	35883	25498
34306	27794	32519		22991				
17170	28619	32648	32899	18449	19149	43704	48915	20960
87162	110743	123795	127553	100578	89489	141528	153411	94585
380119	410596	465074	488433	562747	677639			670635
25023			27572	29458	48431	52067	51978	44136
42616		23133	90276	337151	130061			328277
94607	98044	105752	106695	141794	143954	152122	157500	156754
10419	18963	12449	14910	17392	16484	35553	23287	17180
145043	178384	156431	195131	1839937	2254826			2539072
		24782	27253	29439		33561	28325	
46594	46121	44295	48314	54236	54499	61787	69603	64543
97040	119254	11753	136256	160681	149436	176683	190512	141436
	14536	96334	16885	22730	22493	24622	25429	21386
				57875	34102			38415
83463	92073	96659	126377	95071	94330	135138	152181	102543
26662	33862	32073	32411	42688	41898	44032	46243	32569
1052163	894226	1022077	1720806		4960080			311451
63369	85587	140576	127581	47727		100922	112781	32416
27190	32759	34705	38418	35816	34669	48193	48890	37218
21481	21052	22354	22519	22456	23720	29263	33157	25460
276286		219711	353363	563925	508592			522268
127172	130977	435606	163178	1767380	204147	182493	198510	2590228
	39787	40448	42833	50326	47158	53315	56328	49871
92404	136028	149906	24225	30031	48037	53371	51710	56513
242096	284100	327929	386080	348849	318571	432217	472477	332240
59179	60766	58721	64708	70134	69783	90232	98196	77130
433839	524389	389811	298878	68599			86034	
21077					37317	29192	37391	
303884	313027	73846	77560	343060	353768	506622	122851	360031
105066	42964	41079	46413	83841	59555	181774	67031	79463
46735	47287	50856	55020	57404	64803	48311	46702	70613

635294	15118	15045	16885	796320	20165	22409	19047	23994
96857	106220	109320	113392	132907	137148	165200	163048	143505
112475	470236	403111	465738	597836	657993	55006	731359	764445
362733	364066	375227	377790	455266	26956	27221	501327	28017
136520	140594	158759	169847	288940	319840	385461	398462	351103
105097	120837	139483	173757	47010	148362	246621	279962	152779
				75870	184031	299766	300553	157149
81271	87181	433962	466883	521385	476135	597360	642886	472240
29433	31934		34249	47882	47014	55640	63927	46981
18446	19679	21867	23070	28817			40309	29943
647956	671308	664738	661597					
193625	15937	228582	253377	234838	328691	248870	258804	255834
160494	187408	165488	3920460					
	35185	30341		44795	45198			
		19262	21243				19692	12538
	30233	45064	46958	124364	158216			54008
	14116	31643	31115		22393			27738
48516	37666	47705	135983	137043	88677	129509	132469	150876
11996	407790	453044	14833	762454	296619	4096057	4460403	833126
56080	58569	66922	70308	141266	29772	93295	119225	159073
65928		50952	62716	95535	38137	38429	78305	47290
76403	81346	84017	101422	162756	142127	125284	139214	158537
89439	89116	91873	101175	97579	122627	258950	277474	116204
28170	42077	51169	51979	65442	50169	48972	39653	57741
39788	42710	39026	39954	43752	45123		41971	42536
28615	492704	46152	29483	552844	540895	715939	25306	549535
80959	91484	112069	121489	105497	111313	137507	155450	111919
	13424	17478	21439	34152			31162	29829
				122871	95136	73691	59133	
67677	21277	106732	126260	34460	154696	179658	172231	145584
108288	83672	106362	113777	107450	115473	225999	141732	122903
410056	411883	413168	424192			543171		
	118346	114901		220994	248765	237263	237932	527202
771911	858999	938224	1277355	74656	53219	1742507	1936855	52115
123172	160732	137202				212260		
26124	25329	26132	21505	21004		48543	56019	45118
100115	72626	20281	35221		50729	54223	54172	50521
	30654	15329	17372		31261	38510	42630	32098
	95576	338574	69044	136856	67016	133384	139311	123442
1420039	1181712	1593198	1288598	2751529	2208537			2570248
26331		29633				43450		
	53576	64105	89945	134974	141853	108527	147760	181438
80153	86828							
	282302	298629	327737	512951	511859	608966	682163	584207

	9809	24213	4452		42958			
	240766	289169	376933	671550		124442	118311	622474
	55534	56581	57912	70819	69880	93561	95954	70930
	53982	57647	59511	70496	70865	83398	90961	
435274	402614	447373	45856	607126	557427			621691
25366	20040	29198	21778	43821				42458
78140	91552	214260	17068	373935	405162	493912	511087	497491
66057							84098	77054
83498	6416	82604	92727	156398	149446	181628	203731	170830
138319	131794	100127	82714	21768	104476			107501
434000	468270	527857	647312	1523086	1564895	1632552	1813856	1807990
	40784	44911	41043	69349	50024	18230	29384	56927
686452	689517	465934	440853	239552	262124	1142882	959290	254769
377037	81704	82361	336960	600270	614881	612341	592405	114255
2259154	2251954	124253	2763210	4487187	4563781			5460534
	91271		95904	12878	95615		106990	
132492	133124	113415	129392	9691	100920			
128362	150684	189199	177088	341223		68517	79129	284833
71865	168213	212310	223062	75971		306927	14853	52340
32993	41792	44905	50648	74845	72972	98671	107373	71746
31966	39479	44198	44660	70331	66237	98854	106920	75232
		28357	39215	212005	28664	27642	271019	113500
51029	39671		34889	42383	63808			63519
454396	485693	485189	495581	732281	760066	789048	764636	930032
330153	351362	342477	378161	376655	426050	465114	525206	481921
60078	62391	5704	2484	44849		54002	56318	
11757	25267	27662	32324			26143	26020	9945
930220	113008	873472	1130149	1789628	1627728	1724965	1765543	2147079
32419	104155	491129	238937	316253	179106	913543	146210	191289
374365	396493	388680	384912			619267	579895	582908
		12710	30910		221611			52362
351773	343331	339564	365375	509949	524078	723868	729329	558899
348303	354996	403867	451835		702254	728751	779253	757152
341086	384690	402887	499306	587589	679826	626379	604239	672697
1202204	1347902	1591060	1785032	3801542	394572	530191	647274	2850768
38346	34017	157986	153577				205996	
	93306	102860	109392		140829	138140	173067	
	278535	278835					601881	
	138791	142847	214640	247296		346836	365392	382768
55753	54454		53725	65462		79817	57002	
162093	164971		164815	361841	216019	208154	295208	319406
135700	134652				435264	318929	414659	541252
49760		107212	100360	278602	236153	39286		180271
61911	64356	66918	76306		66057	78077	80219	

278848	279652	271381	357064	58568	186912	248748		555492
	561536	293432	346828		289058	246242	421340	436764
815687	774442	841930	1156289	1783098	81541	1759839	1680322	2186609
355764	376852	354848			10352	523224	531556	286317
82990	85472	95501	127632	153881	167169	150521	161107	202286
39421	38929		33775		77628	69685		60814
138378	180113	309379	237790	506092	157572	41980	113935	
129192	129596	28775	130071		141447	197055	65328	156714
26753		23982			48740		41511	
	41256	45875	29962	50103	39199			111929
2578784	76348	87692		765140		469688	495482	173844
407041	556292			432698		463803	438400	446675
	60065	21004	100236			170657	174071	
80774	119632	124246	47780	162517	164650	151125	153552	161394
670444	680110	699936	702227	863110	799206	1026015	1019330	934333
	110568	125100	214348	214577	267136	270916	171704	
169355	108829	7160	88032				10136	
67800	65180	68093	72439	43144	79465			41549
3288137	3126023	573698		200740	193392	164742	169752	30592
80507		85001	100024	161648	158736	178088	164447	155156
2996412		344036	50580	601956	518812	526420	528066	554619
	27116		33045	55699		62181	80189	
305056	283154	311730	363415	587614	559088	533572	586499	588623
54450	64815	91078		138464	52356			103696
42730	77786	74139	69584	44280	46586	54452		
272822	279086	318356	128464	417171	367560	333994	311465	464313
642057	206216		947994	1228636		1204708	1171099	1529437
178639	205042	195090	253440	69192	333020	298904	317259	80608
27435	30021	2980	19613		36770			35535
	79514	16704		361801	64133			181339
463376	425402	244902	90016	126204	1664619	128785	123614	618633
	70581		101228	42768				11035
39702	62990	64329	33643	46912	33308		52717	53542
	128970	4520		144440		135036	112527	
	52409	7509		79216	20810	3060		
79205	69198		65801		48128	43236	53944	
	168212			15068	4956	117336	120949	
36708	29595	25435	18009	25521	52429	55801	3908	63060
				1264426	1174458	1311504	1231971	
84941			256080	12952	62076	47076		89984
	116172	155299	171208			198637		251420
129652		10322	13944	265053	220574	270507	286202	408810
	30330	30779			48052	42657		53414
	386742	423879	261088			636248		764464

49413	58848	284941	279934		88317	242771	62525	115848
61299	56336	58277	66875	77305	82792	69528	68820	70683
811308	487764	443554	457166	889033		980638	1005981	1408
	171612	4634500	2491920	5118103	3497894		5668904	5984745
414713				692812	680628	670457	684524	
26418	34012				29115	34820	37462	
122569	85534	67818	102027	85211	86083	109350	25889	81564
			1496514	1420690	1355848	1351081	1289313	1286577
36174	31037	27941	28793			6560	5868	35462
	226016		200732	4605944	4607209	4803816	4945456	4950682
134710	2280	102780	132256	141369	129393	120799	115896	159780
417111	685712	1087930	994560	805644	467224		8136	268158
				836975	128690			47428
		92488	115608		2456	114135		
		211596			160138	197203	212144	
	269016	4658	288709	323923	344512	329463		376502
209872	226212	211810	263220	258398	294792	276551	279035	310927
				62872	48720	48004	40829	
1748	64120	1044464		1037315	699733	1015862	1008320	1068109
		1037472	7280	1002696	983688		17305	9752
31523	41824	6187		4735217	152664	11700		4179392
	2584219			17400	2088272	3146701	3307864	3973176
88989	83132	82555	100304	65472	71723	59833	55554	55628
	316428		508404			599766	600232	
1110839	1105503		1205168	999014	990782	894461	880030	916626
		10660		20715	26468	8199	13100	12108
72528		2622225	2637456	2971403	2917984	2826090	2878974	3070117
	7322	25284	20776			24126	23240	
320160	600692	443469		395944	366820			836509
	1188	429087	16640	18922	152490	19122	21024	73456
		11749	11713	1708	15205	15920	12018	16087
	48263	49774		57828	60067	60460	57111	58621
83304	85906		122828	105593		124001	130497	114269
285184	94392	132648	149720			161509	166101	173968
142558	133127	148472	172105	170655	179178	177447	177305	189423
	4440			39792	37509			26557
807124	767496	744101	739521	732363	497389	721849	664432	775267
105707	11034	7560		26095	5547	7536		6665
1964064	1864596	1957178	1986519	2049888	1425476	1968657	1939398	2483541
203595	77496	194138	219413	281668	248885		270036	306716
25654	23310	31090	27421			21849	19615	29856
740750	772122	733959	720216	646143	636994	569467	553727	592760
1591376	1620244	1616877	1602597	1707731	195044	1632771	1650464	1760783
	15140	12685						

	7055	9188		8136	9228	8100		11724
	6952	7884		9695		7784		11050
130256	97640	102284	129672		7525			5956
106700	94324	102107		115577	101421	95035	89318	98768
				35020				
25152	25349		26851	20331	21788	22620	18765	21569
28283	1348	26622		29088				27654
	9604	13864			13821			22220
52687	8919	56750	9564	30536	19035	10948	11474	16883
68293	64307	68826	72239	214252	81175	76096	77826	75704
42570	42435	38023	40580	42408	49455			50774
91906		104446	107487	107455	112891	112893	111465	113929
22936			13088	18448	17217	18375	17741	
527815	12436	514780	516157	507055	280878	443152	399939	487983
21940	15772	17140		27276	22294			22248
	5495	6891		29174		11471		20061
42520	12038	14587	28425	44183	82622		18528	54182
1168312		1228836	1241880	1304955	716808	1159399	1065431	1480220
	11707	14200						17468
45185	348688	44279	44367		42075	43042		
	2993	32823	44868	42200	34065	27468	27926	31203
240819	219222	231853	259477	193339	202982	172935	155561	182911
	89517	88055	104412	134434	119735	121109	123959	141866
23393	7751	22572	39976	20452	21484	39761	21908	67839
22536	18626	17696	12169		19212	22761	19309	
459946	487689	443393	455442	403107	405401	358753	344609	366529
14548	12686	11207	11626	15186	11887			13894
8816	7035		7312		8040		11468	10452
946682	928758	925346	950256	972725	991219	913385	898753	992102
12872				11176	10916	11355		10896
303704	284244	211185	312564	414308	427936	394399		410410
4381	4669	11381			13094	15418	14421	15224
14935		12012		10236		8252	5666	
15311	15586	17369		13706		10558	10708	
	12490	13687				14296	16680	13515
8049	7611	7492	7120	6634	6998			7608
28489	32091	35548	43059	45096	46052	52219	39766	40864
35202	130652	36110	136504	38476	41525	116498	39237	38969
111439	107270	109532	110685	92060	86216	71512	67421	71691
64176	64094	69254	69909	67161	69048	67120	69234	71621
	318881	321685	319113	322006	149604	264382	228551	286476
1576				3500		1440		12493
44638	14251	44641	62513	172127	43377	26010	5234	
649505	671404	704258	736185	804211	341539	633799	547808	833675

47383	60787	53919	49587	101714	100942	64453	58829	65165
41044	9747	36047	8602	48849	44752	12314	11328	
31536	33399	35492	27016	12784		68942	42962	43088
	52796	52027	54955	46754		55647	49288	
262959	281323	256821	1220	223973		217895	1136	211310
	20255	19761	21604	3280	21087		18792	
544715	555786	537189	554968	516970	558135	525195	508697	537016
11164	10402	8546		16624	13545	11515	10134	67040
11809	12795	10998	9824	13563	14155	14894	13947	
11129		12105	17078	137484	28673	17686		
102368	97676	82965	88972	83084	89155	112644	90688	130576
20211	37787	32929	34604			39335	40061	10205
17907	19913	15916			21695	22385		22468
195356	197175	188064	155288	224486	211678	96467	132402	215669
153444	181650	62418	192969	9440	89421		57213	
	13819		14606			13025	12296	12376
20470	61991	20861	19861	63053	57322	65685	61386	76000
40447	36752		40810	12124	38286	40067	38328	39540
	10999	9448	11974	12652		10760		11621
180919	172587	172796	179444	180450	11525	137912	120541	156133
33665	25049	19690	19480	31522	23582			26230
468378	457804	493552	460807	445081	216350	333021	299191	407267
20649		4959	9535	116900	62796	50510		1468
46068	71204	53803	162288	38895	18673	18123	19361	1032
11738				16329	14168	12321	13418	
	7489	9800		9858	31423	14154	13813	10550
12724		9266	16714	40997	11483			
87960	37612	20117	98476	18718	83818	25741	23103	120420
22699	22335	18792	18645	9622	302846	274522	200920	20841
28521	17796	14590		6737		16184		
22841	22010	14211	9365	8014	15711	11565		
				29752	11183			
52664		32630	46068	34869	39680	36828	10161	42318
	31626	22930	98029	16472	100473		16172	3821
32400	25694	26059		54276	37850			16455
		24209	22996	20387	27480	20711	21300	19811
1346712	1217221	363502	640419	576301	1007077	708720	809976	
26574	34759	32563	20530	9441	65568	262805	163611	286359
235596	207672	222152	205526	213953	147473		151278	
26712	14201	23290			29601	11770	10127	
45247	15478	36909	6501	11117	8816	39139	13386	13268
76847	81854	72011	75351	62678	65533	57159	56267	62611
695865	856525	822118	1093678	348743	446078	383304	450388	13500
748969	624171	205183	591402	286428	524313	200722	203782	8679

442971	200314	184651	48289	97467	358512	123483	104151	10261
359524	507070	448854	299770	266114	16787	222891	108844	96835
14614	20673	44728	25949	21810				13449
22582	16633	14062				33985	13339	13347
34696	37078	38387		11936		32217	13124	12621
50329	59626	51674	75198			22425	24055	
25865	19322					6572	5376	25576
25048	30414	39478	21160	11342				17737
		12371	10575	10853	9157	9271	9400	9220
40052	731287	6826	904102	50824	192847	231849	215630	38182
22371		17373	37393	10740	9801		8758	
147158	69972	75318	213685	56259	284489	261309	102164	
47803	47659	26379	43674	18724		198107	124074	306555
	214039	257335	50598					
72600	74652	32731	45045	44760	81012	54793	64580	
34144	31169	25717	13962	17064		5208		20877
38011	40874	34554	36754	37144	35160	26249	26018	30446
78825	77792	74543	77724	69029	80452	56534	57229	70825
96586	66883	24148	110373	76277	170607	102665	133115	
8119	35638	18613	16412	18703	22770	8672	12967	
29105	16262	192629	31838	28193	14066	87711	10593	5562
32332		45838	43813	57435		20068		5857
58457	50069	13518	40914	24714	37403	25885	23400	
13293		11008	12993					15360
14025	12172	14256	7500		4159		6569	4572
21118	20942	20384			17951	12411	12894	
45360	39489	40574	40474	49928	42547	33224	34629	42135
8682		5062	4745	7811	8024	4609	4701	9735
10648		11320	7663	15208	30622	8828	9133	14232
35200	35527	33567	32756	35380	27680	25064	25094	
	5451	6518	8660		3740		4539	6470
10329	9660		8579	6129	7817	6936	3615	7494
21228	17377	17968	18620	17522		13552	14167	17716
8396	9394	7752	7030		4834	5320	4473	6812
	16555	13975	14735	12697	12652	10428	10512	11807
3067	2276	3213						
7075	7089	6747	7050	6575	7984		6054	
6661		5998	7041		5341			
42092			59628	62132				

G26	G27	G28	G29	G30	G31	G32	G33	G34
			150688	96934	183572	297007	235239	139889
20745	21115	21837	19287	18171	20019	19128	19282	19623
32994	31297	32748	30374	32939	28667	32124	32035	29503
14718	12077	13831	12399	14182	11473	12403	12795	13516
			12682	13767	14131		11605	
			193208	187515	274951	132911	175434	310687
			33982			39817	34754	38744
18398	24057	20091	22914	19309	23594			
14208	15207	12726	14182	13368	13959	12050	13921	
			64966	99252	27905	79243	45046	48288
			63097	49788	76168	36545	57180	
22110	23763	24080	19458	21851	354846	18577	21887	23130
			27204	30051	26808	25391	27670	30053
35248	36972	37072	34136	34751	33444	33290	34559	32987
122106	120548	123444	128260	118920	118711	114400	131125	139586
			611835	646568	508096	468212	425690	444403
28500	28575	30424	30481	26016	29070	32240	29428	29394
			95541	147803	126934	63338	74697	125861
114529	111293	115475	118130	115107	109786	99617	101475	93850
14715	25727	25883	14560	16311	22343	14041	18592	12681
			177204	177365	232109	122215	163530	309578
10668	10379	26975	24902	10622	25917	22753	24481	
47385	50851	51381	45857	48021	47468	44399	45876	47766
162006	157425	163775	144579	11778	142535	139749	144926	153132
17959	16817	16194	16583	122460	119495	14802	115763	127828
			13819		32617			
120859	116851	115162	93166	98002	97786	96536	98675	110476
36115	37102	35489	33875	32749	35052	33555	33790	32468
			1501787	14836	92862	42120	1275346	
84360	72928						70942	
9908	36361	9758	36288	33664	34719	9382	36472	38086
23591		23047	21015	20704	22761	20109	20107	22958
			151579	161019				
178053	177166	178449	178094	173326	157369	334164	517127	653544
41865	42167	40620	40860	39051	41883	41207	40359	40264
171640	31406	28432	30254	149178	29603	26305	163729	163131
369739	31792	24954	28863	26899	338648	352464	345278	357775
69885	68607	66267	56395	55919	56232	55368	58100	64891
110898	96109	73134	71866	60467	55942	53629		
29634	14213		24943		25918	28571	24212	26477
71564	74109	291620	80473	80581	80828	80959	82563	79144
186009	44848	43691	172099	165201	44000	48620	44554	178385
	49505	57067		41695	37286	58060	55883	

26534	27697	28235	23446	20310	17383	17427	16213	712191
	120508	123466	116677	117559	114297	107378	116384	115587
539332	520236	42751	518125	520635	514025	505984	464092	437087
369892	31952	29133	30583	30341		382492	381184	394051
234815	240242	236462	220370	221568	221821	196972	200685	184906
203530	209258	215060	161816	165787	162360	168324	168825	204415
				3257472				
586859	591678	586517	514321	496867	495816	512942	521121	539649
36200	37683	36516		35204	35579		34273	35094
	25205	20930	22544	23696		23570	21265	21047
671342	649540	654241	653896	656208	649627	655151	650584	649683
212200	15073	238325	16313	282916		313663	274291	224244
77650	89971	50881	52767	63002	43339		36792	
38275	40189	42382	38095	37136	30832	30551	31198	28358
			52969	59327	24590			11612
				47481	45983			31701
			81463	63147	63936	68606		123781
167782	169350	173843	93079	109090	87210	81015	82092	
514320	569677	560049	566286	3421612	554505	511089	496593	486111
124106	123652	129159	86848	90019	67888	72208	64688	41311
85098	38711	40988	33686	37232	36077	67363	65831	22183
115283	115834	116114	170783	112572	295274	88237	207544	78964
108470	105857	104882	103242	102683	103533	24921	85279	87748
59849	59537	53379	53319	54008	54580	51594	50396	
36690	34042	35725	40851	40117	39256	39088	37632	
19455	21969	19279	40485	43648	28740	567211	15811	117108
150428	151527	23814	22747	25771	117427	122780	132117	134328
21539	20899	23322			20972	16722		26024
39068	39875		42339	48389	32085	50548	51776	52821
128518	125006	39223	127376	120565	59848	74687	67687	27198
139675	132359	131485	122299	117058	120590	108688	111162	391717
410827	422286	406311	419810	403220	410972	429599	418155	422751
117981	121557	114934	118297		119216	123369	124741	116400
1442626	7848	1498065	1502400	1481981	1351988	1150475	1054684	1002239
147842	23600			163448				
31451	32785	31666	30633					
20334		20298		33701				30966
30888	40121	42498	24886	29364	30971	32496	16596	10210
80314	104743	79640	93673	736485	258836	71700	194498	98601
			1307486	1474690	1208234	1384083	1660667	1410758
37100		60956	33369	32701	60709		34763	35850
182069	186359	190425	164269	181596	157881	131652	96630	16296
92925	88762	88305		110332			86052	
604349	570825	594162	305927	318490	268043	266250	246076	141600

36474	41879	40608	14704	59127		19311	41769	
891915	920235		753908	756356	305569	370100	231835	116141
60593	60792	55964	58327	58104	56652	52240	54445	54633
61887	60355	58097	56168	58176	56303	55704	55604	54220
			449660	459188		49532	425995	
			24847	27753		26287	29021	23162
295146	298117	301380	138376	287817	265960	127188	102899	201836
65944	63327	63045	57000	69497		65136		
164935	158603	165417		4764	78954	78818		
			112809	100181	78368	86432		35024
30224	29438	909303	913378	870531	728204	602687	545619	483912
99410	94994	98949	64831	56518	37466	40130	36299	13501
805540	810482	673420	213582	738233	703744	509533	656406	723097
79751	323873	77054	375009	95510	347308	371205	82360	178361
5948040	115082	6047455	3094216	3496581	2453274	2624140	2333701	1474072
88694		83024	9180	66210	86673	6176	61145	92687
124400	118136	120384	130468	135384	135128	140328	142576	136392
322933	316058	312043	244560	219431		151918	124139	47853
262641	261073	264626	222482	212070	10839	228721	235737	9729
51901	55008	50185	49705	47954	49136	45779	46085	43178
48552	52831	52517	44817		48206	45627	45587	42953
107375	106193	113089		88199				
		18098	6576	68248		31202		
433650	457594	456766	24300	472870	468537	449530	467858	487499
440427	441895	435986	419634	425421	416639	517472		350159
59977	63170		64647		57949		63762	59624
23921		23080	6668		25826	23315		
1935589	1981352	1969443	1709624	1735072	1562441	1362787	1311287	1204055
348901	361211	313957	721428	557119	561392	606483	592782	1171819
278015	371573	359589	387371	368124	385042	370981	318617	365126
				25638		44411	60346	
618412	604184	592200	285832	337972	303851	283553	288649	255773
543502	540553	535415	533653	534977	518746		442944	
524181	520311	504537	7264	559140	507111	523482	490278	1025860
4267836	4266979	4245789	3705276	3408080	1378131	1694158	1516100	525548
	117108	140612		30957	57775	62186	196868	74282
179035	203917	198838	124362	128341	94346	100363		53319
503730	496594	494363						
148889	167088	143983		139001	129307		168964	167476
57798	57423	24332	34517	35004	34215	36146	54184	60310
329288	315333	366820	57201	156279	168940	162308	35509	
286369	276335	278692	303552	325580	273120	202812		163188
					103744	17241	2244	53014
78633	78639	79530	75989	76442	76433	78752		73076

444234	455914	459557	190744	382168	279215	372260	278060	166828
	249216		262162	426788		442448	288440	
1844229	1818023	1826350	195668	1783968	1605974	1463362	1337764	1199412
235367		324952	264844	305176	320404	324096	350248	318388
158353	160092	162310	168168	167314	149997	138748		118439
33787	33896	35057		41416	39288	40024	39261	
1001745	990644	990859	909152	959025	339954	340460	357737	106245
135237	142051	137876		114780	101593	99244	101303	104156
26328				22963	24416	25554	27045	26382
106048	81544	78272	46525			30237		
159080	165848	154448		815715	858996	758190		882088
	380262	373932		364818				334240
184378	184864	196164	88254	90473			74942	
42532	44294	108611	112019	42994	110569	22694	39608	106813
892083	916823	884547	587581	638541	629800	583543	615136	593320
271148	163025	266928					282016	
93063	94298	97630	93462				90521	
69819	35110	30886	65446	67436	29005	25497	62922	189024
22203	25224		24493	22136		121668		91370
96640	88107	88193	94744	92572	103504	96999	88656	
		372616	415136	421328			339384	
88380	86863	88423	81205			23142		80384
372500	367841	102212		386727	121728	359653	321416	314871
303135	287293	282684	155444	183154	68682		70092	
42770	35694	38344	60498	36560	38269		76909	
444203	461930		158824	397176	158504		306376	154828
1228865	1229914	1246210	209302			27424		
305691	358848	298057	89169	31681		260834	261335	
23742		21914	24920		34552	23623	27777	23925
			221961	188429	175943	239299	227818	204337
481806	500309	66410	737940	205799	83872	177825	168848	100322
	20973		54124	375821	50855	70667	12528	90746
34623	29203	29541	44900	31451	39334	45250	26700	33481
	93830	117672	5954	104655	109878	131204		
			58407	105899	190844	33783		67212
56818	59077		59968	60592	73676	59591	57802	
129699	125737	128128			141964	136727	130848	
43234	44611	48828	71472	46072	47524		46977	
1392172	1396800	1381776	1295716	1293496				
27092				10376	105187	184632	80356	7844
188455	175816	171249	178646					
356978	361923	357147	174772	12860		15272		145260
36051		34413	48916	54684		40290		
	624126	195199	622264		727744	683396		

288819	232422	284842	195634	440107	190974	272454	263501	203982
52193	51245		57886	56295	62704	61818	56867	63242
901672	875254	858347		364895		782512		
6122935	6092444	6035777	4324248	4256570	150396	122208	1090540	326584
852816	753494	874480	143968	59588				
26313			37088	33658	33202	36303		
59970	64288	63877	86531	73318	94489	91210	121195	63341
1425601	1412876	1351900	48648	1453180		1522352	1282918	
5757		7196	25106	24231	25074	22786		
4884765		4735798		34242				
	108212	98823	119914	115604	115150	124701	97666	132628
			1489204	1343652	126052	1128261		1884472
			396547	397116	331513	173355	648541	97052
	96591	96115		98415	108836		90556	
225960	226265		169300	223807				
73152			6861	301484			294746	
264226	263212	262742		265517	262080	303528	253047	229169
	44552	43112				37324		
963777	922289	880974	732347	714487		841976		
	5391	28364						
10447	8816		15206			19487		9756
3499139	3435009	3444394	2827344	2859169		2631313		
61071	54587	59679	65123	77996	72578	98824	87703	107004
653276			521998		556512	219788		67708
1536	959415	918958	96400	945468	927955	1079968	910147	1047076
	11515					11014		
2985915	2923306	2901916	2780623	2987049	3268312	3202036	3307556	2685302
	26532	21575	23168				24036	25996
			1199976	1450852	4092	57508	71160	
	17138		189024	15004		507451	410509	
13024	11006	10287	15780	11436			13928	10108
51668		45898	48183		50366	60240	50963	
122912	122322	120737	89895	100296	90036	84857		75947
132453	29188	52256		74264	149694	70412		
151553	148178	150439	158514		154624	163859	151237	149134
		4752	11988	10288	4908			
674993	644130	589431	579347	567197	614372	637097	781800	583088
7528			16156	6092	6228		6412	
2114137	2108314	2001866	2007107	2051151	2334304	1977685	1949416	1775664
274611	271846	297968	245691	250888	238614	231848	224147	240395
17317	17056	23160			27456	24836		
625137	611367	592238	622617	501813	601681	594323	671924	644272
1661546	1691368	1671914	1632801	1749160	2001344	1660546	1671546	1642502
			42092	41168	38069	13740	11318	

9748	10448	7105	2756	9904	4900			
			8259	8841	7643	6400	6915	7104
102464	85276	98244	99492	97977	121396	124168	133628	129668
71127	67261	65468	70385	85870	116264	104643	70263	
	32268	28483	27444	29253			27379	30996
18574	17424	16837	17972				21432	20623
		21204	24280	23847		32800	25758	
	7903		42163	72653	33245		28316	
8664	58823	8239	57940	15051	56245	59692	10852	10474
63975	59290	62148	64463				68990	32084
			43432	43564	42955	35579	36436	36597
93406	93209	88977	92000	93460	98526	103677	103171	104544
17664	16812			16968			13581	
414533	409274	373171	398095	399503	421799	434697	438734	424854
					8621	13976	15647	13166
22967	21537	18608	18127	17244		15189	12849	17104
			20462	10402	1176	9256		13881
1197803	1215734	1168207	1177301		1419004	1184575	1373816	1068657
11203		8548	17017	14184		9446	12628	
	35592	34298	38835	33338		39355	40721	34538
28484	23256	20720	34120	27093	38172	2460	40548	2620
180121	163246	160207	249223	229644		260057	296848	258150
114928	112763	108740	116944	111390	129580	117315	114685	129320
27406	36167	38384	39272	23013	18934	27980	34957	33270
	19924		13432	19608		17158	15626	
384340	363329	351146	362583	314708	366744	357405	350378	327220
			12767	12772			9879	7705
6459	8212	6866			8384		6628	
929512	884089	882763	906397	1012767	953352	963560	980614	958209
		12224			10360	3224	9703	12960
551153	629700	570515	386140	324634	310217	248148	279261	431888
14562	16460	2604	17752	14732	15239	14227	14029	13869
7904	7912		6310	5600				
			11439	9368	10872	11462		
9042		9407	10365	9990		10764		
			6869	7919		6513	7027	
27593	27051	25514	28110	30827	25956	4019	39971	31552
139556	26284	31649	145252			36908		38432
79888	67923	77967	85870	87223	89926	103194	103327	107665
56837	54965	56037	59167	57862	62592	65745	66327	66426
241225	240641	221628	242523	235522	265744	268502	269628	250400
5180	14790	3924	5748		12528		10200	4356
				7218	13989		8935	30075
653330	660831	648450	672700	650234	658698	677612	673344	590430

	46954	44024	51213	49885	55442	51786	54545	47015
37172	37206	9279	8867	9374	8269	45217	44451	9131
119866	127223	122899	1564	167882	137212	176416	141255	253767
	50420	46684	49107	40271	52439		52323	
211303	1712	198664	205345	176210	210930	205603	198216	195385
20096			19848	19129	2748		20068	2644
477148	477287	477560	535187	578916	19140	572123	556945	577800
6858	6020	6757	7780	8282		8263		
8027	7428		10302	10440	716	9653	9290	8303
1708								
92810	113348	92790	120252	87945	92992	94380	72194	100148
37337	34365	36055	33549	34025	36919		30395	36654
16668	17090		15848	17424			22708	22144
169081	166736	117541	206251	191028	149583	146154	138369	145816
					113047	89487	108806	227124
10999	11641	11473	13406	13355		14575	12313	
14805	15118	14162	18208	14826	18284		129668	110939
33751	33444	28955	5693	29926	36072	40557	37663	41177
10708		11704	11368	8429	8806			11510
123716	130369	111634	140691	134101	174692	162162	153641	140804
21723	17053	19994	36478	17832	29185	18251		
303788	318166	321551	336164	339420	358775	345205	329544	231848
					37366	23851	30177	58389
					62625	52645	31637	78511
				11738		17316	15708	
9017		9505	13634	15171	5313			
				9156	20233			30498
111443	101739	103685	113685	103427	23514	8706	7311	95827
		82456	356308	106080	19862	19297	18417	19357
					5453	6575		
					8933	1724		6158
			8656		14126	16591		
	7580	6818	46638	67594	45851		11906	73784
					48006	19297	28077	240258
1116					16230			27903
17316	5502	17215	16998	15568	20641			
			4732	4006	807266	198896	112119	1389890
	49733	50498	74268	74211	82265	83992	213289	102735
152627	158638	154697	180647		184188		183547	
						7247	5897	213888
						17074		
	49264	47826	59103	61010		62227	61265	76424
					43342	67122	44928	125470
					496469	361996	548805	956838

					32799	108210	46296	53754
							72764	47911
10375						6450		
						43513	29705	13118
						20960	16413	36503
					25704	6880	8866	55977
24445	23019	24101	3406	4061	29669	34660	29937	
						9935		
28336	23371		37538	42252	470814	32888	41616	501275
					32937	23823	32511	34404
				10217	183657	40975	94628	298753
					39222	26721	39811	64841
11854						41338	37732	105545
					7135	8423	6836	10879
				2300		5995	3929	
24507	21667	20551	27704	30266	26808	37892	29586	39976
	50506	50133	73844	75608	78176	77226	74960	70748
					136221	49725	58599	130935
					36974	26836	34265	37984
					5493	119399	97530	126790
						21941	16666	7244
					32887	22823	37507	65839
14021	11267	17384	12904	17270	19748	18732	14820	15126
						12698	11693	11266
9802		6856	17059	4011	18356	18725	18052	15587
27852	27981	27369	40967	38971	37223	40395	38820	30492
3944	3909	4143	4819	6105	5157	4292		4492
9923	8151	7538	14132	13401	14135	15751	15535	18236
26341	21670	22105	32375	32693	35410	36893	34979	30549
7258	5860	7099	7520	8399		8030		
2857			7783	7199	7578	8722	7596	5112
10616	11122	10992	18000	18397	17587	17531		12385
3511		2764	5332	5652			8156	6156
10952	9537	9295	13148	15812		15706	14926	13593
3008	3027	3495		3595		3151	3149	
4237	4270	5356	7199	7771	8320		7101	5452
4828		4193	5741	5312	5834	7292		5226
			101403	79732	67642	96523	87369	

G35	G36	G37	G38	G39	G40	G41	G42	G43
157589	142943	152151		132069	134896			
18942	19698	18609	21658	18171	18092	19432	20725	20094
28208	28757	27484	23032	29057	23093	24342	25379	22677
	12217		12254	12142	11531	12015		11395
	12148				12583			
205298	247378	171856		188914	230681			
31991	40983							
	23584	20468	21585	23965	22595	25223	24929	24736
		11391	12595	11550	10712			12606
163408	156991	117304		95204	128436			
	71666	51674						
19639	19301	18979	23539	302689	21813	22197	22260	20918
	30581	28111		24894	23530			
32571	32393	34359	36989	33968	32459	36562	36398	37090
122323	116791	128933	114393	126204	122821	115550	112900	112633
490249	505937	530157		536981	482278			
28957	19179	30212	30038	31801	23819	29079		30203
168071	205058	90479		108465	192464			
92411	80517	85980	93655	94100	95775	89882	90560	95454
12578	14054	19542	14760	12148	21532	24272	23457	22671
205583	231324	160910		188155	228363			
	24535	24600	27077	23458		9928		22792
43994	43118	42418	48966	43704	41515	44880	48753	46222
141436	144238	146450	149537	142083	142333	156660	154521	155770
15874	15100	116671	15868	127364	111543	15098	15291	14802
		23558			30131			
97782	100334	95558	113773	98131	97140	110611	118118	114222
32328	33895		35732	30334	32950	34050	33778	33554
33818	70510	22400		1585977	1270319			
		70433	78841		72059	76490	76886	78775
34459	8896	7474	36018	30682	32479	33673	33785	33254
20743	20757	21410	21444	21519	20020	19462	21070	20318
	234216	172211		183272				
121606	101478	94159	118366	630392	131360	123798	129667	126732
		37466	39916	37373	37823	38442		
156990	153689	159947	25845	26638	155086	27072	171192	173669
324697	323547	333376	344789	328886	323850	336187	330448	341003
57503	57510	55752	64010	55092	53135	63617	65274	60352
53061		52848	57119	53037	54508	60263	54596	55581
23341		22031	25509	23643	24793		22995	21244
78399	79838	97215	90363	77960	79331	77283	70958	75233
39187	162849	38918	177516	39396	38857	39528	171045	171839
49025		38010	56781	45116	47838	46064	47366	57659

708426	695808	712065	698118	659197	15629	13252	700663	14767
111487	106400	111938	116638	109799	112678	118000	113174	117286
	419592	406882	418214	399669	353806	39249	36937	38329
340184	335227	339394	337968	338121		343151	343746	28600
182602	157320	153849	206682	200098	199859	230020	223812	223728
173099	173119	167672	215750	168855	166714	216905	220822	226427
	210845	195189		211244	191301			
519747	502370	500815	579570	506255	522128	583378	579872	588584
33335	31603	34546	34661		30687		31781	32469
22014		22051	22333	20792	20104	21895	21999	21425
623432	591453	601355	590298	606786	614548	599184	580832	587898
194793	182804	195658	233366	222818	240050	175717	194853	184970
40095	61987	35599		68194		42072		70992
27825	32205		29700		28618	32837		29417
				17814	53423			
	43953	37713			43755			
40567		16415		52166	45354			
73844	56036	32669	18545		67172	124287	124942	115406
27680	399640	12416	490659	490132	500330	498515	496772	509999
45605	279737	33090	56014	305598	62049	64942	85692	80106
21344	70384	68035	84557	34385	27683	24818	23248	21614
74535	60176	245257	12792	53184	80916	78108	78862	76409
64929	79086	42336	88375	91434	80737	64403	87790	91001
46516	49556	46253	49613	50641	52210	28962	55444	55752
37182	34416	35728	32150	36181	36137	32519	30476	32142
540267	21973	37621	537947	524013	23246	19492	18235	17963
128132	124287	125228	142696	119219	121675	136423	140088	137253
		24870		16306		21631	20720	20560
72365	24419		25073				36278	39114
61447	15631	22241	113759	43010	125840	66031	64640	66387
105131	93081	296745	62877	106445	106057	68508	106796	105563
394298	370795	384227	381696	369574	370537	365663	371380	380601
117285	104582	102683	108237		111096	106752	108110	106745
963402	691851	628241	968032	1096344	1107145	1056091	1063330	1062507
					132499	131018	132070	116684
28848	23610	27345			27288	30274	27938	29461
			32335		32623		28725	31506
30495	32005	28534	30973		20972	29783		22865
98260	87638	67895	79697	57055	89764	593601	95858	95816
1719694	1467951	1468691		1571694	1808185			
33387					35514		35310	33471
	68324		49286	91853	93191	121186	105416	91736
76961	78211	94640	84514	76149	73346		80829	83528
195733		181615	295929	242549	250371		415774	404026

52993	12986		26945		49599	20996		
136996		80932	317371	185224	532827	224509	197382	715220
55878		54252	54224	52601	51426	50632	50607	50584
54667	53689	48891		54680	50957			52524
48288	48276	49616		505792	485173			
	28700			33638	32466			
184186	138706	61160	203658	111480	210835	124252	119132	110704
63179	60749						62175	55758
60812			86549		76980		122079	122641
81045	24312	28437		12720	83597			
437711	296612	360809	479103	541240	531049	511639	514717	513347
106246		29700	37322	23422	44260	40230	37799	75061
475101	523591	489457	693356	665165	807869	852175	696421	240689
351844	288591	328332		345952	282950	60219	324763	54338
1705757	930761	1537909	2588079	2248795	2440218	2753101	3986740	65431
57263		81656	82905	59942	82373		56252	54920
136040	125072	32836	136096	144492	151520	147976	140388	145820
80730	30844	112488	142500	93310	183403	114948	103383	248613
226963	213461	226074	250066	208466	219227	253129	249400	253133
46700	41401	42163	44331	41784	43502	44078	43003	
42236	41711	41076	43288	35127	39669	44984	41673	41352
	161472	91848	25990			35419	51360	46233
	30457	46422		55074	33814	16409		
13616	358726	393910	391578	424299	417463	396468	400730	391587
352093	285864	332539		367385	349921	386718		378168
59568	55866	56309	57068		5720	59115	60060	2444
		22201	21437		21634	21602	20831	
1210968		542774	606642	1173359	1234631	1403157	1420306	1325803
304251	363192	103692	303183	411282	405570	390659	740050	407948
287577	302258	317451	306806	402968	332490	315163	306065	309151
13827		16102						
269049	164677	249656	384273	275470	259948	430716	492804	450094
12971	296998		393990	437773	424408	427302	422650	413301
422953	323804	266711	311458	435095	468829	382688	408627	388082
680501	219346	1139872	1476338	839340	2681956	1207749	1150675	3210417
146070	143587	25679	44306	49504	22640	134400	6820	102888
60759	33658	48029	75824	75010	91329	78290		103794
	134920	206827	319731	238144		369367	425027	388382
173168		114934	150930	134988	147416	163852	148190	139548
111392	38271	22352	40018			54634	58398	55184
133315	31437	118181	186431	135520	147213	253832	228656	251972
165556	107552	85936	134827	155228	185012	172567	154907	154510
59427	100216			25519				
482215	68519	76471		777004	78341	79529	76190	72819

199332	85052	146101	228572	137368	343776	261539	318434	309088
270875	339792				385336	219428	203891	266337
1253756	25451	499739	27618	1357732	1245269	1303141	1357368	1233263
312444	283280		3676			237886	291184	236799
97561	78599	39979	66563	121386	121799	120377	122444	117326
	42220		42221	38432	42960	39111	36684	
153295		147670	174731	149866	506852	246658	234949	662557
103960	64051	91665	122603	98109	92051	98321	134060	96896
27735	26349			25637			25450	
		40918		33490			35835	79324
			79340				748748	112220
319040	312521		298491			294412		315311
						126708		131603
105776	33025	35915	53601	103446	39568	52127	50227	37025
600856	354196	521969	807188	578580	537680	804885	816125	799035
214620	206304	64988			4916	228251		205404
	74399	75120	84234	84364		83007	88521	88429
23888	56956	59618	63913	58432	58569	58326	58126	63517
96410		3744				95070	91099	
96597	79006	83340						92249
	243678		17607	323258	49609	300533	22680	67958
	222559	253361	306676	332267	333778	348032	324908	313936
		50948	61727	41301	116727	78707	74413	174185
36445	84923	95527		76115	78979	35005		31956
228195	156732	202251	90584		308944	293473	347334	151716
	6170	153148	536464	207352	199936		154344	304716
213980	161637	104674	153244	242897	248800			241430
		23369	6428			23973	21427	6556
40396		270937			374791			
95321	111203	62068	565167	70995	202867	88967	108008	81467
93200		39253		62643	52689		21780	
40394	40087	59341	33837	37058	41139	37329	41604	43616
129512	102079	121444	91730	105092		9704	69374	88504
8890		78416						
58787	58395	68716		56136				50161
138000		109236	102193	139448	132796	103584	90796	91095
	32448	21633	2576	50835	48128	23087	40384	43224
			1150044	401696	406684	1351504		
206404	95466	67221		131161	7928	8620		22840
160943			118813				157218	153731
94220		13657	96804			138076	136656	
	46684	22162	23120	3088			35361	36904
46068	124972	250233	14439	265444	613844		519984	522489

119165	117437	119762	258492	479486	388048	249709	242057	92456
57737	51679		40099		56840	54978	60212	55242
704872	262135	361826		710160	450554		766309	
292076	4208	422180	198792	458288	225856			6055596
59176	394520		331652				113004	
28808	22399	20771	29024	33565	33704	27504	31680	
102775	86665	70534	63648	122711	84408	77677	44854	67842
					1403320	1513980	1401896	
8508	21855	29596	5609	23626	20285	28412	7852	
	126900		188216			5177024		
118978	100183		83116	124534	118301		106998	109296
				196564	1280631			
53046		419270		2176	934931			
89886	73039	52771		113280	94794			
						243708	202067	202662
	180227		190438	254579			128184	
					247398		244023	
23960		117641	41580	33996		39916	35868	
812168	616787	112468	983996	860560	725039		841495	878787
6124			4832		1318187	5244	6588	
9820		86425		14640	495796	9704		11988
2151976			2889656		2319402	3093540	3068339	3076900
99584		67662	55186	70597	78590	67395		54146
	308116			180200			579804	
	782645	1006244		1119844	1086476	901531	1082588	968223
			6860		6337			
2731852	2124036	2190774	2064869		2576394		2806157	2817139
24908	11992	16588	14921	24744				21076
102392	479556			153748	126148	1804		
218811	201080							
12644	14748	14004		10753	10224	10197	9358	4156
	36970	24822		54724	49484	49574	49413	28203
	44020	77726	99092		91018	103629	111536	119523
154448			112754	134849	146885	133557		
143853	120456	93951	125937	155714	156916	151949	153120	149606
	22992				17824			
580873	533795	530000	619022	586362	584931	623804	599093	609452
			5720		6380			4720
1722266	1509537	59480	1911320	1796292	2051400	1859509	1822111	1884641
	179373	139022	123401	71696	86652	263764	258075	254098
					28808			
505526	524741	566179	500702	647547	574522	559328	631086	639549
1433821	1298320	33152	1187701	1586055	42836	1652647	1629083	1684182
12020		49945	10520	10869	740		8769	

9660		8136			8546		8074	
6754		7478			7060			7388
129020	120652	87104	102992	108619			123512	109119
95274	103324	65107		123524	101898	85740	91520	42669
20067	20176	13672	12899	26503	25996		20260	
		18288	22394			27408	21625	23426
24081	19217		21095		25270	24702		24853
	9220		10928	41137	68579	6984		
	14761	46682	56644	11642		58420	6903	58407
65497	58188	53824	34968	47556	85424	64406	67488	197216
32161	38866	40277		41520	39202			972
96521	81544	68673	88106	107787	105908	97235	100516	102411
17136	20108	12124	17108		11514	12488	14792	
	346233	344536	403022	400609		495464	401858	409563
15230	14511	11520		15423	16959		2425	
13185					9972	7440	18080	11567
14232	32637	22568		14524	20876			
1024769	886370	1009396	1002692	1321292	1093551	1109593	1112788	1150955
		9792			8670		10468	11596
44992	42636	26526	28748		35461	37399		37091
36748	2388	25292	21936	30832	30742	2856		23951
247796	188924	168933	135230	217807	239300	194355		178851
108447	86339		67904		116444	118518	111702	128412
18580	35359	17879	14402	31650	18672	14925	19072	15921
20492				20892			16274	
300926	326599	334237	298063	413629	411452	326533	403267	398198
9085	11142	11051			12053			
	6615			6300	5918		6984	4469
823969	786379	21800	671948	937432	929979	10668	965106	156396
11208	9616	9396	2812	14576	11096	9690		11302
174197	190032	159461	129743	234365	279904		443664	425982
			6686			13957		
		8754	11312	10412	8214	6574		
12989		11825			12351	12926		
			10986		12256	12877		
5974	7003	7356		6892	7485			
2388		35072		40770	40174	26343	27530	28133
35166		25945	32255	37614	35781		127976	35809
114584	81793	81624	64121	94900	103875	77504	88817	84672
65058	57075	49893	61405	67221	67815	64385	69332	66559
243072	205988	199535	250593	251797	243420	264006	253801	262172
			3060			8907	4992	
17262	28655	23641	10843		10358	12619	12559	
582632	499126	471425	605930	639408	611487	652259	635802	666103

46369	42918	56249	45248	48067	75406	43450	42318	123022
9016	34045	9416	9562	38478	9284		9654	8832
117358	47746	40411	49182		138109	92046	119668	125045
	49117	52292	54876	48560	53477			50543
165683	2556	192339	162813		198281	175169		228830
		2508	2680		17941	3396	19863	20722
480732	439683	452355	368247	565747	553896	528046	530947	532150
7263	6903	6439	6167	9596	7463	29258	6316	6259
9253	9042	8607	7444	9555	8299	7277	8319	8423
37113	12259	5518						
117248	117952	84009		117764		89302		84329
	31204	30906	35980	33014	29736	34085	35097	36994
23348	18400	18806	23820	16924	17654	16909	20932	19846
70834	479089	141778	149839	147505	162351	137858	138344	131285
155584	257813	87717	144352	148926		120542	54586	
		12546	11987	13084			12074	12945
18517	15705	15386	20540	19397	19100	17781	18781	20277
38977	35622	32997	38330	40000	40224	38765	40697	39957
	14958		9829		9564	10656	9772	8106
141995	101489	135348	150402	163828	141646	150201	142107	140933
18339	15799	23282	14460	34529	23001	13470		16286
233176	233507	344744	453586	409999	291011	230374	213568	192392
33588	93056		15672	37019		40443	46450	
51738	43213	24196	88142	88863		57730	29209	7784
					13451			
		17097	5100		9780	14188	14079	16887
26500	60008					7753		
23752	275895	117580	15916	11991	137176	10801	122374	143760
23797	20487	21297	17821	19617		18632	18703	
14976		105509	6105		10633		10235	7240
	106030	65408	27174		10040			4892
20952	19737			13937	11893	13831	12486	
4907	36276	27044		40188	10227	79012	14208	38884
85204	137752		17854	16853		17162	11858	
16929	91026	45639				10163	11350	
						21930		
765110	1867371	443435	491788	68578	154083	619712	227044	209518
119442	360292	1206938	220105	76435	1824	118847	75196	198066
200212	134066	161668	169763	202308		172893	166330	172843
20238	12713	67362	7394	9601	14113	6650	16592	13177
	50838	165572	33908	21195	30384		21132	15216
45218	57519	49821	51321	70123	61465	40172	61514	63709
337780	97375	18220	348915	30933	52295	200707	102919	75465
694604	886310	339914	328549	623890	11144	574024	621296	128597

249310	235076	794735	43967	72915	81523	31082	38070	72325
63767	83819	67197	907798	94223		108955		143306
		22571		141125		6934	18689	
69344	32692	52294			79875		24245	2676
50031	35792	166884	34349	50408	33668		20406	
32155		18988	30228	29517	8644	16823	8204	
	15358		15608				27020	
6635		61311	3905					
		10562						11205
24124	283384	10946	43884	33784		488306	501540	43252
	16737	11109	15289	26376		39227	41984	
55205	340487	315744	48887	121285	50263	214627	75921	87183
44295	110720	31691	42881	47127	14169	52568	38734	
233832		823335	86551	160610	121423		77708	48710
13903	77179	17853	13961	7793	70490		24327	17753
	11305	62851	4872	12660		8146		
	31291		27856	34340	33537	27630	31432	30556
66330	64336	55329		78375	75030	70346	62686	66655
6540	88047	8674	21286	69302		159766	114054	
43414	5793	25214	24628	29050		41448	32426	
425813	20503	387886	83565	74756	306691	6364	74553	54918
48482	13703	83939	150534	40405	41013	11734		
50305	49794	25392	23697	43133	2852	40321	43239	3056
	9006		2573	12014		21252		11930
35268		20363	9595	9707	26906		10037	6913
22456		16033	10627	17731	17470	20744	16974	16524
35183	21796	30870		36318		33947		32744
	5085	4691	4158	7200		4200	3428	3813
13884	23700	1064		17164	15690	9245	12898	14241
34224	31523	29024	23126	40532	35807	31365	27274	28319
8286	6156	4040		6000	4951	10698	7039	
6458	3454	15495	8274	7892	6325	6991		
13922	8194	16432	19004		16963	13289	17224	13894
6708	6490		5923	7299	7444	4489	5594	
14835	17756	11237	10987	14162	15364	13003		12890
3904	2222			2628	2134	4245	3328	3099
8504		5512	5472		6244	4885	4636	4876
6094	5861	4218	4499	5645	6507	5353	3720	
54499		30341		68411	100657			

G44	G45	G46	G47	G48	G49	G50	G51	G52
	130526	126009	172434	170620	80778	159001	106135	
20417	14984		13631			7228		17004
22411	24424	23738	20887	17329	22148	17978	20523	20005
11501	12129	14277	13751	11679	13089		11545	15072
	11718	20147		21888			14945	
	225108	704899	398383	588613	583534	556335	659769	
	36173		32824		29965			
	24485	23104	24078	21002		22647	26540	
12575	10945		11060	9400				9673
	123098	256312	150572	223920	51669	180379	24879	
12519		95787	83185					
21860	20780	17772	154392	16724	16588	42919	74529	14570
		15170					20820	13768
35866	32262	11481	23162					30206
110469	130892	85964	93461	65261	62664	51645	51993	93619
	533356	435569	427022	424525	407514	410773	332211	
30584	31236	21859	29761	27945	28280			29550
	191014	331464	161101	132566	244147	163172	159027	
99116	107017	105000	103257	88344	94023	79234		77730
14594	12245	20323				19715	17165	
	226700	687680	394156	579144	564744	541745	626578	
26898	22716			19967			17144	19854
44579	43307	38557	37729	35348	35983	34530	33397	41967
156159	144984	119301	119457	106774	104072	103133	102424	126675
14017	15395		116494	11432	119113	10919		13655
		42518		14093			32188	
113384	99733	67193	75210	63542	70020	54175	19801	98450
36200	32875					21769	26095	29638
	1656063		1886402		2377453	2085583		
65082	61702							60370
10260	31065	22672	52785	21065	21554	19122	19926	27004
19602	19812	13960	16138		15495		12699	18943
	288066	298230	297213		398950	268652	380177	
145503	166228	946028	781887	1066643	899284	924212	1115320	
			31598		31661			33256
29818	32000	71987	106241	32289	98035	91393	86991	138089
324518	25238	23873	228368	175349	191047	149120	167839	256507
63131	56500	50304	52392	41669	45079	44981	39119	57939
56055	54125	49997	52353	48171	48542	42268	41587	48409
26123	24823	21885	24011		21231			21411
74300	79129	310199	72577	112955	113278	63478	107369	103735
39552	146853	93016	36941	34933	35460	60275	62350	34474
	43163			51083		53293	34731	52999

22685	17680	18072	13621	14358	14676	481695	482018	593010
120502	116544	101082	99888	91252	89450	81623	83159	101026
38027	285607	197382	217661	174413	218628	202705	252995	278722
336652	29525	305459	302393	284177	287978	252228	278422	284105
252648	243129	220020	226788	194029	184311	166239	158792	187501
219401	175729	71905	122319	26074	28952	70236	26186	158408
		158374				188580	161463	
585558	528071	403956	396089	358665	370000	220988	327942	418806
31855	33012		25332			22764		
19956	21012	16923			15089		14724	
559729	558043	521304	510027	467868	476877		452813	506975
190257	248461	192654	175853	251600	214151	186156	208109	177928
47128	60066	37876	76536		37535	36390		
	27961	18327	19022			25467		21536
	40855	24594	23177		63553		61547	
		84880		33145		37797	32031	
	43317		22648	57762	96664		96461	
130769	70882			86557	74637		55176	55512
111641	568830	104429	89524	508696	57168	418454	370757	2987747
95272	73656	85778	73396	244844	58017	221776	255648	278410
77496	65411	30307	33623		26049	46243	45253	
90211	110637	465280	168324	233036	74748		537164	58681
90830	96316	80617	86875	71827	83486		30096	37971
56546	46121		41627	13932				34796
31685	35539	32719	31941	35377		29220	57076	28699
528151	483683	359910	32713	24825	316801	284833	88452	431344
139361	26003	85560	88092	80449	78193		70090	92381
18276	20091	17780	23068				15324	
39997		37064	19151	20136		95114		
78453	76408	83378	55655	65645	146157	44028	146161	214653
115647	116010	105078	109306	71855	97295	86577	232234	41049
356406	360656	325409		296951	301451	328552	287192	335315
103439	104314		103448			72274		
1319356	1530854	1464014	21399	1203389	1081712	845928	672773	690560
				54096	87142			127392
	28069	24514	25830					
					17310			
28896	22327		18437					26795
95200	94622	106884	80950		72002	55035	177972	76834
	1445104	1407100	1422726	1819131	1870179	1821098	2156950	
30677		49782			41726			40111
140746		173563	161411	124017	116405	83277		
80215	88592	69174	69133					71335
423854	249040	292109	262064	192383	193496	151906		160660

		38477	24197	17185	49310	17949	43712	
721630	674306	630067	421571		343460		185308	
45352	49185	40183			33795		27125	40513
50628	50728	40774		30626		29364		40802
	483750	405304	400834	469735	524849	489615	409483	
	27482	23954		30935		34217	28779	
270061	271940	123860	250141	206483	190884	135662	118559	141826
60063	63257	58279		48556				
125271		99335	77123	57172				49474
	78840	44224	83209	75341	38581	41452		
696689	778004	725302	695783	526543	461573		261462	273868
78358	51828	52156	39630	20177	31922		17900	
843458	843203	217791	826262	147447	159223	138737	759444	821756
65096	76482	314108	66377	265330		234567		272823
4250979	231836	142196	2773724	1917668	443362	1504076	802504	1294249
55919	63722		52812		47365		2220	50137
140252	135768	152560	146260	140400	145356	135884	35445	132536
251518	190983		148320	88401		32244	77619	23334
251714	203085	54630	82391		30742			171020
40308			32617		23006		24183	33415
40894	43089		30291	22441	23774	21591	20277	
60537		170606	49556			175585	167086	48587
	37389	57328						
399033	28455	14934	380473	21029	387762	298103		307053
475724	379625	366779	389142	330467	333298	286481	244082	311449
57055	55696	45072			40335		30976	
21411					7891			
1582049	1505379	1475177	1473822	1297722	1141134	955319	38088	676090
380773	373407	218012	168559	506232	850227	637918	836305	552116
307145	265840	302873	301823	279595	312889	194770		216954
	32332		50540	120128	39224		78907	15518
493096	245837	295944	255588	169835	178159	208495	86120	244062
492318	529591	490683	497617			292478	273431	290127
426172	462195	435325	431649	534982	549917	293275	574698	253305
3389773	2559986	2218814			1895284	248812	909408	127413
112008	151080		40087	45874	151924	98886	35411	82349
	95781	104268	93196	70795		46473		36458
414983						180585		214908
130343		126990	167812	164184	155704			108537
55516			58039	58974	59053	58636	56098	59421
242831	152694	169187	150209	83342	24264		58116	32478
211891	282412	295844	264408	222632	197360	109316		88003
	43175	75301	69387	68544	69849	16445		
	75233	64879	66835	54374	1370260	47563	952740	69674

339033	87029	402544	307730	270577	292948			140171
	311112		171832		357244			202963
1443824	1560258	1552755	1547574	42870	1383912	35699	49372	648225
280624	287332				272356	246280		212708
136464	142959	143019	138722	128509	111790	83046	59927	
37204		38264		35980	33408	33684		38808
748658	950766	1153575	327523	117336	446854		137925	
128402	104686	173686	98539		44250	75998		91216
23183	21907		22633	24452			23716	
	41552						17868	34476
1004120		837074	728584		2068624	500438	371080	
	294567	293266	274509			232987	260660	30016
147133	74597	83255	76164	78155		65347		50068
39290	102372	50657	77268	169660	153577	92024	168493	93410
795184	487682	576724	501124	371461	412521	480741	197127	578218
242692	260928				205261			
92933	88546			88056	85567	70798	72384	74642
57817	46921	54351	37286	45354	43660	47331	63292	71976
91956	20773				114976	101652		84778
	100212	109908				78732		
379980	360070							
74032	76880	82038	46138	314086	26164	244760		246222
346762	399732	357512	347045	325989	295360	240088	220277	226333
184830	136957	367146	82994		72244			
46860				69135				51588
	368400		149108	272777		127488	154563	178057
	1280292					716518	467727	529154
	73656		21914	27961	242446	184133	130224	154154
		23089	30088	23710				
		430806	199889	314958		731859	800501	
554484	297091	387188	547009	70206	452199	308523	437951	87061
		20308	282544	639794	74208		317125	
31359	28647	38881		59242	34602	24825	22119	41813
8040	87425	67948	106236			71935	77672	2700
		46440		35080	6856	24897	46927	
51084			49980	55785		66592		52437
106584	117504		116792		116848	85962	31133	74439
3548		20757	49036					
		301584						
	8888		56907	43214	106707	22908	31103	
159938								116808
357227	26608			152360	146048	57917	93908	103604
	46064	10190					38287	
	251520						320676	347226

205881	239803		131387	198188	99608	189957	64864	202162
	47835	60560	52863	53909	42801		56776	42019
820296		742940						
5187732	3947365	137380	518000	452872	566012			336332
		434974						333689
28096	43140			32314		27001		38468
89895	80770	70274	117388			179181	48582	75776
1439748	1428592							1502688
22510	22932	34004	28940	26632				36448
4538729	126300					323748		204792
119648	111568	88613	113059	145764	124167			83159
	1041580		991296		1853400			
		2027181	222786			56053	1256472	
	87234		92368					
219942	213448		202996				74520	143138
142576		316284	361720					
252391	260106	278176	269223	284740			15751	
		43808				231133	186536	185203
786249	661188	762313	775406	53444			418915	280888
4477		1634847						
		49490	811480	7280				
3032948	2458063							
51595		64779	67078	68325	87291		58733	62337
594923		261336				524064	385860	4507
862963		1066744			1039932	303264		
		40837	11428					8636
2774406	3015468						1790168	
	17616	18968					13231	19040
	99480	126800	199028				647100	
12084		14992						
45793		46737	47454	55877			2584	40175
121155	106467	118587	97719	81853	88632			
78564		145319	147015	153711	308176	133199	175628	
145389	150854	165137		180695	185875			
		65789	7240	28036		15111	32156	
570289	522365	562822	553646	588064		618239		20568
	10789		11212					
1872814	4170	1916545	2180824	2242220	2207540	2101712		1516300
253796	226229	254243	237106	280628	240844			159697
			18157				15880	
559367	586870				642664			590695
1603245	1668243	2090508	1680251	2086596	2037584		1048444	
			17869				90927	

				10548	2876			
	6817	9484		5372				
	110236				127652			
	16748	84055	92493	16940	116808	95752	104220	
25647	22726		29105	31104	28389			15056
19826	19146	21931	18628		20117			
21341			27764		32404			
	42120	29800	65898		82419	1824	8930	
7790	14933	9613	13386	13299	27733			16204
61235	63920	80624	265896	109012	77094	67896		40476
	38183	47399	43265	45666	41194	43238	45262	
93779		100696	97711	114193	117616	101814		86462
12710				15976				14902
382831	363929	363748	361186	485316	391551	392749	428272	357911
	14409	16125	19616	26608	30616	29306	33722	
7888		16169	19120	15464				
16969	28996	228680	2456	77592	18896	57124	101819	13808
1124876	1327640		1126814		1353572		134108	894823
14900	11561	18191	10684	13734				
35794					59292	36690		32319
26256	26984	35636	28045	28296	32036	3416	28565	
157369	202206	243562	236827	252517	288747	334633		
111628	113169	20472	11124	18880		128484	70411	78803
29778	17205	47826	41337	1740	31136	59363	67426	19416
13949	17760							21888
348277	376291	347944	365781	390206		300655	220030	372195
	12071	12863	14844	18034	18849	16756	18884	
	5447		5507	7004	6328		11248	8468
886882	976194	1013007	1006553	1014805	1269512	1032872	11635	32444
11452		11112	2184			15984	9202	
598768	390496	458096	338432	449412	228998			205924
12470	13730	16168	17456	14627		15704		
4516		8028	1720		7784		17004	
9225	10596							14418
10701		9253	9984	13578	12977			
		7187	7462	7795			8084	
26067	28022	28149	5000			33300	24467	24684
37176	28827		30605	37982	38592	38001	67165	56685
73207	95320	83885	93431		127692	140453		74636
57589	57654	60369	59278	68714	1960	67498	46166	63143
239634	239866	217602	228057	253675	248837	224940	211362	216625
10734								3764
			7137		141907	50343		28968
620498	647942	621166	616187	796360	645517	567516	555934	9744

45138	44414	87678	68701	52445	72153	29683	103521	48933
	8415	41580	43093		44361			11030
116923	268914	102006	90396	104407	163060		214932	56780
47048	49965			53848	52786	56029	55908	51407
201182	213935	206359	209205	228448	188843	174291	184463	1652
2263	19597		3000	20428	20297			20291
489842	569031	608918	582220	604996	616648	628059	510396	485959
6525	9056	10852	10517		10159		10455	10870
7467	9345	12343	11672		12492		5584	
					28734	10141		
	83221	106916	114692		81382		69018	109808
33263	33009	19815	28360					
20556				18848			20022	
119152	285145	234152	126099	71384	174988	682902	151041	542375
				153536	53515	298105	55080	492280
11389	14466	11739		14792		13168		
21360	15072	16999	15071		20727	24512	20836	18499
35045	32822			41676	42405	44086	42153	36416
				13372				
134637	135749	131130	127915	144900	147229		92759	18794
15640	23945	24495	22922	23232	10817	24285	26184	17352
102523	197317	246002	351114	426293	353231	261500	268651	234484
			15260	37598	12063	155402	7705	112968
			37806	103460	15494	58997	11659	87867
12480	13613	13576			15820			
	18208	16184	11101		18780	17278	45832	16797
	4826			8623	11434	101129		
131160	115648		119832	33836	3994	358622	15541	138811
306896		21728	14875	19042	8779	21691	305068	19964
		3604		7131				31312
				7226		74363	40982	30686
				15803		33012		20135
8020	7743	8049	52889	67644	84048	98508		14336
			15530	76920	41202	282018	40917	85181
				15904		135618	40381	75348
18266	16670	18868	18867	2968				
6413	5821	5440	259806	47544	159647	2060388	532203	1797414
128259	73463	67594	73250		223002	187737	603400	64928
151514	175751	169135	174716	235684	231852	952		6320
				624			55879	41101
					17492	14940	3132	123500
54396	58217	58932	64962		54078	55072	5464	67118
			67934	954526	90040	116756	123310	508396
102970			244257	721537	94129	1252133	427348	1117034

			11471	17058	96667	116948	200634	721505
86749						33047	406860	16678
6256	6334				2324			6506
			1988	15791	41271	30112	139737	69606
					21243		167624	
			9698	69538	13524	54027		33263
	28896	3992	28271					
9092				13568	6690		28166	6918
							11882	
30329	44440		309676	652815	132788	545482	40052	540197
			20234	41009		39162	3312	39475
			32184	99121	59606	420675	39218	535618
			18425	30549	8168	90086	54731	101618
7310				25408	70881		686656	421104
				10481	36990	108229	195789	73837
6387								
28544	28963	21711	28012	37643	8196		34616	45760
58330	81461	81169	83175	91651	86172	98428	5216	72523
			30585	68347	8494	187911		198710
			22370	24907	12196	50254		52317
				83205	32283	13192	1678737	268478
9773					10500	7876	113980	45193
			13541	51630		83207	11020	72067
13099	12398	1856	14216		21225		4368	
				8191		7857	92445	21843
12027	15992	16269	15917	22448		19780	16872	13553
27319	39132	48988	40422	43386		34140	32750	28205
3485	5593	10856	8931	10343	8089	14320	10861	5840
11779	12302	12063	15909		13275	34359	22476	29654
25930	34620	44048	39201	42084	41246	44783	50416	34354
6552	6494	14388	9660	9111	11891	14523		
5475	6367	6874	7324	7522				6314
11616	17353	20680	18127		16630	18224	2276	12109
4441	6696	6652	6619		6797	8473	11408	8224
	5296	15358	17334	17579	22864	21768	20904	15489
		6001	4580	5900	5554	6772		
5644	8098	9056	7520	7861	8916			5135
4458		6597	7448	7940	10100	9898		6032
	125629	101506	138943	371198	334903	58052	4668	

G53	G54	G56	G57	G58	G59	G60	G61	G62
95497	116808	88973	95273	162444	137125	120246		127134
							18674	
28100	20835							
12870		13199				11601	12478	12897
18023						12891		
795842	689555	1026778	879009	1120149	1002699	907957		1082457
29921								
26124	28072	25510	24577	29266	28449	26160	33018	26674
12137								
25598	224753	23483	234723	17734	199729	215142		
120902	93672	99612			118242	79542		128663
76481	100815	101284	102307	67986	77027	98160	20507	18468
			19867			20150		7928
							34555	17020
49001	49223	47446	42452	57316	8988	9341	93305	8932
309530	364755	354197	345356	374893	409277			409742
29222		28097	28029	26839	28299			30003
187143	209463	267490	267305	227971	125533	207971		201390
92598	16393				63014	54399		74398
	11687	12535					12280	
744690	672758	961488	724537	1054395	964777			1070125
							17975	
36237	105514	115896	118281	116842	113950	118314	138381	119420
98138	105095	96317	100056	102283	96962	38836	131957	40291
11156					118260			
27996								
33156	56251	45942	48215	40079	45157	56021	100498	69762
		26359	26670			14858		
2689294			157274			197284		2897027
							60946	
76396	27707	29408	31192	30101	26986	27483	48072	28672
13903		13068			11926	14527	17772	14500
	310437	291476						
117840	125262		1110216	839572	121502	876284	106048	187103
	74823		80191	79365	81291	82044	84111	81758
42615	79317	14080	77964	72698	89950	97349	134219	68929
158579	244715	246081	253758	256327	242182	258376	388310	307242
46356	36182	35730	35570	31632	32882	32997	42200	36542
	40617				39459		39613	41161
							16571	
118951	275548	278706	265858	36583	33932	261983	235010	266006
48923	49843	55693	51993	51733	41123	50535	107722	27899
50233		52317	44148		33295	32687	50743	

451045	489212	446617	438038	450957	431386	432915	531073	492974
90865	76380		76103	72610	67117	69896	82932	82510
317799	188696		66180	63929	61139	60972	33584	77679
231499			337907		311391	309312		
156921	104676		101745	97005	89818	75082	135832	132450
26220	25852	64387	62192		36915	58506	145931	71812
182185		158119						160301
101542	42115	203307	336331	332741	320723	341717	483963	194695
436785	337026		309884	304159	215819	289812	323949	315835
186459					99623		132586	143403
			80584			53558		
134687		28523	68223					
33465	28216		73876					
132276		24758	83661					
51772	71460				28060		24159	44705
393390	219598	221784	267759	285903	251683	227418	57120	341768
198409			165679				67387	89542
		38093		56404		46770	53938	
70984	72304		84010	103335		94800	65342	122309
67630			26771				24153	36450
						18634	20704	
35376							16903	11196
290414	261507	36819	238695	232137	218261	230989	332048	269792
	66715	62733	45121	65404	57693	62226	86577	
				53583				57698
				117368				
345572	74702	68398	168090	78174	74893	49167	54284	61557
80573	174425	185892	368883	438503	304688	260023	20107	25579
269684	3098330	4006044	4074864	3929640	3394542	3992996	3910210	3675933
	87219	71441	67252	72970		63155		
768804	789078	241659	706001	1266920	716192	376009	570383	1451809
							554427	
			18884	26031		14895		18964
							25167	
193319	64247	61546	111707	63632	59849	60683	81285	52257
1577649	2202110	2412705	2229526	1764865	1480957			1702173
22808							43433	
	341492	411764	434300	419884		361042	439554	403050
18898	138364	157851	213654	361144			494244	518330

68265	17057		23795	31228				
							205349	
29076							16872	
415857	500919	513235	535407	423134	447650	478338		403267
32690	33393		36315		46610			
133280	62680	105327	142293	124400	106387	119164	29326	198402
		79203					78419	79737
						88562		
				48319				37786
282155	124807	63297	133210		110053			223955
26201		14945					32069	34420
792433	134047	594054	602238	532863	120577	577752	622014	582284
	254940	417303	487965	482862	505666	511196	570291	623817
1023036		1152488			1917950		3903736	3971913
29454	91925							46362
126656						43434		61788
127309		73988	83085			42819	119775	136869
			28900	23350		30013	165956	33653
							10233	
							19645	
175239		159523	157732	131396		158845		158472
		31751	11520		32017			
280448	234941	319782	377167	356842	346074	340145		419911
317580	108092							
	48680					43528		
	54076	55576	49710	57519				100536
95332	622287	128518	245180	516625	360571	206375		111336
1226008	568674	167488	719474	10939	524208	533822	562653	115545
	240148	253736	291370		248989	251601	277481	258349
93739	86376							41449
95064	198405		282615	497263	469265	516088	712937	626743
326860				205004	166035	209362	165285	284650
551331	143414	204420	269553	146072	109536	78994	12745	195077
1704740				320124		302876	1155887	1408156
	163472		33862	126534	102425	48756	16912	
		12840					18788	
		200099	158321	408919		435281	609789	531863
145448	124376			158976	102236			173940
84861	35597	47870	44802	47091	118205	35255	45660	46353
76825			56376				166104	166825
106168							46030	186548
87036	41991			83992				
1321272								23824

205252	98505		130037			94936	173409	154452
30902	60224	35778	33706	134247	349264	149004	276641	772423
					496116	648848	632910	694840
75289			21811	53948			22695	78637
			30816	24842	33492		34240	32024
262117			86647			49754	115338	
	77768	88260	99194	160558	158040	182808		186700
25740	22232			20564		16344	21958	19942
		952872		29023	637376		2690604	3297484
248685			152755		146038		195900	194868
11351				45776		29240		56657
175959	176209	167636	167090	171893	169317	160214	93456	170142
237982			530850	891052				
183448		55299	281144				216592	
82849	63860				43814		62496	58427
42659	49703	53664	53262	49159	49428	55567	68522	23756
126244					87640	82260		
			80943			53013	70815	112330
274282	187458			204963			32066	36617
274338	193285	78484	200339	242988	171829	116471	166886	322066
			122991				50328	
251100		94244		220505	180252	187077	287917	321755
4692	517261		204623	499114	286534	164657	241087	598958
161726	139710		50487	132560	76078	38508	57871	179709
1110102		100474	39580	260624	634787	165555		202408
554823	458538	450770	435842	495611	340493	426060	542502	456094
188431	23044	71548		721387	706961	77877		
						3940	52728	
			38368					33820
			32420					
33536			3800			26897		
		484012						521576
			44496				10028	
			70800			51812		130044
	87910		90394	192140	88637	190264	211268	256540
					37488	21856	33972	61708
448620	163013		323000	235876	209875		207316	279712

	168765	308384						46192
							14336	
		238281						
								444908
414679	442692		246120	461009		321423	437796	577107
				23031	32804			37637
36185				33038	33123			
21824						20722		20284
								23819
	42314			40625		28252	30496	27475
		1234252	572500	133410				354043
345274		119338	56411	3369195	1573854	201765		129539
				93785		35980		
	49549		148824			218316	247615	257337
	277395	285479			265117	296321	280822	
196318	216548	67286	119925		134144	88701	108959	213523
461750	491524	494488	555948		342178	355396		
						98070		60645
						1032616		4628536
	34168		24960					
479954	564004	261623	318939	573308	509701	411529	478023	597275
620869					503204			372108
						52160		62254
						66732	228978	
	28724		18364			29785		
869814	312242		230044		222372			420269
				166662				
			15408			13528	14776	16088
		6873						
62326	80925		89976	120408	29011	141344	138959	150137
144812		49626	86615	362207	107716	63014	65222	359072
		15387	14307	44974	44808			6356
				127848	807972		124244	1072616
								8292
		539660		110248	1607180		2802552	
259774	297214		176318		278417	225653	247176	331467
18993								
	483622			263064			1207192	1210672
		209700	1711537		150484	238940	115520	56864
9528		6252		25980	182796	20956		23128

			13808	16548			12244	
	8751	9076						
		26139			39859			1100
			22576				20909	30404
21545			28612		27924		31697	29453
								24931
28974	17003							
151931	239624		45868	60824			35573	179988
42955	44866	46143	44944	36860	37771	32049		50582
128696							60929	105162
	89432	57724				51576	55977	
492272	415889		634776	79868	651860	548620	661254	771036
38265	37555	40682	41304	30267	27932	24157		21390
	12110				5124			
101379	43512		50464		138072	59156	10100	108660
1261887	28364			1402413	1404302	56540	1634737	1710241
				19944		12664		
		113384					29972	
18028								3760
174310		61392		159698		55464	1808	
121396	143495		110984	146340	127195		99615	141692
68894		43222	40924	50828	43435			29133
249683	467916	511900	413424		439820	712536	620614	823776
19640	19793	23259	22248	22217	19534	19097		14757
	121584	13604	113424		43858		30648	35865
808934	13549	713047	1308676	15354	16029	1571108	31592	1473313
			2904	3128			17371	
				231380				418428
20260								17024
			6384				3705	
	16648	10773		3368				
					7283			
	2712							7923
37315					22216		30236	30832
	38480	102348	102787		82925		22672	36903
		3216						
79805	64369	30770		68672	59716	39977		73350
260287	221587	244589	326435	367200	329403	402468	387248	397560
12256								7200
533856	125278	2128	369311	25475	9893			
707011	659304				792197	786550	1006844	1033029

108619	84123	80956	83393	79039	83337	81951	47480	
66488		30300			52304		43580	6664
256482				32480			19294	38166
		25128	25016	27608	43416	27521	2872	
125119	200621	190291	185952	243287	1288	319292	363121	391292
				10466		15724	11270	9011
568302	704087	480829	531676	1126292	113640	699170	799977	876362
	15112	12204	23548			12848	11756	
							14462	
76997	19442	7683	54043	34294	4732		5543	
98588	413188	855932	903200	888296		802991	834645	938628
							59266	
	43467	44596			40710		34401	35565
299681	1245374	58119	140783	566002	324180	54526	77281	173929
	272937	50881	91576	328056	239295	64809	149383	101861
		13654			8084		15884	12498
	160264	54388		194648	15319	61686	70630	
55922	46383	24523	5955	59000	38296	1512	32769	49069
151334	148240			215117			249622	237213
28606	16885			22937		9612	14631	24542
390838	1800	332579	472617	546128	472518	485945	600025	599475
	217799	13908	16009	97820	45074	2520		
	25760		58486	43348	28549	20778	89338	69771
19956	2372				17820			23372
					56921	8219		
60126	84412		49957	65266				
44033	422889	63887	12361	262557	116428		15052	10450
7510	394305	232350	11756	508752	448661	380334	454114	529303
		40153	2668	6272	82761	94906	7348	10830
16621	154566	17284		82660	21702	46001	29623	
	4592			21884			8867	16985
77220			2600	113604	1696		10051	6872
127561	173965		52539	121422			16260	11100
	363035	34977	7816	161521	94773	18630		
								29370
164447	2343651	437093	29455	2172172	1604161	398471	12977	676396
997124	2132	1945260	244285	56103	821021	63585	61971	156344
222027			346876	371500	242342		317676	362924
17774		50158	35714		106363	71719	27678	12300
	20929	125464		32993	116522	87615	5959	17762
51192	57702	59893	44168	64392	101556	91515	101469	
92003	85135	6596	776791	638789	395687	261043	555895	809186
	1158478	1396833	225034	1081958	1019041	330269	295663	294454

	353367	80351	4200	310536	242357	25207	42401	
101699		107994	1407926		93811	1124544		123399
48250	3320		128044	118042			124863	140151
	10888				44342			
		115709	3052			114940	30984	27152
	17006				24254		36372	61956
			28068		3532		23936	
	3388	65892			6484	57282		19280
20919			20000		15230	13348		14444
49116	132805	46236	5372	103171	84793		73524	8822
							10208	13044
353934	129491	30706	21054	354075	518328	15397	36851	
47672	106874	23890	400593	106772	73564	269517	14136	10565
201373		966129	129782	70644	631453	927196	166943	154990
11865		5296	3368	13611	8899	34386	15113	12304
17358	10273	26567	32731	9635	5624	3452	32325	
24081	42776	31330	12619	30729	52702	2644	48024	60618
89025	113656		66898	133869	137100	2736	107117	128052
34371	6943			11377	92071	8917	15872	22821
23350	46804	23155	14630	4024	38886	25292	15819	15629
6987	31946	349325	71841		176583	351576	93231	69427
105616		276550	540351		45504	70950	128364	
4888		6904				5995		
13867				38260				16578
	3756	18385			9319	16613	8506	7339
12691	16008	18173	22845	27044	4200	26292	24718	
	32245	40199	5712	59288		4136	53197	56595
				12906	12655		8792	13022
16686	16711		6744	23184		21121	23005	26044
44782	63044	31117	32870		52031	43232	46222	54582
8871	14875		7756		10436			7696
		9016	9519		10423		9880	
16084	11738	18785	24073	21643	20160	21683	18357	22568
11028	11560	8041	8976	12088		10520	10594	11768
24540	32920		19980	31816			19703	24222
4907								
6302	4852	7043	9239	10065	8493	8419		7920
10211	12643		9068		10652		9936	10718
490393			329868		12136			

G63	G64	G65	G66	L1	L2	L3	L4	L5
152462	170496	92617		200588	238384	204618	240170	523811
			19423	25948	22145	30338	28523	28657
				33804	34062	30927	32209	34449
				14616	14458	14834	11078	
14775		20594		28552	21806	25100		26287
1312270	1016139	1013734		663441	714423	264406	257559	298040
35476	35267	15218		52254	15186	62137	58176	58455
27573	23006		27495	50757	50206	48359	61630	51963
				16857		12781	14870	
27124		37293		422396	516748	26034	189169	150536
126739				16201	199513	87736	82610	85920
67495	512683	448734	14719	36915	28219	31727	34491	36991
					23930		22783	
			33790	39383	29526	41624	45231	46199
47819	8460	54024	92942	161217	134749	161132	164022	182025
375395	395332	358879		748214	798391	784642	769939	668813
				65390	40646	67798	37289	30124
176925	179004	337581				161389	124661	123007
75983	76404			160076	148754	156424	152894	160342
				31522	22187	23476	23581	24566
25865	1009954	36940		644990	716630	269268	260753	294699
14675				30187			28511	29978
113373	117522	111792	134694	95985	93487	94155	89594	93819
38181	104246	98993	132784	176653	141030	150144	160894	171115
		106159		258891	267343	28184	26166	277097
		54565		28452		58698	50005	52771
28390	43354		72768	124131	120679	109439	113574	123422
			26893		39690		43224	
2814350				116937	140876	1682583	29808	
			55695			91037	91875	97440
27927	43336	26499	36990	75640	68643	18476	75000	75189
				34542	33474	33559	34567	33040
510477				468254	400177	391029	237274	318560
198249		183979	89459	1184540	1613652	692848	675628	194464
81539	78929	79839	81739	80018	76468	79864	78934	78712
77764	19787	60226	132989	148167	40554	42241	26149	42435
238752	290569	263639	400985	590316	511704	556919	577658	591399
29947	30512	31197	40414	117229	108900	100249	103238	115809
39532	37862	38844	40855	70147		70929	72191	75670
				48474	48975		41966	
197001	41472	244543	234737	404793	407479	410479	127423	580958
47654	59036	54767	112059	367054	103354	105146	107593	103274
		42663	48494	81301	60078	86119	98004	50860

453235	466209	443701	540196	25467	20148	20785	27382	26558
74222		73303	78336	135378	135132	132069	141468	143515
71923	68215	62400		125120	125572	649431	581499	576551
339139		317492	330277	33982	31470	29591	704257	30890
112046	107590	94559	87308	238156	236706	251251	255010	270787
			94352	265545	246949	253661	257940	280226
				236809	357904	365903	252682	260438
334501	365540	336744	400174	616437	473730	508451	532792	573295
							60003	
							38040	42387
306358	311347		317185	1375388	1363789	1427858	1413088	1422057
228492		194024	99424	263233	13172	13248	270997	249479
				124844	119947	135568	143869	153223
					62823	34810	33878	35921
217215	122481			18971	89112	21404		10307
	119092			66306	52229		65385	63475
154402	75795			28384		37839	69846	26884
		61172	19397	209232	180012	191687	203337	239827
332125	340910	276473	199824	4092090	707124	3814736	4108845	28773
58447				177110	156538	149884	168498	197828
51342	51347		46928	73086	63389	58373	57624	67819
876840	764624	115898	57948	155674	148725	209001	73206	323513
				150565	139590	149587	181276	160706
19102				75943	72652	50931	80970	80045
16768				43585	46107	48285	49099	45613
27117	244668	236256	335159	805280	724836	807474	52837	38449
64778	62461	49424	84035	168215	28739	142563	152705	33506
57134	71636		57508	50699				32492
	84254		23069		30828	26192	60472	67492
570501	308838	45326	38441	41116	194574	298609	157727	353341
35837	41159			167147	156494	166970	173206	173492
4105108		4151436	4459196	334909	326967	316539	314748	314331
72471	71031	75645	61668					
1557394	1757228		390553	122578	128918	109717	119813	107373
462908			537282			311531	338788	107836
				46495		54766	53347	54896
				74680	71257	68289	70364	68876
			22864		31523		43274	45376
246647	106563		65580	921811	831906	143402	177496	134818
				2439260	2154260	1847274	1939539	1789541
			35315	224321	208916	209974		214664
68526	84778			283762	257928	252509	244401	297006
441688				218180		232484		
246760	164058			758728	766602	660691	705063	808936

101359	61676	43629			134816	45212		20921
				538350	355880	512705	786111	
					88085	96626	98356	
				97922	87258	92424		99814
427844	445253	331084					81196	
							34299	
48491	178908	63572	33951	823476	645784	699739		871716
				93759			94980	
72027				223422	221056	196627	208079	234041
47780	55320			126004	79432	152760	103156	134744
	235688	142537		3586162	86500	3384392	85496	76832
19440					71242			69528
557720	554746	498350	618791	1081997	1094072	197298	773567	483576
	596680	542962	550006	822855	751274	107894		97932
2121832		2107632		5571356	5542759	5144797	5792452	6332033
		38283	155868	138448			138564	
61720	44641				247592	250824		
124589	89096				313800	320510	382654	349441
24507	24797		158598	224797	148646	238405	250138	261085
				126858	111189	121751	124878	
				124338	108037	117945	123774	131706
143686	148655	129586	133943	57064	39756	39742	224365	62212
				44355	12600			45180
433109	448897	477744		1026569	994980	24111	972303	934217
59026				663421	772696	683787	712284	726937
				64796		56549	56822	56443
48868	62359	52799	134766		29377	33854	36331	
		492913	255961	1632133	1850499	1923315	1945459	1961669
123123	257030	421592	487679	189367	970177	279231	463314	185210
336564				798340	740015	739213	743134	740417
		25573		106924	128235	66410	51577	73323
251146		452378	479013	767470	789764	724261	738991	851959
		205360	108541	729500	686645	110442	26769	133950
	563784	146107	78419	530296	524015	616696	670272	568601
1712940	197044		149448	3116151	144876	2938426	3169714	3318650
		116186	127432	251928	172834	249564		225528
				201818	206283		173873	213890
161150	176082		398155			602446	616694	
175268	221940	195044		539924	424764	7220	356549	503196
70207	60287	40057	44712	67063	87757	91280	49872	
84680	43050	58624		422760	361138	381818	404202	376958
	268548	145452	34280	558396			448948	540352
38928				246795	196535	143552	191033	143606
	686908			125119	94895	310850	93504	192446

123892	182348		100728					255008
				331760	267619	506600		
17002	854183	132188	121996	1718215	1826172	127565	129838	1928730
805812	721396		662169	724992	683360	706764	691740	667724
	80906			162402	164353	172428	161570	180423
29997				106079	95843	98156		
423922				352314	280669	365488	485714	323353
65099	64960	147375	167688	257803	241135	234506	226689	254072
				66948	61805	64080	67040	56024
	35150			29616		161845		
	644952			313604	166944		141716	146824
			180460	6988	32879			26866
	9384	45642			108639	104296	116781	137242
163640	165538	165243	159758	209046	232647	190543	184501	190528
430645	373467			1315763	1270935	1241423	1197521	1330493
						65953		428632
52723	49181	52919	65024			190916		
				51105	144868	42459		41767
114992			47503	141097	123289		18656	119266
				48381	378526	382789	362727	396489
41118	296839	222833	126810	42307				50254
315451	21104		110078	440645	382334	401961	388169	440858
				167079	178740	115676	162179	109432
				79194	70438	81526	69513	74112
253545	224550		125939	313239		309692	357564	
			184192	1265925	1245903	1331010	1283854	1270066
185048	200828		42871	309236	307635	330046	43860	331097
			18668	35849	6516	49012	39073	39567
	538225	146160			123752	362322	130831	331094
370041	86656	382963	154696	149396	131309	118018	128344	116883
413091	606090	965434		113950		47780	21880	512876
74576								
				99437	100851	90152		82920
	152100				32156	39147	25489	43212
				52616				40540
				291346	246551	180548	200381	293820
			37796	60690	63308	59550	45633	
					1686062	1622884	1623210	1664596
	131820		26670		13580	101283	8816	162900
					194889	196544	204122	
209100	213740	131268	85156	200217	188020	219562	250242	238831
			27832	49272	49018	48368	36354	31455
451119			185407	639835	644807			676392

136186		120582		334067	322814	339158	340869	300442
				39458	75316	63628	66925	59556
				776833	699645	3235064	781244	2946244
542296		461451		5527979	4433051	4977858	5390310	3773424
		437342	390029				514021	
35080			30300		40016	35363	27828	
				89715	59696	98925	81212	72317
				1747330	1776548	1728717	1741426	1659504
				29792			26455	29724
				5707435	5653326	5406654	5843812	5665346
51880		43005	28520	89117	91783	89651	102422	113508
		8056		1098010	525914	585598	872929	434750
15100	1857957	3200153				247323	52085	299610
				104321	106934	111246	102652	
		179659	161806	12120	128897	141520	153112	
		221654	257236	312087	314618	336932	324228	
				284051	284496	293619	284977	286755
254524	250605	216178	85773	95780	138537	583593	60563	56797
	638155	174538		1187103	907242	1103921	1090519	937615
				1244920	595806	768889	1073337	1038023
12800		1225712		5494196	108712	41880	28684	4556969
			130552	4047338	3637144	3916373	3982159	3121191
				34128	37575		38883	38958
542297	510618	551764	459837	440741	435597	457045	461008	454733
1369529	1169997			1182534	1256971	1203801	1186636	1164653
				14968	14410	10636	17308	12484
3736044	4085516			3602336	41448	3424928	3590795	3601278
				23797	22992	26636	25539	33684
726851	667686	201081		501651	445400	394080	522072	421873
				140608	135535	145901	117421	181054
			16212		20924	15652	20724	18640
					53095	54176		45964
		122496	110184		77709	84671	90335	86760
4140	141179	172306	171621	151633		157754	355820	155858
	235612			183197		190896	191600	195151
28608	63788				39603		10133	
				874829	674033	822066	834043	789800
				7248	6832	6940		5339
					2447855	2551740	2692022	2471014
296907	291892	299227		209867	217362	214816	229822	218274
20335				77159			38939	60202
906558	716726			760093	776870	774137	753870	728168
	2241707	1183528		2170115	10872	2034412	2170003	2190166
20411	151931	654536		20276		14168	14697	31835

					12976	11360	11053	
11445	12530			6586	8621	8200	7910	7128
				144184	112706	14782	111680	11404
83972				73844	82865	74566	82291	
40372		52592				27518	28080	
				16205	15594	17369	15854	15838
					25979	28515	26359	24849
195148	129284						23613	
69907		61136		10159	12932	12411	10545	10492
241345				75930	234388	240528	74876	75202
53010	41792	33574		5894	46854	1324	39788	33940
118527	126386	117819		130171	134955	130817	125055	128471
				35296	97651			28773
91148	582156	721168		573308	429916	524847	550142	528453
26389	27998	25268		18347	12568	10132		8004
12488						14620	6615	16776
	189772	87728	11504	31068	29396	15415		
	2011140	1816908	960013	1571466	1478053	1461319	1631219	1588146
	3944							11000
				33654			36241	30999
					31400		25626	22204
	148796	141008		124233	122790	127328	136894	112229
158215	158637			98846	104092			
	42453			59892	39766	41362	37576	49781
				16780		50216	48444	43849
	559472		428472	468789	495604	477552	465081	439378
21514	21432			11256	10850		8801	7923
158737	152656		33686		22560		17732	18116
2728	1875040	17074	134360	1272667	1258503	1188092	1232268	1283819
				12980		12957		20412
		230424		156520	166228	158848	159030	158323
						11994	14483	
	11052							
					9364			9568
				13603	12349	13892		
	9858			7938	8291	8167	8065	
	24112		24324	34866	36588	33652	33550	32972
149027	174221			40425	50668	40240	143631	92279
						48974	52787	996
80465			37798	82953	88042	84872	79005	83593
416248		381444		345354		310194	343038	331921
5232				4056	4164	5284	8458	4152
49118	1198700	181256			21194	12054	59736	38913
46356		968587	777412	893674	853319	835556	945276	943935

84358	77282	311908	48714	51153	40510	150095	58484	56302
67482	55769	58611		9696	9125	10374	13924	9078
			7888	17925	18625	26043	3157	13388
	23687		30058	41293	41955	40608	37333	39813
334734	224331		350544	254213	268769	265915	271875	243894
		3280	15816		17934	15399		15690
183958	799053	672030	614452	675341	674788	656936	720937	703334
	13948	46100		8933	9985	8903		
			3364	10103	11583	11784		9182
7445	163601	22700	6025				11128	9788
	866896		936340	150226	166915	171276	161810	167688
			56415	31411	25601	33043	31364	35231
				32916	35879	33824		34786
141079	628510	462970	161766	147729	145339	117070	233387	246793
	238633	234797	219139	290326	300688	28055		103431
12969	14535			11065	9690			
89871		169024	11961		20031	19700	19005	18461
50211	51543	54455	29924	47869	48428	47763	46273	44629
				3895		16186	16416	25592
304636	308428	202630	179733	192153	155464	171955	222956	177853
29512	22809	26230	14509	19223	26318	35104	37919	29793
635797	807508	637384	432276	517557	528220	476364	526605	545094
	75560	48475						8435
1444	129453	27513	24507	110933	132334		20769	43801
25372	24117			11430		13260		10984
1964		71903	55099	26242	18575	19214	17788	13258
	318372	73008		12758	12119		14750	10888
202700	103662	435308	222144	174832	317087	11032	7836	42280
467237	381129	463731			6688			348704
		8769	67809		12065	11577		4693
			41353	21990	16207	31216	32112	18512
4020	43730	26295		16984		13475	20791	3470
59388	55437		33888	21810	43467		40496	9290
	89333	136729	26119	78564	132647			30242
3168	95337	23224	76285	70428	103384	91019	6862	5502
	32257	2984			19394	26529	25223	23990
	1390077	2064760	1713072	249582	390382	305966	138355	7760
11040	40255	99942	929983	20600	242462	331639	18524	5561
340047	349711		215997	225562	220965	256436	243915	251638
		45148	69410	22904	38837	58140	8060	
		38693	116590	28894	50114	61467	12498	8265
90369	47482			66505	70582	77827	72439	59079
	1358609	762504	245157	195959	315007	293732	243121	658727
79657	248388	1011044	706011	160810	265030	176076	120043	365561

	31195	940390	1360417	18199	68193	15336	56109	51377
1772	23219		529118	23645	90117	38484	68314	38807
12215				13145		14467	20864	20611
	3552	20700			16029	26036		
7887			110560	18407	30643	42347		
3484	67378			13280	23030	23627		54099
588		19712				19869	33416	15799
		4360		15042	14604	25767	8980	
15729	16931			12110	11899		10770	10185
78264	374867	304848	22802	130865	7815	231370	5911	313660
2640		10863						7404
17961	12440	356857	602021	6049	43366	35112	12272	121489
		99375	81045	10095	38591	14512	8659	24759
67993		16582	347057	27653	63096	44027	19970	16885
				20528	32867	22164	12643	54563
		15396	16300	24449		23927	7784	
47062	14408	16000		29951	29235	37988	31706	28935
120907	97820	149245	96501	73747	91720	82534	83024	78610
4304	65480	15908	107393	4436	7744	11310	15517	60167
	38069	14140	25422	12320	18955	15714	7533	16047
68137		16856	83454	13592	25441	58078	14356	
10036			54452		6577			
5404				17345	18780	12817		35533
		46380		6908	13472	10374		
25883	20033	2244	19498	18560	15773	17608	12440	17344
66903		49505			41903		39878	43938
14872			9215	4222		3418	3513	3409
	17000		16984	13326	13590	10698	12435	
60216	11802	72109	45057	30568	32263	34716	34550	33978
10505			6153	3501		5185	6885	4736
	8032	9596	9560	3966	9020		7315	6709
24489		25028		16111	17664	15492	18179	13520
12288		9248	11316	5604		5676	5338	
	30598	33961	19678	13451	13843	15522	13587	14579
5899	11288	13388				2703	3917	2664
	7325	8838	6393	6327	5656	6222	7030	8384
		18232	9976	5249	6174	6890	7016	
	662641				47292		34007	

L6	L7	L8	L9	L10	L11	L12	L13	L14
319498	188690	162703	213105	198604	221435	166615	174560	153279
29529								
34597	33457	35570	35214	36066	37597	37926	39815	39971
13602	13993			12472	11487	13082		12129
39473	58330		37122		47401			
474180	871796	885972	1121819	894113	1603721	1299660	1242376	1413635
55169	57611	55427		56784	58963	59045	18624	13381
54151	46660	50278	59559	50051	58572	58571	47046	57538
12687			11809		11497	12717		12634
231630	489951	29598	59298	28779	29021	634586	613324	23862
21254	147929	150579	197648	27001	247017	26770	197033	
28293	31128	27569	28762	27517	27753	32217	28817	23602
	26515	19498	23292	21556	21061		21510	
42993	31407	17068		22519	24219	18721		17607
180112	142686	137479	118586	127531	132463	139303	109255	82570
616569	466858	571475	633779	734591	706546	686147	659716	599759
70446	49911	66472	64104	28407	68335	32467	29452	23818
	378432	206551	260935	227984	293833	336448	287812	253463
141956	147295	140447	144247	152005	153427	153136	150889	147915
24097	22098	23529	45464	8426	24862	19219	18911	
452169	876608	880943	39236	894946	1604933	1288252	1260155	1421041
11762	22417	8749						27505
90042	87485	88362	82261	86910	88343	87216	82492	76887
160709	139217	133473	126930	126436	130341	134152	130761	123430
242312	185840	26041	24475	213793	23164	24091	217843	
74383	134637	75532	114448	52263	35891		74393	59675
114649	91892	85613	78598	92480	87413	99784	97892	80770
43385	40009	40317			36538	37687	36088	31742
110921	2804105		3711577	283460	4672878	285643	3780719	304638
98792	60674	60424		30991				
74013	14725	59780	57025	58301	56516	57775	14260	49518
37560	31669	28362	25898	28380	29577	29924	27698	26836
390186	444223	920638	413266	416135	620469	562750	412084	406372
855696	1195640	1221104	1613240	176769	191842	1598916	1727732	1807604
74123	71297	72049	72517	71230	70365	73193	71276	70551
44049	45903	73255	46441	49327	50590	39228	45567	44347
571935	487599	470767	436838	444963	439226	455994	430400	381840
106633	105472	98628	95755	98554	96341	102508	98793	90889
71101	69214		65567	66512	66943	64170	66429	64083
18963	48199		36920	35682	46815	43191	45619	
135244	126096	184213	355428	376206	372125	366140	369313	357162
103400	95643	217356	170473	96903	185445	206074	100636	101891
78445	72109	50598	52931	59848	69346	61184	71993	76114

23815	24454	17524	21217	25691	20756	21027	25113	22702
139696	129275		129498	133392	130428	132921	133341	114261
44259	462112	411759	377181	370351	357513	227474	336927	157293
32152	30983	649546	30119	31574	655237	33395	33618	34584
249455	226720	243752	258936	259118	262465	271898	261137	257352
265648	177849	52744	48692	185411	199024	218900	185486	167591
209223	234526	218954	140846	243912	246204	170475	244323	341533
564392	465793	445707	146201	99695	446152	446440	189078	166961
62734	58113		52688	51457	52892	53524	21889	46002
42532	36164	33578	32774	30981	33647	31651	31509	30841
1423518	1358773	1356611	1314067	1293921	1309968	1318029	1302868	1310951
	288303	233653	277806	286474	13637	8420	233357	258815
138784	124463	128009	124121	130835	133592	136334		122271
66189	32563	29703	64498	66024	65218	69611		61906
28187			13287	10756	11506			100340
40189	74552	50432	31132	90279	89233	73453	87293	37976
33678	15366	12355	16679	20667	17514	20079		117174
178040	201453	190032	199304	237476	252697	236591	230601	194631
4392079	36600	735216	2903742	3070922	3147743	3381204	3065994	1787860
152721	160770	160973	163232	171267	190074	176273	182107	144364
51153	59102	43388	49491	54765	104673	61648	61179	60725
320251	48721	687240	1074981	117475	129789	132781	130546	42005
133734	125892	144263	137583	144902	133339	147874	127233	93503
77140	74640	68156	63374	65455	70965	73710	73172	17141
44029	45362	44614	45655	44760	44282	45194	40893	62478
31780	32059	32442	31502	40855	31368	39796	34651	64455
164275	137082	132028	125797	32523	131002	132461	131705	121827
43144			46113		43913			
30812	61001		45144			58365		
63240	181887	135341	32680	88960	84031	95953	88270	177639
172775	157480	172368	175398	176159	181002	182415	184807	228932
301243	307210	294581	283320	271660	272211	911742	268213	858090
						277538		268626
118443	106034	98948	92318	130913	85969	78213	78348	80969
97696		322120	283877	314752		97060	305288	
55103		52982	50506	52079	23677	55828	53143	50223
65873	64122	65442	64506	65207	63525	65000	64149	47416
25780		28017			34260	30371		
135906	873603	132793	839753	964821	141161	1031296		252645
2206970	2909759	2801812	2346465	2561903	2729975	2879879	3013839	3174896
185942	190813			202709	207557		202518	
178313	224872	178971	220291	233260	286412	292506	252381	274563
224708		208964	207014	209788				246664
560483	670591	640815	603197	663007	713464	653771	651335	454213

			49544				63506	36606
647772	156756		915460	1030866	1135874	1115462	1119557	861625
	90705	83895	66751	77146	69252	83729		
98386	87603	84282	74857	78008	76463	82273	78683	59576
		59532	622704			75176		
38188	46650	51098	42651	47892	43301	48829		53787
	182220	180740	653244		208240	201292	729351	191696
94459			94110				95504	
176840	193246			196492	205486	194676	190460	
132149	77188	69840		86958	116901	24694	102477	136640
3059344	3274346	73133	3118748	73567	3489417	71062	3390153	3222635
62060	65586	69498			84055	82101	3240	50171
552116	376952	1005338	983009	1081569	284076	313563	1041038	507363
799943	793213	86603	759208	100416	735122	102551	758987	244889
5169293	5646999	5600351	5523495	6122451	6409595	6065520	6000401	4310412
116018			19084	117855		106356		15035
40876								
335721	326013	350945	305235	381436	396877	400624		312752
258213	138816	105345	73425	97769	84275	96455	74346	
	116549	104474	97693	96031	100915	106341	96687	83176
129895	112310	102576		96236	96944	103164	89151	84335
62515	57649	62422	69016	295507	94989	89210	98733	249953
38265			53706		60891	39756	65585	
925299	952975	938945	914761		866501	10976	893802	918404
709117	821912	711238	884644	742372	764300	780041	794886	950308
57143		53021	50028		46077		46079	8055
		25732	20757	20973			22804	4468
1873669	1585851	1755617	1939055	2214747	2354387	2330693	2331472	2160558
228497	98929	924284	116542	155636	125309	134493	174708	916902
724420	698865	756749	703655	679786	677428	700909	672945	754270
47896	57655	173956	75365	131152	108125	74858	74678	85138
628963	681328	644939	577658	656308	657051	619506	592576	
684133	573565	354424	695434	54144	746988	763663	738717	716746
568124	470787		563835	584573	592409	611973	626462	1309602
3110411	3141394	3587465	3936844	4452070	4719077	4809668	4559075	
	180498	25785			168301	204012		
148022	184906					168849	171375	
515724	549891		481375		544480		495812	
365422	470800	418220	359123		374697	411684	440072	448040
50273		25488	45319	47508	60343	82403	43982	
364595		397739		384049	402772	388985	377868	
		385432	422352	473220	531756		502356	
156697	251380	187392	197437	215375	288769	267781	210212	376560
98825	82998		53916		58649	39884		40402

447416	253184	262912		493864		276708		465184
				249713	384488			780876
1898705	1540760	1726802	1894266	2142687	2306084	2413933	2363577	27362
676740	554650	701104	661436	666520	631304	639348		677120
164820	155194	159284	167123		203047	199198	198548	197410
94069	9644		91066	86407	82398		77538	80664
		374408	431157	623937		654441		
193839	209865	211255	180208	98483	198056	78908	178740	119367
4572			56594	54923	55101		51060	51740
	155999	149451	40705	67076	50296	57245	48456	
132196		6760916		7989467	511008			972448
396745	392501	413126	388574	388298	399130	26317	26501	386198
97987	117548	120696	106372		148019		128467	123964
47181	161713	201194	75755	212774	156564	134752	183134	155582
1054666	1102441	1090845	1010569	1116294	1088839	1040773	1007944	700712
	103628	114176		11200				110384
					11888		10136	
35536	29445	40895	32767	40182	43606		47576	87508
37208					22677		128616	
		112635	110635	112839		111320	106278	111433
355801	382541	369008	372496	384112	431308	367690	391428	381964
39037	52555	56598		79333	91919	89834	87069	73227
370332	408667	371588	382358	393718	413858	395868	395400	387006
115689	118412			208912	190056	190306	229938	121459
63753	67627	67188	59950	68254	65042	64492	54320	65986
369333	352615	365420	380247	398965	419782	396566	388174	383533
1261189	1044828	1193057	1345735	1468218		1507682	1537829	1531701
308183	269505		331543		47365	427240	376144	60401
		34094	9128	8348		48240	36670	51524
5796	467138	411226	754968	279181	45753			196655
119445	134102	232997	211295	117030	247622	119540	187776	228652
50333	1270336	32876	16436	26218	17104			59261
					40932	47325	47122	36922
82906	104896	9491		102884	89149	87966	11204	
74296	202155	122270	162232	75218	10493			60378
	40968			51824	5296			
	256638	181661	199744	167644	195236	186328		170839
66054	47370	63909	66888	53363	59503	4868	60812	64214
1516522	1562623	1547694	1541982	1541325	1533029	1478190	1477273	1383069
		13056	161050	95510		73760	10087	322794
		178843	189797	192702	196030	193504		243520
243360	296398	248215	251260	276833	272226	337567	271986	302822
54043		52990	55740	41457	39756	49666	42611	54374
648048	566839		670861			773435	747923	737403

346709	76959	222760	228024		169799	87329	333232	
56895	63745		68725	62410	66784	65763	67770	79167
612376	745717	716619	821646	926647	3568137	767114	803762	18277
3783203	5021106	5098252	6372365	6711451	6524968	6571536	6383354	4446906
	572068							611472
42546		41258	40284	33370	26850	37010	29299	51000
69330	85533	96285	85646	60638	60684	52888	82216	80626
1600371	1624527	1639692	1694478	1659190	1622692	1589285	1583521	1598223
26921		35308					38596	
5359141	5705919	5492583	5768948	5879585	5886746		5815766	5846445
	96088	103126		127344	105706	97604	100965	116697
1047902	901478	476478	977196	635962	393557	444489	674947	2335664
601624	1734843	969870	762981	84329				72908
		104701	12004	109362	105809	104835	109334	
149878	139833	156094			179148		166241	
300504	292967	321906	331772	355672	353431	346278	360419	374945
268351	246488	275512	294215	314243	328741	306478	319736	335319
65236	58350	60860			57796	70252	73968	
933620	1038119	1081701	1114930	1122758	1132501	1124636	1118728	921812
1619025	1375703	761115	1390067	1219440	1325693	1338953	8764	2115940
14636	9860	5230169	5171218	16496	33198	32000	34163	40982
3279172	3106487	3801504	3567413	3975037	4143629	3768038	4141461	3845674
44584	41594	44207	24728	38307	38947		41434	1836
	72892						522624	503289
1150234	1169849	2652	1168401	1151030	1176854	1173805	1142450	1166178
	15914	14659	22796	15524	16330	14823	16599	18834
3388406	3640947	3635726	3710049	3792370	3935773	3921430	3807072	3816608
21760	32228	31716	38164	33100	34996	5844		34924
669041	547154	395738		519117	731823	1133427	1062901	1263217
	130724	41484			14079		85866	
17572	15149	4652		15433	18788		15727	19364
49406		49692	53377	53776	40912	52268		52193
85160	80559	87596	79919	91473	97205	94899	90369	57687
148264		159400	435904	169262	416228	166619	445668	160846
188813	173586	185885	196445	198063	216408	206500	201842	216142
	19851	12051	48780	29869	21987	19725	36120	
784367	811630	824204	814081	812888	814082	823512	822406	784801
8364	14382	9336	15616	11481	9389	9192		23872
2574892	2603412	2729909	2862024	2901519	2883926	2951721	2918205	2897313
225344	208496	235218	237092	258909	259861	258799	256802	243300
	131216		31564	27780	30799	29883	60933	
700707	720796	767414	785884	770568	758727	743415	747820	771242
1971266	2178548	2149096	2313980	2300504	2259949	2228216	2253563	2309630
13529	15883	53332	16298	13250				20388

		10256					11409	
6583	1876	9667	10464	8862	9906	9133		8729
13277	116482	146120	147660	114442	9996	8402		3380
88724	76816	78898	88069	79451	86064	76540	82954	132448
29036	21049		30635	35462		38520	36839	31111
	16063		15757	15719	14213	14173	13984	15451
25666		26845	26351	26930	22589	26024	28050	28605
			14660	22296	18697	24424	25092	41609
10595	14843	13849	15658	7794	9757	12373	50501	45936
249048	82756	79258	81716	82368	252217	75898	331624	76236
38757	42139	44973	51605	47283	52354	40991	7952	48134
122177	123379	124971	131882	134646	137213	131564	133988	139182
26002		26788	24084	27428	24602		26426	28160
542722	543806	541244	556789	545322	517717	539098	541120	549249
14373	30546	25655	18183	20084	21829	27017	27508	32571
10912	3343	9048	10012	6820	2060	20792	23912	7812
9518	30836	72557	79424	45953	35753		67216	58204
1662834	1625122	1614413	1703031	1752494	1681213	1712933	1703464	1736608
15304	13655	15532	5668	16680		16934	15839	7780
30593	28995	36012			35657	32961	36312	37227
30034			26259		23484		3188	33882
151977	140923	130685	143475	140597	129357		150735	187340
	98596	91516	113850	124696	141236	126778	124907	121300
23062	52556	30728	5417	25686	26411	47942	25137	62819
		4376		31026	41741	45772		47716
464598	448806	481369	480132	467870	468814	461557	472383	483086
10591	15570	4464	14828	14930	16006	15765	17733	20577
17589		7024	20428	16427	20700	16684	16984	17600
1219420	1310616	1259122	1285738	1294239	1305048	1305289	1294695	1333521
		9214			18448		19460	14672
181164	1888	190956	188168	200993	200103	205801	5363	267412
14364				17462	18225	18998	19205	15333
10288		7912		6773	8904		6331	10644
8340	9537	9246		8049	9714		8101	8701
12268		15300		12708	13336	12899	11889	13700
7527	10050	9197		9216	8422	7364	9119	736
31853	32919	32630	34326	35913	34583	33517	35711	28290
36972	38962	39641	41355	38100	40846	38724	37623	37245
51400	49808	56824	52182	57795	53463	57822	59772	82284
79515	81567	85658	83295	85229	88402	82889	3244	89646
335481	327966	329156	336451	339623	311701	327311	331658	342286
9320	7678	7102	10328	11160	4560	12101	13452	4760
10316	66893	11430	10183	10398	15719	8855	11078	19031
938781	941101	924028	938862	980945	915798	940165	937699	989919

69977	63995	83592	76183	57334	79066	52444	65699	143376
9364	9135	6897	15524	7979	45751	7403	6751	45918
15935	12392	14132	14517	21096	15792	11712		17064
	40764	39079	39768	39150	40103	39473	40667	42757
271033	254933	264832	285185	282890	267751	268987	291552	290402
17569	16673		14634			16391	14318	
710444	721805	692019	740105	732344	708823	715418	743617	777784
	7757	9690		10044		8679	9068	9241
8347		11906	2868					10307
4527	17059						3461	
			216032		189933	170947	171948	191713
32771	23604	23461		23035	17735	23157	18665	
35747	39124	39028	35345	39512	33740	34493	2428	34665
98121	134690	141967	344744	97827	252664	124293	184214	129975
82531	80822	73131	30232		23267			30114
9576		10799	10268	9998		10912	10695	10920
19787	20756	96011	99412	19974	916	114927	19184	113515
48048	44045	48653	47661	48688	49499	45480	45186	48423
15624	17328	14148	12833	13957	18376	15192		
180335	176131	192634	186583	180019	175253	183373	180834	200272
43909	25919	47165	43784	22418	36142	32181	30306	32585
517446	513371	507684	512033	528281	512100	529978	501261	563028
35306	36806	37606	26330	34918	27100	29268	36558	45348
11616	12500		12446	13151	15249	13632	14671	
12705	20558	8959	13934	14557	13777	14170		18792
8730	29748			8474	5062			2950
46561	31521	99392	154950	14220	11217	8006	6154	5531
401357	87108	7008	289894	14341	13755	15324	18907	9139
8978	10810						3028	
13812	11594	14023			3884	4515		
	6994		13204					
9875	30543	68436	55485		9308	8482	68746	62573
	19069	85776	7987	14914	13218	9735		
	10924	14696	7272	4874	3310			
25477	24177	27060	25880	25341	26408	23118	24328	
667815	566881	617291	242905	223324	239262	270557	247352	221140
254500	260504	203174	9490	11256	67817	5506	68491	58267
233017	237012	242760	247160	252711	240976	241703	228172	257118
9522	9147	6031	7537	8248				5864
25374	22593			10402	7979			
77067	64348	92872	77075	76926	79275	78958	81059	85341
496944	397565	469870	386264	591695	389169	495727	656077	754808
370603	268808	277717	138836	101968	120256	100268	113619	221150

218243	18026	100141	16123	33027	55796	43768	39025	66790
29282	14854	163558	108284	112482	43077	25536	42029	7289
4140		8801	11932	8958	7921			
8187	11358					4298		
22499	18410	13078						
33120	32258	38327	34140	43529	38450	45176	48408	49956
26571	6132		32860	41560	39704	35163	37729	33739
	20862	18677	28759	15487	16897	15955	16836	12418
14472	11850	12355	13385	12472	12085			11612
24875	14379	318419	12698	7175	244689	9134	8060	422064
8924	5978	8002		4839		6446	6814	17125
46188	90599	88416	44193	32489	20878	33482	6938	20879
34729	19641	19545	59804	68756	22987	9863	11854	10846
87810	8028		44551	20172		19602		13940
49934	38790	40325	31809	38483	25535	32547	43249	25889
	20286		24466	15057	10692	11369	15057	12892
15720	29308	37270	36117	34411	36990	36691	39707	39042
90529	78452	89247	88832	90280	92950	96907	96770	101704
53829	43171	55417	20267	28161	25264	31576	25562	28409
24390	13399	22595	13770	15941	19004	15896	17102	17042
53117	62949	44344	39931	23080	15137	23097	14127	15178
9354	18243		14802	13068	2864			
31963	26016		13395	5467			7440	
	8368	15354	14818	18414	17595	18256	2876	
2886				6032	6939	6734	6379	7759
16703	16370	16518	19355	23544	19693		22840	22776
45951	44855	45359	49407	46846	48864	49664	47301	61616
3787	5058	5349	5807	6564	5529	5283	5572	
		3843	12603	10203	16212	7840	13907	12479
38191		38355	38988	35616	38652	42432	40607	43986
		9128	8170	9343	9558	11126	11132	8713
6970	7344				7404	7308	7957	7174
19272	18153	16814	24060	23208	19386	19305	19650	
6007	6004	6069	6383		6780	5332		
16504	16632	16105	14991	15978	14588	16590	18170	19128
3605				4398		3818	5697	
7240	6971	7609	7127	7627	7523	7726	8086	6882
6025	6132	6221	6823		7029	7120		7242
61184	54708					49423	60968	

L15	L16	L17	L18	L19	L20	L21	L22	L23
183757	165817	235968	172303	158714	157781	187216	212589	
					27139			29100
40214	39626	36399	38639	37795	62095	52719	55204	57363
				13529		15406	15855	14870
	35485		43075		33186	34495	20754	
972078	1277052	1529955	843221	1139317	1145378	1847412	2225806	
56032	53575	45798	49195	49007	124149	55139		
44629	49216	50118	48430	39008	38057	38841	41535	37673
12997					31288	16446		16510
22268	37541	23325	317401	24820	19793	19886	22956	
45025	185825	209046	162218			194720	17423	18777
25748	25436	22595	24144	23730	26267	23740	23748	30275
21430		21503	20106	18407		22991		
		17460	16028		33046	18449	19149	43704
104406	110056	101646	103517	99339	134036	100578	89489	141528
514246	442957	417172	453599	475846	393789	562747	677639	
25504	56737	31842	31035	30667	43089	29458	41270	52067
199040	198864	282341	291943	322194	124838	337151	130061	
154947	136164	122369	121698	119601	213302	141794	143954	152122
15568	20303	20982	17682	18466	44067	17392	16484	35553
975971	36976	1527166	853905	1101455	1210220	1839937	2254826	
		17961				29439		33561
83430	77989	74954	79339	77628	46919	54236	54499	61787
126132	133222	131739	126603	141901	153926	160681	149436	176683
21776	21766	23752	21216	20959	27716	22730	22493	24622
36484	126992	50174	72694	38950		57875	34102	
87208	59435	75509	80932	72779	112779	95071	94330	135138
33620	34429	32187		39956	59398	42688	41898	44032
265289	341109	4043134		206165	128603		4960080	
		37510			78644	47727		100922
53880	61677	56768	52340	44809	65368	35816	34669	48193
28554	26832	26340	27412		19851	22456	23720	29263
318722	474674	552899	549531	398435	506596	563925	508592	
1477480	164073	1788552	138303	1341428	244012	1767380	204147	182493
70107	68418	69263	69485	66675	73261	50326	47158	53315
49494	44779	85278	32944	37549	127086	30031	48037	53371
412161	412699	398444	413779	377958	621356	348849	318571	432217
87333	86623	84016	90765	79628	63192	70134	69783	90232
65651	65934	65412	65146	67393	104179	68599		
47681	49433		46181	42807	41938		37317	29192
504916	346416	350085	352262	352042	350921	343060	353768	506622
159470	157315	106075	96171	90623	90127	83841	59555	181774
50837	82525	72844	47210	69769		57404	64803	48311

23423	22963	19692	18891	824916	1297447	796320	20165	22409
125646	114113	123370	121894	126015	193093	132907	137148	165200
32308	301742	290221	297477	273377	45038	597836	657993	55006
630514	29943	29174	644795	28387	675887	455266	26956	27221
275344	245672	218099	218996	210103	382404	288940	319840	385461
151637	111741	135993	43251	45363	145391	47010	148362	246621
346192	307991	189208	265019	181704		75870	184031	299766
123509	100424	165890	224812	479468	683718	521385	476135	597360
44122	45222	45447	47963		58485	47882	47014	55640
28440	29897					28817		
1327181	1291716	1307047	1317452	1235477				
233258	441640	247969	272504	410524	548544	234838	328691	248870
125145	115837	120069		75380	4104			
64408		58442	54855	49863	58214	44795	45198	
	40978	37071		35670	21735			
39077	93929	44079	76469	61399	82528	124364	158216	
18668	45982	58386			44347		22393	
274348	163143	179723	142504		138981	137043	88677	129509
2861741	713905	621678	68820	596147	774207	762454	296619	4096057
175424	110258	96365	125292	326108	177204	141266	29772	93295
62120	45477	89014	87326	40529	112908	35825	38137	38429
129643	1107576		469280	103651	51868	162756	142127	125284
90595	87043	109735	149569	97518	123889	97579	122627	258950
62759	62136	65363	63968		61357	65442	50169	48972
42205	44969	48305	41202		49759	43752	45123	
33980	40613	34363	629118	42630	934163	552844	540895	715939
130677	132745	126223	127068	112683	56654	105497	111313	137507
47708	47167	40253	59864	42046		34152		
182121	49047	143730	47216	50782	56427	122871	95136	73691
179810	175706	77491	60904	50250	225930	34460	154696	179658
177772	181540	1023176	171081	166063	167686	107450	115473	225999
853902	871294	865697	879346	797762				543171
	276826	270324	265228	255567	819919	220994	248765	237263
80989	75278	77987	69884	61409	39274	74656	53219	1742507
	305204	308036		247515	86957			212260
46279	45670		19608	41662	53242	21004		48543
		49789	52253	48913	57687		50729	54223
		39394			40920		31261	38510
128670	121050	200956	122958	17302	148116	136856	67016	133384
45753	2911133	3485059	3139902	3604121	2157888	2751529	2208537	
	206768	136668		50799				43450
90139	90812	179042	119243	72029	137786	134974	141853	108527
	186546							
675720	495727		532199	501263	631516	512951	511859	608966

95673	30195	30038	36696				42958	
	364592	280218		192612	516384	671550		124442
		69698	70762	64483	91926	70819	69880	93561
65647		65364	68797		89616	70496	70865	83398
729870	652081	714908	611454	786237	472804	607126	557427	
49391	32660		45964	61831	35755	43821		
708175	602773	159420	612520		497237	373935	405162	493912
94875	87229	99528	92175		90176			
	149355	101563	151609	147489	154700	156398	149446	181628
118216	90104	115241	63127	66086		21768	104476	
3276852	2911443	2345994	2275080	1998232	58684	1523086	1564895	1632552
63159		22553		19749	44096	69349	50024	18230
268137	891152	500703	1023280	439383	281618	239552	262124	1142882
635639	171150	163881	742013	746748	107926	600270	614881	612341
6341149	4517820	3259126	4616824	3799030	207976	4487187	4563781	5276897
	96102	99883				12878	95615	
	29608				168852	9691	100920	
341084		163496	207940	117096	320343	341223		68517
61472	59422	41703	2436		13390	75971		306927
87182	86738		85370	69574	87201	74845	72972	98671
79078	85284	79805	87248	71659	83843	70331	66237	98854
87865	268603	257805	46930	123117	154774	212005	28664	27642
	31605	26790	28115		57415	42383	63808	
878739	901758	856214	886526	838355	1138810	732281	760066	789048
769494	761663	727963	755104	765580	532816	376655	426050	465114
43530						44849		54002
								26143
2295808	1769015	1685998	1303446	1292261		1789628	1627728	1724965
117559	327999	463030	44562	37965	415813	316253	179106	913543
700827	719752	716300	648939	686955	922928			619267
99426	114352	131164	82372	175148	114388		221611	
653138	438776	319669	572036	634280	619758	509949	524078	723868
728472	696135	105912	573292	519136			702254	728751
547828	933895	875974	412270	428356	588706	587589	679826	626379
3857273	1968196		2119156	939417	2252428	3801542	394572	530191
	226524		217584	63350				
166760	133877	94468	132799				140829	138140
545336	372977		482929					
479788	339549	388152	8666	286772	439156	247296		346836
50129	56569	68386		45654	61102	65462		79817
357354	180778	176061	271768	169028	396817	361841	216019	208154
	392120		283856				435264	318929
186262	212994	366857	231582	309533	235103	278602	236153	39286
36659		867892	39037	24172			66057	78077

	59964			310700	291628	58568	186912	248748
232261			273730	418300			289058	246242
2299358	76714	1682403	1221707	1178292	1882710	1783098	81541	1759839
540742	659084	671848		633044	914692		10352	523224
182096	155469			104775	185480	153881	167169	150521
31940	80231			73120	117596		77628	69685
405771	118870	106798	140346		167205	506092	157572	41980
202282		111869	181638	200765	183993		141447	197055
51777	52149	56968	63372	58835			48740	
77529		124158		43037		50103	39199	
			112432		474664	765140		469688
	368278		381998			432698		463803
151202	55146		82587	94460	130125			170657
196956	82383	188155	194829	33465	136497	162517	164650	151125
1125787	757563	580680	990450	1064345	1030417	863110	799206	1026015
355356	283204		244660	8324		214577	267136	270916
	72160	79084		21088				
39392	372112	40820	84418	90277	36482	43144	79465	
		122196			29277	200740	193392	164742
101810	108522	101126	100337	104693	282356	161648	158736	178088
376655	361353	321529			655072	601956	518812	526420
58255	30156	33347	28240	26721	89082	55699		62181
401123	371440	33563	43367	33249	608011	587614	559088	533572
121798		33764	42632			138464	52356	
62721	59782	58323			81061	44280	46586	54452
388442	318207	299427	329503	295745	533000	417171	367560	333994
1556385	1318220	1210894	910672	858088	1331520	1228636		1204708
358125	321890	292646	220214		306112	69192	333020	298904
22309		38479	22405				36770	
221559	741637	497249	627857	421086	146168	361801	64133	
237191	796170	514606	222303	144336	1001154	126204	1664619	128785
158800	35772	71645	45227	538823		42768		
43528	36285				31645	46912	35335	
8869			96089	132112		144440		135036
101690	218882	76371	70542	123619	47592	79216	20810	3060
57396							48128	43236
178808	181910	150453	174483	188852		15068	4956	117336
	3832			59471	65729	25521	52429	55801
1740188	1358345	1322684		1439251		1264426	1174458	1311504
78597		350706	11320	9808		12952	62076	47076
237220	198977	197912	157040					198637
279730	280766	26679	259921	217096		265053	220574	270507
		25868	23725	61012	63379		48052	42657
749579	704129			28972	718556			636248

84121	79832	63478	113068	266896	144954		88317	242771
	93932		72947	75712	72878	77305	82792	69528
926562	4790583	899296	309462	1974678	1173954	889033		980638
5785108	1037587	4482078	1575055	944577	6711313	5118103	3497894	5050414
	587936			395827		692812	680628	670457
	44941	19010		48922	47846		29115	34820
83556	25013	60931	81127	166052	50044	85211	86083	109350
1601683	1403859	1548810	1502065	1462597	1944914	1420690	1355848	1351081
	31141	31121			98812			6560
6014649	4349463	5567462	4668816		5556578	4605944	4607209	4803816
193100		191592	129344	143980	126566	141369	129393	120799
500098	1186148	1865939	304812	131508	806941	805644	467224	
90838	1482719	234034	156720	769614		836975	128690	
134796		104269	90449				2456	114135
161081	130969				207086		160138	197203
374835	347538	326922	277676	247445	397237	323923	344512	329463
324813	303039	276450	285132	203898	284713	258398	294792	276551
58077	16362	57140	26369	20362	81248	62872	48720	48004
1123893	145927	920093	328800	183529	1636239	1037315	699733	1015862
1116296	893474	903631	639249	641580	915760	1002696	983688	
32695	4845488	5047963	4601359	167948	4015250	4735217	152664	11700
4408860	518544	3174838	1033852	516480	4950418	17400	2088272	3146701
39505	50226	60312	56007	51981	71280	65472	71723	59833
644951	545512	465274		428906				599766
1095429	4660	1142645	1010039	967811	1451043	999014	990782	894461
15388	18421	15948	20584	62907	26609	20715	26468	8199
78628	2603646	3531352	2870718	2533360	3712522	2971403	2917984	2826090
		27188	20964	22644	5576			24126
712604	777038		348304		474816	395944	366820	
	227166		106941	83268		18922	152490	19122
15352			22060		47820	1708	15205	15920
59881	55263		47184	44906	78660	57828	60067	60460
90509	67643	46965	85172	71072	110674	105593		124001
167261	170415	159099	309732	132741				161509
210020	206284	189552	179435	151493	196643	170655	179178	177447
101433	41596	45532	26453			39792	37509	
830754	168733	803546	531009	345155	1272513	732363	497389	721849
8604	20587	8360	5488	26136	9312	26095	5547	7536
2978987	486471	2743526	1596039	947212	3567014	2049888	1425476	1968657
302620	235293	10410	206892	210785	364728	281668	248885	
34395	39076	42672	33027	30632				21849
709085	602086	749652	659907	650754	974458	646143	636994	569467
2389644	1572802	2168506	1774383	1613073	2247919	1707731	195044	1632771
14503	92994	13411	48650					

				1632	16108	8136	9228	8100
	7919	8254		9407		9695		7784
	117241	121403	118011	113283			7525	
	99351	143659	111623	81447	107420	115577	101421	95035
	29732	37872	29857			35020		
		14679	20732			20331	21788	22620
30119	26669	27446	24926		41236	29088		
29454		14252			16496		13821	
14009	15535	20748	12262	10285	16544	30536	19035	10948
76865	80948	266680	70349	71666	267052	214252	81175	76096
46019	40630	44513	43894	46485	21212	42408	49455	
134931	141021	131507	123154	106831	134732	107455	112891	112893
28760	10940					18448	17217	18375
559636	233492	559416	527871	487213	853415	507055	280878	443152
33898	28210	47564	33997	48077	23960	27276	22294	
32459			9544	13828	21220	29174		11471
237132	102288	80387	40596	44205	37220	44183	82622	
1771014	890324	1697623	1511015	1262275	2173212	1304955	716808	1159399
19212	15116	17396			24082			
		37171			73383		42075	43042
58812	31011	42819	46148		37860	42200	34065	27468
187697		211120	158149	150973	179752	193339	202982	172935
145157	117380	121426	102332	117724	209024	134434	119735	121109
29611	55382	25577	55924	21176	30604	20452	21484	39761
39195			46640	5668			19212	22761
401935	367982	437631	413773	442225	658506	403107	405401	358753
20076	7704	24393	17929	26165		15186	11887	
18716		20500					8040	
1358825	905007	1260556	1083782	1053858	1423213	972725	991219	913385
			11920		3624	11176	10916	11355
4930		149927	101988	181552		414308	427936	394399
	16613	17856	15567	15820			13094	15418
9392		11520		9259		10236		8252
8345	2160	8402	9502		13827	13706		10558
	13455	13948		10125	21805			14296
		9364	8268	9497	3562	6634	6998	
32008	26855		30328	21671	40491	45096	46052	52219
41514	43083	40786	37515	36349	56295	38476	41525	116498
57732	55988	67316	53392	49971	92528	92060	86216	71512
84731	88239	83702	83663	68208	93188	67161	69048	67120
326628	296690	342285	357997	342480	565698	322006	149604	264382
			6520	6232		3500		1440
206629	7580	211395	181491	12712	24350	172127	43377	26010
965029	841178	951674	966628	864174		804211	341539	633799

61027	187277	56449	68861		85063	101714	100942	64453
53558	44609	5184	5868		15516	48849	44752	12314
21508	20916	18496	23527	13440		12784		68942
	40318	39312				46754		55647
1152	239062	249799	247255	286292	395865	223973		217895
						3280	21087	
827012	574175	751820	655797	681807	835621	516970	558135	525195
12996	9141	10271	12432	10143	21911	16624	13545	11515
12133	10362	11288	13848		24105	13563	14155	14894
60754		56596	46741		12916	137484	28673	17686
	218288	219644		220076	297500	83084	89155	112644
10695	15389		13644					39335
34656			39516	33776			21695	22385
267009	109408	253686	105516	109976	151183	224486	211678	96467
47092	176988	29942	166978	117987		9440	89421	
9246		10230	8819		15239			13025
19084		141873	91635		96518	63053	57322	65685
7168	50676	50317	58544	41661	54331	12124	38286	40067
18064	12453	12356	12992			12652		10760
180621	189542	185221	189447	184970	318122	180450	11525	137912
21604	47132	40763	38177	27715	21322	31522	23582	
522856	513263	525293	520386	494161	721810	445081	216350	333021
66609	32223	30975	22824	22082	11908	116900	62796	50510
52107	79235	24846	48824	33862	15369	38895	18673	18123
15667		15255	11994		22612	16329	14168	12321
30200	55196	16646	26675	46831	13019	9858	31423	14154
41061		32880	43861		1364	40997	11483	
8616	170638	126899	115430	91036	181971	18718	83818	25741
11819	342625	13376	12317	13196	12147	9622	302846	274522
		69380		40781	22276	6737		16184
	23974	1868	11557	20227	17524	8014	15711	11565
11026		8166	10069			29752	11183	
10952	36892	83198	61764	1116		34869	39680	36828
22040	4882		83143	45379	25340	16472	100473	
48321	48536	19504	31346	36443	11807	54276	37850	
25563					31754	20387	27480	20711
334298	1128218	220088	1100729	999237	13574	576301	1007077	708720
92731	804	725204	15357	359642	691703	9441	65568	262805
248908	2248		245059	224805		213953	147473	
	51837	56746	19868	38974	14698		29601	11770
	18925	63354	9911		9946	11117	8816	39139
47633	78052	58900	63542	74477	118483	62678	65533	57159
236885	22145	89422	11060	695721	368522	348743	446078	383304
246053	573393	1046047	665914	581526	801027	286428	524313	200722

62328	134027	52037	57156	402797	67190	97467	358512	123483
22170	17500	70956	304267	137670	50829	266114	16787	222891
	4259	17824		12673		21810		
	21910	29310		18121				33985
		61613	13947	71417	45421	11936		32217
61421	73566	29485	53271	44390	23922			22425
28168	4850			36376	29468			6572
6577		3760		20580	47672	11342		
	864	12918	12824		14978	10853	9157	9271
385639	115790	49776	325447	8603	89968	50824	192847	231849
11746	8840	1620		4128	10748	10740	9801	
51174	436346	53502	71008	352893	64040	56259	284489	261309
16149	50141	55557	50457	38593	93313	18724		198107
57832	3643		25212	53336	22046			
43281	68478	24029	65162	64355	28181	44760	81012	54793
		52659	18737	27786	65927	17064		5208
17732		18516	29584	45644	65620	37144	35160	26249
112736	95699	95535	94137	87924	137848	69029	80452	56534
51708	141925	26577	117995	116084	15276	76277	170607	102665
12247	32565		27138	15791	15100	18703	22770	8672
25319	7393	209139	5906	10889	179867	28193	14066	87711
4451		61971	43821	24989	42167	57435		20068
18890	45536	8774	48465			24714	37403	25885
		24654		18772	15204			
	3711	13623		6499			4159	
	20416	17277	22876	17096	39136		17951	12411
46141	44368	45796			70070	49928	42547	33224
5640	5618	6664	5091		13848	7811	8024	4609
	26262	8511	20345	21752	19491	15208	30622	8828
48652	51440	44160	46088	37640	50168	35380	27680	25064
15213	7665	12424	11461	7112			3740	
7480	9200	2923	7974		7936	6129	7817	6936
19266	22248		18259	18820	29950	17522		13552
	6676	5093	4658	6005			4834	5320
11125	17872	19690	15981	15342	18891	12697	12652	10428
10868	4088	7385	5643	3322				
7520		5795			12024	6575	7984	
	7192	8549		7996			5341	
		142259	31815		84896	62132		

L24	L25	L26	L27	L28	L29	L30	L31	L32
	211223	132375	195346		387865		229304	167996
33443				31419	24803	29457		
55593	56916	57318	57994	57070	52288	50606	55176	55808
15488	13526	15720	14008	17484	15107	15618		15000
			35202				60148	
	2499821	1903693	1563114		2293493		4866410	1597266
	44469	49721	35720		62736		49885	
38367	41902	41749	35759		34252	34649	37444	39311
17896	16567	16885		17313	16594	15870	17117	17099
	21239	727617	23428		82087		22311	359099
21094	23027		196827		144888		326660	
35883	25498	484169	24405	33237	28952	22190	163021	20634
			23322					
48915	20960			44512	37466	44418		17638
153411	94585	99110	97707	144950	138173	146608	85958	76886
	670635	675705	685221		31539		788557	589475
51978	44136	36872	44220	49702	52728	52782	38284	31969
	328277	350875	312028		741059		398171	240174
157500	156754	153842	148326	139810	120343	108074	129832	134953
23287	17180	29332	28914	19978	24859	19104	24884	31545
	2539072	1922564	1586401		2562325		4870604	1609635
28325		29565	29076	33887	31731	37450	8905	
69603	64543	59630	58787	71409	69219	70805	59604	53631
190512	141436	148097	147654	168344	142492	164367	134203	126638
25429	21386	22728	187246	24628	209490	25255	218830	19795
	38415	38759	27642		35396		44596	
152181	102543	101562	92004	143799	29043	149312	86959	27589
46243	32569	32884	33312	50411		40019	30352	
	311451	3894869	3858052		2951580		6085756	3389605
112781	32416	38048	39719	101884	84684	99036		
48890	37218	39476	37384	48741	42984	45932	36004	34933
33157	25460		23288	32435	28792	32707	24044	22112
	522268	724533	430960				776368	453188
198510	2590228	1743292	1726708	188466		111858	3009436	1429709
56328	49871	48225	50312	54004	51785	53345		48652
51710	56513	46821	37385	225374	159526	36815	32619	68661
472477	332240	337053	336215	433210	379897	414869	301468	301597
98196	77130	69308	72200	93447	85594	92932	71779	73380
86034		74950	70933	81317	76899	78153	73456	70720
37391		37882	35639	29907		32053	34305	
122851	360031	332887	341973	496386	138518	482706	354579	333995
67031	79463	70174	60060	63903	138539	56855	74081	64105
46702	65495	67724	68345	54174	58436	85692	69717	53396

19047	23994	19226	18743	21496	877628	935859	16345	751385
163048	143505	142329	138401	160744	147854	152966	137729	128960
731359	764445	775039	753600	673062	644679	48781	720714	711140
501327	28017	493968	25789	506021	464848	466009	24637	463205
398462	351103	326294	320946	352630	256869	283195	289120	298717
279962	152779	148349	138163	251245	203134	269670	43682	37995
300553	244581	173968	161797	299732	279569	308979	178188	263857
642886	472240	441546	141993	536875	148520	569062	400223	193470
63927	46981	45307		55183		53913	42129	40604
40309	29943	28968		34841	29304	35623	26858	
							717283	719197
258804	255834	260828	273170	234341	222496	242439	243553	
			71547	75122				
		47518	44291	53715	33992	36776	35805	46049
19692	12538	179407	60578				14932	203372
	54008	49207	78330		43459		46004	41547
	27738	245814	106059				21573	158179
132469	150876		168148	149784	90844		63616	64701
4460403	833126	821356	784008	95814	603227	598235	684606	747875
119225	159073	98320	116782	126715	387454	76350	113300	85600
78305	47290	46211	46855	54012	47450	63258	77072	34744
139214	158537	159957	158777	129933	97903	73471	96558	128526
277474	116204	85769	85286	208733	89360	112037	114402	71607
39653	57741	40996	41149	38905	72668	83191	48609	
41971	42536	57992	52898	42725	43821	42152	46130	54755
25306	549535	63074	555383	708418	634020	27570	514530	480816
155450	111919	37422	112391	34216	26096	132070	31222	90831
31162	29829			31398		30443	25728	
59133			210878	200306		42758		
172231	145584	148387	134529	154009	82265	89787	136671	521951
141732	122903	224328	229307	141829	135055	105277	155772	910626
				546165	503182	523637	501526	477706
237932	527202	236060	217171		198194	193307	203091	134982
1936855	52115	92155	88327	1905755	45133	802883	45614	79283
		121138	160224				146392	
56019	45118			51639	40685	47500	42574	39168
54172	50521	40219	29726			39804	48726	40346
42630	32098		33205			39707		
139311	123442	7564	118875	131573	530143	117266	111622	107379
	2570248	2812079	2496705		1993462		2340573	2280923
			40442		38454			33257
147760	181438	195490	56968		71183	39059	69559	134694
		150488						
682163	584207	259940	283603	535049	340023	414648	424968	244832

		50745	22068	64618				66951
118311	622474	781631	726517		109933	260302	626383	625683
95954	70930		59221		82200	85784		56116
90961		68508	65572	85084	81298	85690	58604	56573
	621691	612798	590036		519633			561445
	42458	42036					35322	
511087	497491	175268	440487	424858		100648	394185	146876
84098	77054						64443	75145
203731	170830			161833	102321	126055	124659	68989
	107501	147412	277335		25577		102231	115489
1813856	1807990	1799210	1726130	1426870	1042617	863482	1213357	1448181
29384	56927	42653	53653	40572		71773	48148	43630
959290	254769	459663	447606	1171652	281397	1182971	214112	237658
592405	114255	540394	492961	91159	418054	482065		45578
6077452	5460534	2596983	3369636	4603506	2833961	3669115	4090122	2482758
106990		68960	93464	106666		7740		80943
		56003						70954
79129	284833		271643			199331	235833	228347
14853	52340	52368		306420	228686	309955		
107373	71746	71004	68739	105463	91655	98544	63321	56854
106920	75232	69063	8676	102245	94956	97411	61762	53085
271019	113500	24640	69361	86352	257722	29688	61972	224817
	63519	26752	63830				55346	18164
764636	930032	30299	739792	704664	669009	605337	676076	695179
525206	481921	471949	465099	492070	468236	408803	463175	476104
56318		56059			59361		3212	
26020	9945	6052					11330	
1765543	2147079	2073311	2109751	1601785	1312304	107980	1115692	1604525
146210	191289	1902026	844198	233862	29311	243408	139548	1297882
579895	582908	659115	675753	377589	504783	478742	372399	584175
	52362	110649	97271		46729			102272
729329	558899	184697	322000	629342	483123	599997	438550	
779253	757152	741338	687244	681817	533954	453081	595161	659554
604239	672697	915772	947737	448072	455735	298885	464745	830926
647274	2850768		3621440	1367974	625119	2089731	2571974	
205996				197784	197556	135156	203008	225208
173067			130080		83388			
601881				515883	404615	501738		
365392	382768	392508	433752	386024				302840
57002		48241	66605	44791	74758	45556	57683	
295208	319406	169825	201722	243652		377368	320400	168784
414659	541252	588268	613880	354900		150900		
	180271	252907	310849		142041		323806	295558
80219		64043				75364	65780	

	555492	169704	516752	176444		285131	470224	
421340	436764	332150						
1680322	2186609	94289	2130927	1548057	92993	901286	190740	150922
531556	286317	541900	533404			347908	477756	489332
161107	202286	200904	188746	161580	119719	72119	110391	179637
	60814	54760	57456	59422	69339	64574	63635	
113935		434220	538903	134456		414187		379531
65328	156714	139075	105580	185791	165569	180307	140317	127910
41511				32864		42527	39660	
	111929	102412	99974	19256	63312	40049		
495482	173844		673716		1551116	130244	142452	
438400	446675	443523	439678	421976	422339	443211		460396
174071			131504	81324		115655		
153552	161394	91275	94892	107567	159667	90723	95911	142044
1019330	934333	334685	599675	1005459	890637	919290	821941	
171704			155320					
10136					134976	112343	75314	41942
	41549	55192	289096	57052	79654		27947	32341
169752	30592			150273		130386	178852	
164447	155156	155884	164814	150925	127194	29136	142740	
528066	554619	502588	587864	467331		354940	536876	
80189					22559	43804	42827	
586499	588623	575339	639230	518265	12736	324252	456541	487486
	103696	105542	131527			103649	69284	99084
		50647	48050	83588	183016	180540	45708	23892
311465	464313	377213	442379	405865	314439	346065	428597	366709
1171099	1529437	1476383	1451537		843826	590978	828367	1319086
317259	80608	45194	30898	269554	225702	140076	225616	50124
	35535	41696		32464	34879	8600	36992	
	181339				43660		17970	105598
123614	618633	479813	475695	122944	789841	122112	793361	545546
	11035	51982					11512	
52717	53542	34559	51997	50405	54325	47713	33737	33604
112527				117453	85891	7688		147160
			19244					39640
53944				64440		28044		
120949			155816	134699		107862		
3908	63060	48649	18027		68799	60180	65432	53210
1231971		912856	1141920			1284157	1265287	1013351
	89984	251975	7156		70783	36162		441796
	251420			187572	155348	98055		
286202	408810			288272	214250	250555	282306	
	53414	38508			70260	53828	58774	51328
	764464	760514		598875		323511	465325	723872

62525	115848	257163	251138	225060	85061	218272	158158	102569
68820	70683	76357	104087	93162	55514		72184	76683
1005981	1408	33855	6133016	272734	289759	1017579	3455560	
5668904	5984745	3514904	3689879	1173600	1219459	6297762	5646423	3732263
684524		684672		630965		544618		
37462					50160	38989	46404	42478
25889	81564	72596	158792	84002	79341	51056	79302	75865
1289313	1286577	1234094	1191504	1211598	1235403	1377232	1418533	1288631
5868	35462		45004	26365		5399		32115
4945456	4950682	4450246	4670262	3691543	3762737	4454863		4616956
115896	159780		177683	151372	145240	118791	129235	
8136	268158	3606740	2281377		94428		559375	3565101
	47428	10875						37803
		113808	123705	108440	86625	55722		113060
212144				198476	151123	185165		
	376502	361371	353855	308116			259043	355268
279035	310927	300001	299421	253685	203396	141674	219327	305568
40829					19322	46156		
1008320	1068109	699831	836268	143204		1014919	1035451	777024
17305	9752	2290097	1458555		6248		842182	1837266
	4179392	4437669	4294531		47940	12612	69983	78444
3307864	3973176	3783396	2916896	390154	960844	3469558	3690777	3007576
55554	55628	64992	67783	75632	63775	62877	71308	79524
600232		579670	719110		552994	494017	532415	
880030	916626	911103	942159	763529	831786	939804	995892	939053
13100	12108	21629	24044		26791	9744	10964	88350
2878974	3070117	2931261	3006880	2128460	2255384	2710230	2913095	2991532
23240		22680	27540	22236	18098		20591	
	836509	1378955	810283		168398		53968	
21024	73456			17902	136217			426640
12018	16087		23620					15952
57111	58621	57822	65433	58104	44733	29600	48590	57217
130497	114269	61145	80085	114357	95675	112872	111621	31740
166101	173968	162929	164437	154336	151716	86269		312048
177305	189423	191097	188114	167632	134821	95295	155989	191019
	26557	42845	37972					
664432	775267	688438	718601	82970	492422	696538	742782	677260
	6665	7644	5480				12177	10668
1939398	2483541	2490795	2493051	206557	1412034	2071965		2328717
270036	306716	276850	355587		251208		238621	263449
19615	29856	24644			46844	31812	22067	
553727	592760	624090	571515	465642	554259	598177	654841	613390
1650464	1760783	1780075	1767171	1207757	1402872	1587008	1746002	1740021
		17308					15488	

	11724	11520	3124		7948		10624	
	11050	7660	10360				10723	10548
	5956		129723			139924	152712	
89318	98768	126752		175886		79195	88888	131388
		32508	56397			19957		
18765	21569			23063	21578	24298	23307	
	27654		34223		21844	16022		
	22220	105995	36018			14472	28040	117374
11474	16883	38919	49948		13502	11254	64151	24232
77826	75704	80886	81551	78144	140604	47195	70096	210656
	50774	45208	48182		47203		1000	48332
111465	113929	113886	113278	111039	94258	70178	108767	117495
17741			26492		14461	19996		20608
399939	487983	523856	505438	150080	455352	434953	478465	471418
	22248	24268	19801		12953		18539	18142
	20061	19513	50944	23145	9166		6440	8072
18528	54182	82932	67390		11278		16759	51037
1065431	1480220	1544100	1498705	371573	1219547	1199282	1369978	12796
	17468	15515			1732			
		40397	26947	33535		33278		
27926	31203	29524		59620	2504	28448	34184	
155561	182911	265822	361044	171590	176276	152242	190977	287980
123959	141866	134278	156893	129897		96524	111785	
21908	67839	41532	64878	39345	48792	19602	18546	24749
19309		34287	21044				20284	31982
344609	366529	400132	326111	257190	357274	351459	401442	396168
	13894	16208	13792		9519			13453
11468	10452		6344		7270		8212	
898753	992102	1044590	1025494	660898	859159	892314	999371	1048138
	10896	3764				9530	16108	
	410410	491628	427448	163410	240531	241371	2320	459094
14421	15224		26958	23261				
5666		7324			9308	7880	6415	
10708		10645	11064	12348	12862	13179	14411	
16680	13515	13512	17035	13705	1504		12722	14685
	7608							
39766	40864	28760	32956	38281	39233	43344	39890	31249
39237	38969	45564	44848	38113	94676	24397	37876	42382
67421	71691	91566	93980	63454	64332	58025		100412
69234	71621	69463	67630	65762	63623	46289	67326	74393
228551	286476		1852	203806	287082	258769	294132	300219
	12493	5580	22613	9764				
5234		23611	533040	206223		8491		17025
547808	833675	838197	844981		742545	667231	773209	815837

58829	65165	59746	153502	58545	89120	59027	58585	102553
11328		48515	10544	11987	10392	10557	41930	54524
42962	43088			29965		37451	57377	41595
49288				43424	1512	51091		
1136	211310	233310	169305	146961	1836	198680	239943	223908
18792		19586	21163			2716	2516	
508697	537016	601404	573045	374722	530935	496804	579846	581418
10134	67040	14851		11981	11706	10318		13740
13947		16394		15062	18968	14023	13800	17744
			144672	49406				
90688	130576	1528		106340	82256	72199	82602	81244
40061	10205		16181	41980	38584	38953		13190
	22468	28108	23684	21455		25493	21103	
132402	215669	287706	710474	108349	117016	85477	102338	133404
57213			51716	180570	149147		91229	
12296	12376	13712	9562			14883	15039	15431
61386	76000	78161	113953	58696	18645	46686	40488	19632
38328	39540	39122	13706	39115	37269	28484	41882	42181
	11621	16360	13106	6188	7559	8459		
120541	156133	159987	174725	28633		142984	162724	163467
	26230	21007	28043	16620	14239	15535	21112	23344
299191	407267	468650	485529	377701	421767	409593	502617	526533
	1468		153248	36221	26390			
19361	1032		60027	40680	37500	5622	67046	
13418		14797		12667			15024	17396
13813	10550	12597	16641	94238	40763	12224	6836	15728
		7175	64062	34022				
23103	120420	126026	11276	142347	79410	126580	20204	115082
200920	20841	339287	10367	13227	14301		12884	10519
			5305	9362	77696	44030		
				54236	23184	46581	9599	1888
			16811	9200		7794		6073
10161	42318	41854	10389	35073			36864	11552
16172	3821		20476	71944	35272		24266	
	16455		51469	14584	28709	22439		10008
21300	19811	20620	19441			15655	24525	21469
809976		7375	480530	1444927	1100857	20988	607675	38094
163611	286359	7034	87911	76645	433798	885213	45971	238614
151278		235700	231537	177771	203041		207154	
10127				47918	39629	30446	11149	8146
13386	13268	8549		18873	10021	75811		11879
56267	62611	64392	38604	39304	63048	54777	68727	63728
450388	13500	13840	130169	644529	538294		702982	13101
203782	8679	10654	239243	574063	617238	545087	285662	124149

104151	10261		16521	157910	412651	197108	25625	
108844	96835			26828	252199	454485	524749	
	13449			11166			48295	42804
13339	13347				11404	43566		5326
13124	12621		9275		89725	79366	12804	20497
24055			92921	32630	33750		58584	
5376	25576	21254					15223	18090
	17737		6000		16377	44486		15036
9400	9220	8656	9603	10085		7353	11391	9911
215630	38182	5052	522348	4275	63572		9850	52600
8758			23978					
102164			54736	444467	274786	69269	36559	14636
124074	306555	7166	15818	60893	32427	23831	9638	
		9469			41515	46854		
64580			47179	77502	56479	43923	23340	
	20877				27349	54321	19750	
26018	30446	31337	14975	19636	30012	24799	14351	29360
57229	70825	83954	81798	3428	67266	58426	77622	79309
133115			53108	173481	118860		19986	
12967			14037	14631	12972		14124	
10593	5562			15890	11868	16190		76864
	5857	9724	596	5598	42409		49291	
23400		2988	2797		38633		20867	
	15360	11585	19173		8751	7584		9606
6569	4572		6935		7032	13475	6771	7457
12894		16160	21308	13924	14468		17253	16394
34629	42135	47702	44755		33482	33049	42136	47304
4701	9735	9268	3336		8096	5100	10748	10159
9133	14232			28055		10591	15080	13429
25094		34970	38406	33325	32224	26155	32511	34375
4539	6470	6361	9613	9671		3622		5122
3615	7494	5510	8192				8610	6366
14167	17716	16395	18622		16816	15132	22692	19592
4473	6812	5552			4964	5840	8032	
10512	11807		17412	14671	11893	10253	13437	14043
		488	4513	5872				
6054		4732	8888	6932		5372	6997	6101
		5626	7048	7672		4748	5204	6061
		256521	173064					221283

L33	L34	L35	L36	L37	L38	L39	L40	L41
266720	218148	222613			252697	121766	157228	349578
			28420	29614				16292
55968	52611	50144	52026	52195	54620	55061	55487	54023
14220	14006	12689	16174	14422	14118			
						23732	31685	24723
1869905	1422342	1505602			2458469	300251	444882	825312
42106	51626	52141				50601	47209	52513
26251	30420	28352	30731		40503	35488	30690	38967
16970	16771	16211	15236		19315	17676	18290	18243
22957	367046	370091				151931	211109	17111
152786	200186	192598			218513	65709	81365	105116
20076	22070	15863	22947	31374	19699	22220	425810	21408
								77016
16910			44059	45874		23554	22210	25284
109256	83572	81955	139550	146043	84576	107173	101027	108438
728375	546246	528325			700720	648626	622603	20554
41550	42440	36804	47820	48054	53735	36649	35471	31775
342789	254754	279517			315987	208816	255360	416294
138235		107173	108473	113940	123738	138177	130514	128747
19230	25660	24793	28208	20382	12316	29227	26990	28952
1920098	1437665	1524819			2489487	316298	382344	841371
30796		26041	33811	33781			22513	
61196	58194	60315	68235	70000	53659	57624	57487	59779
147815	142551	136698	168095	179362	128561	118186	113804	116129
201421	180066	21803		24241	21058	244488	221126	230159
37960		32156				26165		25274
104087	95765	72995	137792	137735	77320	86456	68602	79344
16497			40469	40621	27927	30919	27961	36410
2859690					4311271		1747525	
			95509	95840		55925	52007	58148
38344	38617	36011	46857	50064	33531	39876	31818	29517
24656	25718	26570	31426	30016	20905	23175	23307	23251
818460	480537	493166			502487	863349	435164	678678
1473336	1829036	1749068	116370	127712	2067232	210418	704532	960596
48726	47972	47377	49665	53974	48484	46654	45410	45054
43437	40909	33208	212777	39954	31722	43684	30430	91261
359013	335998	314446	409412	433448	284448	308689	269333	284127
80619	82838	84902	98881	97107	72421	74519	72445	69686
			74583	74609	66861		60443	66832
36285	32760	30508	31251	33329	30931			
491795	335493	333352	117486	466160	329079	337480	329119	331960
59197	61286	82608	55625	58450	54719	98345	52071	50966
	59754	78417	47321	48677	62800	50060	50039	51314

22947	806608	18279	18125	16242	750268	22005	19559	21081
140869	129923	125174	148951	152158	131421	127222	120045	129139
734087	626601	632049	47906	697410	756070	781613	775437	847400
470276	460910	435333	451390	464948	429047	454043	410843	408480
320853	262447	248448	283872	305352	287343	328983	298011	293950
156641	135590	43586	247744	264656	38474	119921	87177	81172
259644	189089	189409	215454	302464	143436	280514	180368	190990
492041	470630	458273	551076	612674	74115	94197	88399	123413
47987	48154	43859	52594		40262	41443		
	28085	28711	34068		24680			25509
743344	734631	714715	738467	754396	688640	677831	659457	668280
224020	258180	238476		256007	234207	253657	267455	213334
107044	115502	67242	72536					
	39813	35036		38689	43248	45675	40053	
16709	46111				10592	15234	12609	
30236		32422			43657	53875	33451	
14234	24390				35269	55979	18953	
75825	26063	79491	30165	76202	30558	142066	131758	119338
63748	63377	62744	63982	57929	719031	747489	729751	704633
111505	61280	59288	76812	83411	91092	116912	99299	112230
36688	76385	25524	78169	93654	71218	43442	40231	34998
138539	111261	83152	79494	88625	30850	143026	130759	126824
108509	70134	47827	119315	110334	47988	119496	122393	119133
65451	56020	40737	70904	49313	37776	48885		54540
42903	53753	41861		41647		46994	44265	43421
594053	98116	43474	26034	26961	38659	39283	28291	476579
117155	103563	104558	130855	29346	98344	90080		85521
	21946					23108		
141921		143328		51613		45044		23564
38802	107164	132655	147274	153875	95486	52020	127035	136080
128210	154719	121463	111716	125391	123225	122924	25951	153252
503186	517510	182507	185115	185362	197834	190469	188196	181732
194539								
60778	40784	63627	899453	74228	59680	57774	55842	45319
163948	184128	177456	186424	197848		169448	159588	
40198	41125		43919		25892			
			41350					
25693			35510	22595		26504	23027	
114653	103024	111808	121908	454779	104411	189092	762426	100198
2046450	2525480	2807213			2632009	2310078	2428256	2181257
	77077			75592	33466	33022	113960	
150693	130244	50133		64215	124582	207818	185261	160818
111162		106908	112068				99406	
349371	197228	234816	347396	458914	357739	378300	369260	456561

122525	43821	73538	25596		10058	23646	35584	
621056				190207	757600	790166	499177	563646
74413		61306	77781	83515	53400	51516	11892	53870
69753	65145		79970	84109			51532	55220
28677		43317			43168			
154244	102792	302795	74264	330647	387478	431698	157616	156752
79070		79484	75556		77123	75155		
136313		89207	106002	137350	107291	122125		137430
	99377	31816			32670	115882	127345	
1443485	1188466	942436	887676	1052084	1232974	1565170	1402863	1300617
56481		49581		69438		72702	2852	53853
227558	938746	931465	916170	906511	953558	953385	232117	373237
99930		58883	464237	470017	89716	405744	96550	454209
4219195	1724200	2571797	3022789	3992110	3382003	4006864	3652456	4333781
68508	70363		74241	99974			74406	
	37386	109196					64640	
239798	106139	150181		206193		292352	181984	227468
79755			290797	316381		120815	96365	127105
81207	72098	67011	95117	95508	53964	67597	49807	57077
82250	66281	68881	94497	97078	54640	58884	50003	55755
72563	220825	216520	33008	32284	53119	15460	221320	110861
27755	74474	59376			18821			45556
655470	649805	592581	596893	604144	643399	15068	603829	604041
498738	517024	516428	420256	478773	477551	506824	491610	612584
	53020	55054			5128			
20730			25880	21654	12440			
1576614	1056610	932382	699403	1039278	959271		1913613	
230646	156822	465851	245320	247769	284777	415707	117677	792743
485082	503158	345566		464251	466840	424560	461357	
		73280			54231		88090	64222
496811	182496	418210	450347	594987	382059	391629	391005	474325
352048	570575	466945	492512	541689	612944	689075	678167	624779
530862	624256	305162	292594	352737	454506	571150	524424	525006
2643456	1031724	1413299	769582	2048697	2354674	2734770	1945854	2519374
51862	165815	30996	161862		169471		57342	174156
124855		72392	95268	88922			102658	
		362116	386872	508401			331640	412676
		243032		254020	242001	331584		43530
39677	59390	57719	44066	40371	53532	55009	76574	
264361	96284	262896		297326	222720	271220	186970	256001
461280	12648	214656	148648	197998	56696	491344	427712	
172824	363769	352280	39015	21023	267720	121368	93773	148032
70110	98759	65669	70940	77059	64628	59920	57192	65108

248736		254275	288780	256880	334559	501580	392139	
260488				390188	326284	450656		
1788194	250489	248900	692299	82036	240934	1962707	1927727	197548
		357366			371474	444332		
159940	108825	63346	62833	88990	108654	181448	179947	159571
55898	56220	62602	64362	65162	60086			
290435		245585	55034	347555	742971	787563		298503
160784	63256		134165			135055	119007	134474
		41389		42455	39219	36772	32464	
	72182	38734			37233	54157	80652	28460
140540	22011		104196	135212		937890	299226	
445418	416437				415648	406137	382397	402079
93105		77834		116826		133239	150258	128648
150492	153042	38355	99565	124138	102814	154633	138009	146012
892627	367740	816844	766487	987290	737973	738387	737484	842557
148028				156672	223604	5964	380372	336992
		97941	108361	34597		29788		
73495	79938	74643			43347	64407	69768	75774
177212		28485	108768				181068	
138936	132053	115384	119490	131917	134142	141866	148707	138480
545384	425117		433832			497873		448721
34394		26848		71844	84926		34600	30074
504053	29067	360555	364041	404736	525588	502360	502087	474277
82421			14083		306712	177272		84858
47679	48263		122596		43736	47648		
420470	291390	301195	322305	305321		426433	414868	435644
1318441	1008618	780730	542131		792687	1364032	1395479	1290337
375660	305592	130625	133860	170077	86848	113456	339385	80132
21713		30092	34128		33666	32681	34499	
					97036	27033		26493
102857	147065	123218	1316162	630362	468251	776413	112806	767868
	45507	29278				14218		
63078	50072	46573	56130	46668		35203	38462	37017
6956	121966		110340	9100			116216	
					27428		3108	8794
				57032	68208	44612	65476	
	84955	112563		96807	139996			
	44091			62470	64732	29891	36597	
			1008172	1393480	1228165			
141188	4920	113250		39000	27266	8440	76204	58805
207333				111925	159424	199238	189179	
290122		207777	249272	232220	217045	399700	366468	388690
13572			38901	51980				46782
	602016	95668		366218	489750		724733	725925

280204				77922	110381	126861	125140	70736
65329		67146		59994	54340	79312		69341
48489	171820	873896		900490	589944	586631		910231
6111824		5261897	853658	5061056	5075768	5460446	5170575	5975227
	480392		431804	555948	529792		835248	
16573		22806	35296	40597	40050	26340	35115	37538
146563	120419	113802	64235	42235	116243	103695	137442	63515
1290063	797593	1272751	917969	1264179	1347264	1294739	1377667	1296533
30693	38888	30819		33780		31904	10608	32498
4690805	2475143	4155109	2817657	4210879		4854299	4953183	4837245
132533	120030	123715	98890	137460	122328	133966	154044	157640
276309	1008679	357965			1564951	901365	461304	289054
116458			54151		93429	107106		
	66196		157161	163796	131715			198378
342613	329469	238418		201195		350513		365901
277465	282179	228308	144272	166755	242980	295952	287980	306042
		45449			41080	45376		
966947	303599	942614	503041	875001	897636	901441	963328	992437
5892		656271			878136	1736451	1514642	
41745	4328189	103318	5884		4053943	4781420	4782027	4170040
3463886	827648	3222030	1558379	2986629	2628082	3732892		3151453
75711	65966	84013	58946	58640		61336	70084	68248
	383549	447341	367332	493794	43608			
856089	600731	869947	709873	9568	897056	904480	947388	927334
8952	1364	30307	8164	8684	31456	9547	12748	
61680	1655685	56748	1929736	2584428	2574845	3092481	3065579	3149211
	16488	3612					3756	
1101654	576346	349256			520370	847984	783078	399456
1528446	224946	108781			169315	60324	46572	
17666	20912			17724		19696	16936	
58694	66828		28589	36359	50736	50997		65968
118066		98829	88107	100808	89261	103593	109275	
405308		315596	96073	100544	135462	154795		
	180799	123978	104570	110391	172976	180261	180340	
42424	3456				25545		43272	
690241	558607	675265	623153	596706	681625	644774	712067	706478
6796	24279	24800			22770	6038	8289	
2392698	1652282	1991097	1795737	1790079	2124971	2340740	2409180	2387699
236318	191295	209710	167867	214937	207720	300007		2056
1376	18314	24877	19039	20334	22645	22771		
531547	462124	506281	500405	559286	585787	569408	613236	594728
1817094	1210965	1573651	26376	1534124	1551669	1768350	1881809	1782734

		9268	1592			12200		10576
9338			4228		10141	6717		
		144892	119664	118415	122771			122646
142751	90242	134156	56492	78927	82297	89424	98275	
33137		26388				33554		31929
24306	14836	22490	17938	21113	22551	19502		21505
30259	29861	21518	14996		25907	27036	27396	29320
28132		15276			48540	13691	49043	
	22972	8368	10413	9600	15506	64491	20946	
77210	79784	195241	123252	57885		77031	230532	81923
46709	43076	42725			50472	37065	37461	41326
108263	115552	84554	75588	78007	112468	110082	113933	120426
19304	17007			12397	5652		19406	15016
461718	453561	418445	429431	367496	435842	424707	467125	464102
14397	24314	31349			24095	18122	21304	18455
21476		7308			5288	20309		20312
	60291	61702			41869	78372	53384	27820
1422959	1300783	1172535	1156501	1052264	1258930	1395651	1415560	1408115
12536	10572	14116	12068	9654		15012	15962	5832
		35105	3768		39683	45647		
56244	35012		19184	31348	26306	30973	33224	27745
287394	192529	244083	123571	160494	174985	197370	195857	202876
126018	95822	100757	79498	96865	101750	134563	163536	141078
33184	16730	18887	21812	11346	21897	60170	21398	21195
16517			23648		16013	20400	20336	
305744	322471	295431	314745	337190	357376	344506	362472	376841
	13384	16638			15563	11301	11677	10754
			9144					
1057557	811290	886590	764341	849605	864375	1006708	1035256	1038384
		10316		7948		13152		
292448	241468	223708	199144	278418	217110	367934	424662	420519
19612		10886	7990			17359	17794	19532
						6940		5844
13919		15107		11420		12513	12693	
14123				9671	13963	13913	13488	
	6409				6702	6205	6516	
45475	19346	42010	25924	33789	48707	43869	40001	33932
40668	39585		76140	29963	39306	36185	37303	38815
103703	56958	92737	55817	73489	79951	68974	76941	76306
66352	77928	54304	52607	54401	71044	69608	70686	75167
286289	273700	259174	248084	218088	324592	318544	279040	276531
15008		5804					13341	
400283	14974	221232	10693			16968	25995	28261
806981	745233	4182	651642		704330	771400	5192	785801

92334	82710	81866	48514	53607	86870	57618	58103	60200
46679	37986		11959	10732	36451	8537	8799	
65688	35802	35500	36117	45543		54670	56035	66586
47456	42288		48193				54145	50307
164755	196270	161732	184936	194738	205979	199652	226982	222796
	18033		18204				21682	
585042	507335	498288	431664	474622	495314	554674	605181	579423
13806	12977	12746	10049	9437	12666	14179	10396	
15410	14365	1776	14658	12651	14891		17144	15524
91760		46995					6351	6746
82208	83377		84039	93260	88819	93521	85431	83650
19766			40243			25289		38547
21216		21241	24724			19717		22275
136440	140630	108357	225727	97009	116295	213150	116533	196163
	156799	52397	238390	32458			192374	132927
13734		15978	12147	13293	14555	15406	16469	15787
105935		16267	15165	16408	21449	17236	23460	63027
10070	42031	33525	30936	33130	41206	35947	6339	42501
	13316		10651	5438		11139		11008
161036		139021	141474	119932		147993	158917	154022
21140	28834	24017			26436	23583	22140	23750
499720	428002	431868	411363	358377	453879	484791	520420	488451
39120	89216	18238	54948				26857	23381
100323	52732	16265	47574	11905	3850	4014	120987	75699
	12723		9370		12007	14209	16732	
19352	33635	13296	29636	9214		14568	12065	11059
90218	22306	28768				2172	5239	
9944	232335	82692	192158	110310		109972	18851	40037
22936	14060	13922	12794	13550	23664	13212	13540	14872
	6441	64041		54977			7952	
	62299	58025	8374	34651	10167		5694	7272
19484	15105	6284						13002
51053	33300		25812	7772	28236		65696	48411
47514		12248	129857	3313			20894	37883
14384	136049	30820	52557	29811				19043
21050		20449			22600	18868	20444	
449633	1668713	49330	1325593	164622	7277		930398	1003873
36556	70011	1058583	47024	919545	96054	22676	81836	236666
213794			179548	177832	236220	198921		
2876	12293	53803	5332	68927	72596			19158
	16203	111770		106330		11585		26326
35240	56437	39766	2308	53649	57974	54430	67748	68678
1060450	1010380	57203	20489	7851			1202049	888562
280788	605325	830550	620779	542082	26820		572008	360110

31090	102720	650254	187005	452744	17950	6615	39726	108381
	6724	74819	618521	42213	480684	5698		11449
		20127		56335		57409		15588
	30100	47957		61073				25653
10828	15500	93683	15989	91885	11609	13021		
79851	57102						92964	66602
	17576		9323		12329	32590	35332	
		3674	6621	41148				18453
		9957			11127			3488
328811	250564		57136	13128	38492	39202	486603	376155
11627	12407				3052		20158	22634
50852	453001	62920	299210	243765	1380	19423	72084	101170
45729	68844	27857	50406	22619	219163	23281	29013	22818
		34524		400408	12521	68037		144302
53130	88738	44731	59065	51901	12167		53679	52880
		63980		51345	21922			
	33704	15663	25448	26949		25866	22617	
60144	65670	62600	54132	58328	3040	74010	81050	77217
27810	123814	9193	95707	15286		4238	30873	37363
29200	20763	8504	32230	6377			28487	19353
	9156	17513	13203	235135	40588			146904
20347	6681	64735	58900	45983	137237	7457		12973
24389	54033	18533	38629	6834			46841	28066
13512	8316					18378	15394	14305
		15309		14677				12781
16735	14055		15327	14225	16880		18857	15338
42801		35252	33091	30275	38601	43549	44879	42028
8396	8332	8722	5447	4805	10281	8813		10187
	28777	8069	19556	11342	16656	10723		
31395	28909		22865	23713	26969	31860	7877	32940
7480	5544					10143		7663
9824	4958	8859		7768	7496		8248	12000
16877	12546	15340	15820	11710	15521	17529	19585	21564
	5298			4604	5794	4075		7712
14170		13732	8269	10705	12047	13866	15090	
4428		3976				4511	3567	4231
6942	4214	6607	6948	4246	7664	6899		8078
6502	4966	5057		3920		4236	5727	6308
15120	75561				85669		58410	

L42
114214
52269
12004
27716
597784
33625
17439
70908
124624
99003
19733
20289
90354
563881
47625
347737
129998
600132
53731
117545
17305
66563
32786
69813
46505
28737
547871
181887
44842
39701
264230
67850
58362
328017
82850
48150

20617
116538
805499
24880
276466
50833
188068
94881
35477
631900
241554
18248
135884
671155
93355
31801
118916
60721
45897
432553
28814
110430
149330
177486
54549
35002
103536
2455790
31848
45722
92307
375833

563790
46138
47415

380348
74316

1269245

908349

3647370
55243

215548

46862
45778
65432
14795
586086
445143

1574361
135097
458456
69764
411984
609658
547136
2482624

357095
242407
73943
234857
394376
90543
57042

437924

1560063

157051

25665

127612
411308
101974
93240
752619
19633

68144
182172
136486
520808

199256

403961
1482808

1252199

145684

1439696
52984

383167
52972

122861
71792
3250390
5125942

135126
1562000

136941

115593
188531
389638
323169

969651

12499
3803648
52583
621691
981701

3032992

76045

113477
180408
200284

712078
11520
2356384
290964

672648
1845781

10352
129405
25967
29335
40822
15582
85966
36364
127756
17191
464474
22252
22696
1377766
220761
157760
25360
403758
11740
1040390
454528
14379
6182
53755
42982
86203
77516
290303
15848
4222

96273

55389

249027
2504
621298
13567
15178

86490

105735
128295
1884
74856
44765
12960
163904
28382
532397
23091
91797

11838

28023
14498
6249

12424

18119

661267
46413

102536
17864
1444
985455
371618

101362
129143

10090

68792
4434
21602
10856
3516

91411
27760
932
33637
11404
35410
84215
21867
22427
3716

30976

47742
9945
12067
35736
5516
9289
23808
7251
15743

9161
