# Supplementary material for: Non-Targeted Plasma Lipidomic Profiling in Late Pregnancy and Early Postpartum Stages: An Observational Comparative Study
Source: Metabolites. 2025 Dec 16;15(12):798. doi: 10.3390/metabo15120798 (PMC12734539; doi:10.3390/metabo15120798)
Supplement: Supplementary file 1 [file metabolites-15-00798-s001.zip › Supplementary Table S3.pdf]

**Supplementary Table S3:** List of common molecules (n=277) identified in the plasma of two groups (pregnant and postpartum). The databases used for identification were HMDB and LipidMaps.

| No | m/z | Common molecules | ID | No | m/z | Common molecules | ID |
|---|---|---|---|---|---|---|---|
| 1 | 104.0982 | Choline | HMDB0000097 | 140 | 427.2616 | N-stearoyl arginine | LMFA08020136 |
| 2 | 109.0923 | Phenylenediamine | HMDB0003119 | 141 | 432.2662 | N-stearoyl phenylalanine | LMFA08020093 |
| 3 | 111.1080 | Pyrocatechol | HMDB0000957 | 142 | 434.2642 | N-arachidonoyl glutamic acid | LMFA08020086 |
| 4 | 115.1024 | Dihydrouracil | HMDB0000076 | 143 | 437.2519 | LysoPA (18:1) | LMGP10050008 |
| 5 | 116.0728 | Proline betaine | HMDB0004827 | 144 | 439.2187 | LysoPA (18:0) | LMGP10050005 |
| 6 | 120.0709 | L-Threonine | HMDB0000167 | 145 | 442.3765 | 3-hydroxyoctadecenoylcarnitine CAR 18:1;O | LMFA07070025 |
| 7 | 123.0358 | Erthrithol | HMDB0002994 | 146 | 443.3066 | Cortisol 21- sulfate | LMST05020020 |
| 8 | 125.9763 | Taurine | HMDB0000251 | 147 | 445.2854 | Triacontatetraenoic acid C 30:4 | LMFA01030826 |
| 9 | 129.1175 | Dihydrothymine | HMDB0000079 | 148 | 449.2644 | Fatty acid, C 30:2 | LMFA01020366 |
| 10 | 132.0877 | Leucine | HMDB0000687 | 149 | 452.5016 | Eicosadienoylcarnitine CAR 20:2 | LMFA07070011 |
| 11 | 146.0436 | 3-oxo-5S-amino-hexanoic acid | LMFA01060173 | 150 | 452.6968 | LysoPE (16:1) | LMGP02050010 |
| 12 | 146.0436 | Spermidine | HMDB0001257 | 151 | 453.3173 | Myristyl palmitate | LMFA07010002 |
| 13 | 149.0686 | Mevalonic acid | LMFA01050352 | 152 | 456.2225 | Arachidyl carnitine | LMFA07070052 |
| 14 | 150.0856 | Methionine | HMDB0000696 | 153 | 459.2976 | LysoPA (20:4) | LMGP10050013 |
| 15 | 158.1433 | Tiglylglycine | HMDB0000959 | 154 | 461.2776 | LysoPA (20:3) | LMGP10050028 |
| 16 | 160.0498 | Isovalerylglycine | HMDB0000678 | 155 | 465.3666 | Testosterone glucuronide | LMST05010012 |
| 17 | 162.0382 | L-Carnitine | HMDB0000062 | 156 | 468.3558 | LysoPC 14:0 | LMGP01050012 |
| 18 | 162.1003 | Aminoadipic acid | HMDB0000510 | 157 | 470.2415 | LysoPS (14:0) | LMGP03050009 |
| 19 | 163.0627 | 2-Hydroxyadipic acid | HMDB0000321 | 158 | 484.3541 | O-behenoylcarnitine Car 22:0 | LMFA07070089 |
| 20 | 165.0977 | Phenylpyruvic acid | HMDB0000205 | 159 | 487.3129 | LysoPA (22:4) | LMGP10050020 |
| 21 | 166.0735 | Phenylalanine | HMDB0000159 | 160 | 491.2613 | LysoPA (22:2) | LMGP10050030 |
| 22 | 167.0844 | Phenyllactic acid | HMDB0000779 | 161 | 494.5405 | LysoPC(16:1) | LMGP01050021 |
| 23 | 172.1932 | L-Homocysteine sulfate | HMDB0002238 | 162 | 496.3739 | LysoPC(16:0) | LMGP01050018 |
| 24 | 173.1407 | Capric acid | HMDB0000511 | 163 | 501.3423 | Palmitoleyl linolenate | LMFA07010121 |
| 25 | 174.9915 | Dehydroascorbic acid | HMDB0001264 | 164 | 511.3624 | LysoPG (18:1) | LMGP04050006 |
| 26 | 175.1069 | L-Arginine | HMDB0000517 | 165 | 512.3758 | Cer(d16:0/16:0) | LMSP02020068 |

| | | | | | | | |
|---|---|---|---|---|---|---|---|
| 27 | 177.0421 | Serotonin | HMDB0000259 | 167 | 522.5693 | LysoPC (18:1) | LMGP01050029 |
| 28 | 185.1014 | Phosphorylcholine | HMDB0001565 | 168 | 524.3359 | Cer(d18:2(4E,8E)/14:0(2OH)) | LMSP02010216 |
| 29 | 188.0846 | N1-Acetylspermidine | HMDB0001276 | 169 | 528.2329 | LysoPE (22:5) | LMGP02050058 |
| 30 | 195.1225 | 3-O-Methyl-d-glucose | HMDB0245931 | 170 | 528.3756 | Cer(t18:0/14:0) | LMSP02030051 |
| 31 | 200.2222 | Dodecanamide | HMDB0251566 | 171 | 531.3522 | Linolenyl stearate | LMFA07010148 |
| 32 | 202.2024 | Cysteine-S-sulfate | HMDB0000731 | 172 | 533.3248 | Linoleyl stearate | LMFA07010152 |
| 33 | 203.0382 | Sebacic acid | HMDB0000792 | 173 | 537.2974 | Stearyl stearate | LMFA07010054 |
| 34 | 205.0771 | Trytptophan | HMDB0000929 | 174 | 537.3859 | beta-carotene | LMPR01070001 |
| 35 | 212.2229 | Phosphocreatine | HMDB0001511 | 175 | 540.4093 | Cer(d18:0/16:0) | LMSP02020001 |
| 36 | 213.1329 | Acetyl hydroxytryptamine | HMDB0001238 | 176 | 544.1518 | LysoPC (20:4) | LMGP01050048 |
| 37 | 214.2374 | Indoxyl sulfate | HMDB0000682 | 177 | 550.5964 | LysoPC(20:1) | LMGP01050047 |
| 38 | 216.2175 | Propenoylcarnitine | HMDB0013124 | 178 | 556.4014 | Cer(t18:1(6OH)/14:0(2OH)) | LMSP02010195 |
| 39 | 217.1366 | 3-Hydroxydodecanoic acid | HMDB0000387 | 179 | 560.1602 | LysoPC (20:4;O) | LMGP01050147 |
| 40 | 223.1548 | L-Cystathionine | HMDB0000099 | 180 | 561.3546 | Linoleyl arachidate | LMFA07010164 |
| 41 | 227.1598 | Myristoleic acid | HMDB0002000 | 181 | 562.2100 | Cer(d18:2/18:1) | LMSP02010025 |
| 42 | 228.2531 | Deoxycytidine | HMDB0000014 | 182 | 566.2869 | Cer(d18:1/18:0) | LMSP02010006 |
| 43 | 229.1275 | Traumatic acid | HMDB0000933 | 183 | 568.2966 | Cer(d18:0/18:0) | LMSP02020008 |
| 44 | 230.2320 | Butenoylcarnitine | HMDB0013126 | 184 | 571.5822 | LysoPI (16:1) | LMGP06050009 |
| 45 | 235.1540 | 5-Methoxytryptophan | HMDB0002339 | 185 | 572.3939 | LysoPC(22:4) | LMGP01050124 |
| 46 | 239.1451 | Glycyltyrosine | HMDB0028853 | 186 | 575.3746 | DG(33:4) | LMGL02010368 |
| 47 | 242.2675 | Tetrahydrobiopterin | HMDB0000027 | 187 | 577.3524 | DG(33:3) | LMGL02010019 |
| 48 | 243.1195 | Thymidine | HMDB0000273 | 188 | 581.3191 | DG(33:1) | LMGL02010013 |
| 49 | 245.0633 | Uridine | HMDB0000296 | 189 | 583.2260 | DG(33:0) | LMGL02010012 |
| 50 | 246.1301 | Valeroylcarnitine | HMDB0013128 | 190 | 584.4274 | Cer(d18:0/18:0(2OH)) | LMSP02020030 |
| 51 | 249.1769 | 3,9-hexadecadiynoic acid C 16:4 | LMFA01030491 | 191 | 589.3836 | DG(34:4) | LMGL02010028 |
| 52 | 250.9874 | gamma-Glutamylcysteine | HMDB0001049 | 192 | 595.3426 | DG(34:1) | LMGL02010004 |
| 53 | 252.9843 | Deoxyadenosine | HMDB0000101 | 193 | 597.4329 | DG(34:0) | LMGL02010003 |
| 54 | 256.2815 | Palmitamide | LMFA08010009 | 194 | 600.4213 | Cer(t18:0/18:0(2OH)) | LMSP02030016 |

| | | | | | | | |
|---|---|---|---|---|---|---|---|
| 55 | 258.2619 | Glycerophosphocholine | HMDB0000086 | 195 | 605.3796 | CerPE(d14:1/16:0) | LMSP03020002 |
| 56 | 261.1231 | Androstane 19:0 | LMST02020056 | 196 | 609.2967 | DG(35:1) | LMGL02010029 |
| 57 | 263.2207 | 11-Phenylundecanoic acid C 17:4 | LMFA01140028 | 197 | 610.5348 | Cer(t18:1(6OH)/20:0) | LMSP02010144 |
| 58 | 265.1144 | Phenylacetylglutamine | HMDB0006344 | 198 | 611.3233 | DG(35:0) | LMGL02010025 |
| 59 | 267.2526 | 2,3-Diphosphoglyceric acid | HMDB0001294 | 199 | 619.4279 | CerPE(d14:2/16:0(2OH)) | LMSP03020066 |
| 60 | 269.1918 | DL-Homocystine | HMDB0000575 | 200 | 625.3417 | DG(36:0) | LMGL02010002 |
| 61 | 271.1779 | Estrone | LMST02010004 | 201 | 628.4495 | Cer(t18:0/20:0(2OH)) | LMSP02030017 |
| 62 | 275.2224 | Alfa-androstenol | LMST02020008 | 202 | 629.0961 | CerPE(d14:2/18:1)) | LMSP03020031 |
| 63 | 277.2000 | Stearidonic acid C 18:4 | LMFA01030357 | 203 | 631.3101 | CerPE(d16:2/16:0) | LMSP03020047 |
| 64 | 279.1452 | Estrane-3,17-diol ST 18:0;O2 | LMST02010019 | 204 | 633.4051 | CerPE(d16:1/16:0) | LMSP03020019 |
| 65 | 279.2151 | Alpha-Linolenic acid C 18:3 | LMFA01030152 | 205 | 634.4049 | CerP(d18:1/16:0[2OH]) | LMSP02050015 |
| 66 | 281.1623 | Linoleic acid C C18:2 | LMFA01030120 | 206 | 636.5553 | Cer(d16:2/24:0(2OH)) | LMSP02010093 |
| 67 | 284.3147 | Stearamide | LMFA08010003 | 207 | 638.5607 | Cer(t18:1(6OH)/22:0) | LMSP02010142 |
| 68 | 287.1453 | Dehydro testosteron | LMST02020018 | 208 | 639.3641 | PA(32:5) | LMGP10010061 |
| 69 | 290.2672 | O-adipoylcarnitine | LMFA07070087 | 209 | 641.4382 | DG (38:6) | LMGL02010130 |
| 70 | 293.2144 | 13S-HODTA-C 18:4;O | LMFA02000373 | 210 | 644.4431 | GlcCer(d18:1/12:0) | LMSP0501AA01 |
| 71 | 295.2183 | 13-HOTE - C 18:3;O | LMFA02000029 | 211 | 645.0653 | CerPE(d15:2/18:0) | LMSP03020043 |
| 72 | 297.2244 | 13-HODE- C 18:2;O | LMFA02000035 | 212 | 645.5167 | N-palmitoyl-D-sphingosyl-1-(2-aminoethyl)phosphonate | LMSP04000002 |
| 73 | 298.3293 | Sphingosine 18:2; O2 | LMSP01080010 | 213 | 649.3946 | PA(16:0/16:0) | LMGP10010012 |
| 74 | 301.1486 | 2-Methoxyestrone | LMST02010033 | 214 | 650.2559 | Cer(d18:0/24:1(15Z)) | LMSP02020011 |
| 75 | 304.8507 | Hydroxy testosterone | LMST02020143 | 215 | 660.4391 | GlcAbeta-Cer(d16:0/14:0) | LMSP06030003 |
| 76 | 305.2766 | Arachidonic acid C 20:4 | LMFA01030001 | 216 | 661.0296 | CerPE(d16:1/18:0) | LMSP03020020 |
| 77 | 307.1797 | Eicosatrienoic acid C 20:3 | LMFA01030157 | 217 | 663.4148 | SM(d18:0/13:0) | LMSP03010033 |
| 78 | 309.1904 | Eicosadienoic acid C 20:2 | LMFA01030130 | 218 | 666.5878 | Cer(d18:1/24:0(3OH)) | LMSP02010204 |
| 79 | 311.2207 | Eicosenoic acid C 20:1 | LMFA01030082 | 219 | 669.3663 | DG (40:6) | LMGL02010186 |
| 80 | 313.2276 | Arachidic acid C20:0 | LMFA01010020 | 220 | 672.4711 | GlcCer(d18:1/14:0) | LMSP0501AA26 |

| | | | | | | | |
|---|---|---|---|---|---|---|---|
| 81 | 315.0558 | 12,13-Dihydroxyoleic acid | LMFA02000302 | 221 | 675.4910 | CerPE(d16:1/18:1)(2OH)) | LMSP03020081 |
| 82 | 315.1930 | Progesterone | LMST02030159 | 222 | 677.4284 | CerPE(d16:1/18:0(2OH)) | LMSP03020080 |
| 83 | 317.1870 | Pregnenolone | LMST02030088 | 223 | 679.4567 | DG (40:1) | LMGL02010128 |
| 84 | 318.2966 | Phytosphingosine | LMSP01030001 | 224 | 685.3790 | CerPE(d16:2/20:1) | LMSP03020051 |
| 85 | 319.2634 | 9-hydroxy-Eicosapentaenoic acid C 20:5;O | LMFA01030717 | 225 | 688.4722 | PE (32:2 | LMGP02010108 |
| 86 | 321.2715 | Pregnanediol | LMST02030264 | 226 | 689.6877 | DG (41:3) | LMGL02010212 |
| 87 | 325.2515 | Auricolic acid C 20:2;O | LMFA01050431 | 227 | 693.4191 | DG (42:8) | LMGL02010260 |
| 88 | 326.9949 | 14-hydroxy-11Z-eicosenoic acid | LMFA01050117 | 228 | 694.3539 | Cer(d18:1/26:0(3OH)) | LMSP02010205 |
| 89 | 328.9923 | Phenylalanyltyrosine | HMDB0029007 | 229 | 696.3574 | Cer(t18:1(6OH)/25:0(2OH)) | LMSP02010184 |
| 90 | 331.0373 | 17alpha-hydroxyprogesterone | LMST02030161 | 230 | 701.4499 | CerPE(d16:2/20:1(2OH)) | LMSP03020090 |
| 91 | 333.0428 | 21-hydroxypregnenolone | LMST02030167 | 231 | 703.5173 | SM(d18:1/16:0) | LMSP03010003 |
| 92 | 335.2192 | PGF2a | HMDB0001139 | 232 | 707.4405 | CerPE(d18:0(17Me)/16:0(3OH,15Me)) | LMSP03020095 |
| 93 | 335.3243 | PGA2 | HMDB0002752 | 232 | 709.4068 | DG(42:0) | LMGL02010200 |
| 94 | 336.2934 | Docosatrienoic acid C22:3 | LMFA04000088 | 233 | 713.3910 | DG (44:12) | LMGL02010306 |
| 95 | 337.2131 | Docosadienoic acid C 22:2 | LMFA01170128 | 234 | 713.4949 | CerPE(d14:2/24:1(15Z)) | LMSP03020037 |
| 96 | 338.3235 | 16Z-docosenoic acid C 22:1 | LMFA01031302 | 235 | 716.4925 | PC(P-16:0/16:1)) | LMGP01030026 |
| 97 | 340.2364 | Dodecadienoylcarnitine | LMFA07070124 | 236 | 717.4072 | CerPE(d16:1/22:0) | LMSP03020024 |
| 98 | 340.3719 | Oleoyl glycine | LMFA08020082 | 237 | 721.4475 | DG (44:8) | LMGL02010296 |
| 99 | 341.2452 | Behenic acid C 22:0 | LMFA01010022 | 238 | 723.4839 | DG (44:7) | LMGL02010292 |
| 100 | 342.3573 | Dodecenoylcarnitine | LMFA07070115 | 239 | 725.4988 | DG (44:6) | LMGL02010282 |
| 101 | 343.2675 | Eicosanedioic acid C 20:1; O2 | LMFA01170035 | 240 | 727.4040 | SM(d18:2/18:1) | LMSP03010047 |
| 102 | 343.3372 | N,N,N-trimethyl-sphingosine | LMSP01080057 | 241 | 729.5143 | SM(d18:1/18:1) | LMSP03010029 |

| | | | | | | | |
|---|---|---|---|---|---|---|---|
| 103 | 347.2954 | Corticosterone | LMST02030186 | 242 | 730.4820 | GlcCer(d18:0/18:0) | LMSP0501AA19 |
| 104 | 348.9766 | Dihydrocorticosterone | LMST02030280 | 243 | 732.4944 | GlcCer(t18:1/16:0(2OH)) | LMSP05010042 |
| 105 | 350.9749 | Estrone 3-sulfate | LMST02010043 | 244 | 737.4851 | DG (44:0) | LMGL02010258 |
| 106 | 351.2296 | Pregnanetriolone | LMST02030290 | 245 | 742.5059 | GlcCer(d18:2/18:0(2OH)) | LMSP05010050 |
| 107 | 354.3889 | N-palmitoyl proline | LMFA08020117 | 246 | 744.5179 | GlcCer(d18:1/18:0(2OH[R])) | LMSP05010060 |
| 108 | 355.2231 | PGF2b | LMFA03010025 | 247 | 752.4681 | GlcCer(d18:2/20:1) | LMSP0501AA35 |
| 109 | 355.3568 | 10-oxo-docosanoic acid C22:1;O | LMFA01060139 | 248 | 753.4267 | MGDG (34:3) | LMGL05010056 |
| 110 | 356.3396 | N-palmitoyl valine | LMFA08020120 | 249 | 754.4749 | GlcCer(d18:2)(9Me)/18:1(2OH)) | LMSP0501AA82 |
| 111 | 357.2364 | Tetracosahexaenoic acid C 24:6 | LMFA01030804 | 250 | 756.4931 | GlcCer(d18:2(9Me)/18:0(2OH)) | LMSP0501AA83 |
| 112 | 359.2666 | Tetracosapentaenoic acid C24:5 | LMFA01030820 | 251 | 757.4086 | CerPE(d16:2/24:1(2OH)) | LMSP03020094 |
| 113 | 360.3396 | 2-Hydroxylauroylcarnitine | HMDB0013164 | 252 | 758.5085 | GlcCer(d16:1(15Me)/20:0(2OH) | LMSP05010183 |
| 114 | 363.1979 | Cortisol | LMST02030001 | 253 | 760.5253 | Cer(d18:2/32:0) | LMSP02010178 |
| 115 | 363.2887 | Tetracosatrienoic acid C24:3 | LMFA01031049 | 254 | 765.4722 | PS(18:0/16:0) | LMGP03010888 |
| 116 | 365.2250 | Tetrahydroaldosterone | LMST02030275 | 255 | 766.5104 | Cer(t18:1(6OH)/30:0(2OH)) | LMSP02010183 |
| 117 | 368.4019 | N-oleoyl GABA | HMDB0062335 | 256 | 768.5206 | Cer(t18:0/30:0(30OH)) | LMSP02030042 |
| 118 | 369.2684 | DHEAS | HMDB0001032 | 257 | 776.5121 | 1-O-palmitoyl-Cer(d18:1/16:0) | LMSP02040003 |
| 119 | 369.3512 | Lignoceric acid C 24:0 | LMFA01010024 | 258 | 780.4898 | Cer(t18:0/32:0) | LMSP02030063 |
| 120 | 380.3145 | C18-Sphingosine 1-phosphate | HMDB0000277 | 259 | 782.5064 | GlcCer(d18:2/22:0) | LMSP0501AA37 |
| 121 | 383.1760 | LysoPA (14:0) | HMDB0062321 | 260 | 784.5220 | GlcCer(d18:1/22:0) | LMSP0501AA07 |
| 122 | 387.2326 | Cholesterol | HMDB0000067 | 261 | 786.5326 | GlcCer(d18:0/22:0) | LMSP0501AA21 |
| 123 | 391.3171 | Ketodeoxycholic acid | HMDB0000328 | 262 | 788.5420 | GlcCer(t18:1/20:0(2OH)) | LMSP05010044 |
| 124 | 395.2218 | LysoPA(P-16:0) | HMDB0011154 | 263 | 790.5100 | GlcCer(t15:0(14Me)/22:0(2OH)) | LMSP05010084 |
| 125 | 397.0554 | Pregnenolone sulfate | HMDB0000774 | 264 | 794.5335 | Cer(t18:1(6OH)/32:0(32OH)) | LMSP02010115 |
| 126 | 397.1954 | Cerotic acid C26:0 | HMDB0002356 | 265 | 796.4637 | Cer(t18:0/32:0(32OH)) | LMSP02030044 |
| 127 | 399.2780 | N-Palmitoyltryptamine | HMDB0040815 | 266 | 798.4773 | PI-Cer(d18:0/16:0(2OH)) | LMSP03030030 |
| 128 | 400.3535 | Palmitoylcarnitine | HMDB0000222 | 267 | 801.4326 | SM(d18:1/23:0) | LMSP03010078 |

| | | | | | | | |
|---|---|---|---|---|---|---|---|
| 129 | 401.2561 | 5,6-trans-25-Hydroxyvitamin D2 | HMDB0006721 | 268 | 804.5005 | 1-O-palmitoyl-Cer(d18:1/18:0) | LMSP02040011 |
| 130 | 405.2407 | Cortisol 21-acetate | LMST02030093 | 269 | 806.5042 | Cer(d18:1/34:0(34OH)) | LMSP02010111 |
| 131 | 406.0536 | 12-HETE-GABA | LMFA08020147 | 270 | 808.5184 | PI-Cer(d20:1/16:0) | LMSP03030154 |
| 132 | 407.3119 | 7-Ketodeoxycholic acid | LMST04010184 | 271 | 810.5288 | Acetyl CoA | HMDB0001206 |
| 133 | 408.0499 | N-linolenoyl glutamic acid | LMFA08020214 | 272 | 816.5513 | GlcCer(t18:1/22:0(2OH)) | LMSP05010045 |
| 134 | 409.2234 | LysoPA (16:1) | LMGP10050016 | 273 | 818.5559 | GlcCer(t16:0(15Me)/23:0(2OH)) | LMSP05010078 |
| 135 | 413.2668 | 7-Hydroxypregnenolone sulfate | LMST05020021 | 274 | 820.5259 | Cer(d18:1/35:0(35OH)) | LMSP02010112 |
| 136 | 419.2596 | 7,27-dihydroxycholesterol | LMST04030178 | 275 | 825.4906 | TG (50:5) | LMGL03010056 |
| 137 | 423.2288 | Lyso PA(O-18:1) | LMGP10060006 | 276 | 828.4859 | GlcCer(d18:1/24:0(2OH[R])) | LMSP05010063 |
| 138 | 424.3364 | O-linoleoylcarnitine CAR 18:2 | LMFA07070092 | 277 | 832.5163 | 1-O-stearoyl-Cer(d18:1/18:0) | LMSP02040013 |
| 139 | 425.2279 | Alpha-Tocotrienol | LMPR02020054 | | | | |
